# Self-assembled coordination thioether silver(I) macrocyclic complexes for homogeneous catalysis

**DOI:** 10.3762/bjoc.15.239

**Published:** 2019-10-17

**Authors:** Zhen Cao, Aline Lacoudre, Cybille Rossy, Brigitte Bibal

**Affiliations:** 1 Université de Bordeaux, Institut des Sciences Moléculaires, UMR CNRS 5255, 351 cours de la libération, 33405 Talence, France

**Keywords:** coordination macrocycle, homogeneous catalysis, prochiral, silver complex, thioether ligand

## Abstract

The bis-*ortho*-thioether 9,10-bis[(*o*-methylthio)phenyl]anthracene was synthesized as a *syn-*atropisomer, as revealed by X-ray diffraction. This alkylaryl thioether ligand (L) formed different macrocyclic complexes by coordination with silver(I) salts depending on the nature of the anion: M_2_L_2_ for AgOTf and AgOTFA, M_6_L_4_ for AgNO_3_. A discrete M_2_L complex was obtained in the presence of bulky PPh_3_AgOTf. These silver(I) complexes adopted similar structures in solution and in the solid state. As each sulfur atom in the ligand is prochiral, macrocycles L_2_M_2_ were obtained as mixtures of diastereoisomers, depending on the configurations of the sulfur atoms coordinated to silver cations. The X-ray structures of the two L_2_·(AgOTf)_2_ stereoisomers highlighted their different geometry. The catalytic activity of all silver(I) complexes was effective under homogeneous conditions in two tandem addition/cycloisomerization of alkynes using 0.5–1 mol % of catalytic loading.

## Introduction

Since the early advances in the late eighties [1–10], silver(I) catalysis has been widely exploited based on the versatile redox and soft Lewis acid properties of this coinage metal cation. Silver catalysis has proved its effectiveness for numerous transformations involving unsaturated bond activation (allene, alkyne, alkene) [11–17], radical-based reactions [18–20] and several applications in asymmetric reactions [21–22].

This successful chemistry was usually conducted in the presence of commercially available inexpensive salts (AgOTf, AgNO_3_) and eventually a (chiral) ligand. Such silver(I) complexes were prepared by using (bi)pyridine [19,23–26], phosphine [22,27–30], ditopic N/P [22] ligands and a few S/P and S/N ones [31–34]. None of the silver(I) catalysts based on sulfur ligands were reported so far, although alkyl thioethers are soft σ-donor ligands such as crown thioethers that were largely developed as macrocyclic ligands for silver(I) [35–43]. Interestingly, depending on their design, these known silver(I) complexes can be discrete species [35–38], coordination driven supramolecules [35,42] or coordination polymers [39–40,42]. To the best of our knowledge, silver crown thioether complexes were never reported as catalysts.

Recently, we described a flexible bis-dialkyl thioether ligand for gold(III) chloride whose photoreduction to gold(I) was fast and controlled [44–45]. These gold complexes at different oxidation states showed efficient catalytic properties, that were highlighted in a one-pot cascade synthesis of 4*H*-benzoquinolizin-4-one. Now, we propose to investigate a rigid bis-alkylaryl thioether ligand to control the directionality and spatial orientation of its coordination to silver(I). Besides, 9,10-diphenylanthracenes (DPA) with *ortho*-substituted 9- and 10-aryl groups can exist as *syn-* or *anti-*atropisomers whose rotational barrier [46–47] ranges from 21 (*ortho*-H) to 25–29 kcal/mol (*ortho*-CH_3_). A few *ortho*-substituted DPA atropisomers have been described as potent molecular switches [48–49], building-blocks for self-assembled capsules [50–51] or optical materials [52–54]. However, due to their partially frozen structure, *syn-*isomers of DPA are ideal candidates for directional metal coordination. Herein, a new *syn*-atropisomer of 9,10-DPA *ortho*-substituted by two thioethers is exploited as a ligand for silver(I) salts. The impact of this bis-thioether ligand on silver(I) homogeneous catalysis is evaluated in two tandem addition/cycloisomerization reactions of alkynes.

## Results and Discussion

### Synthesis of silver(I) complexes

**Scheme 1:**
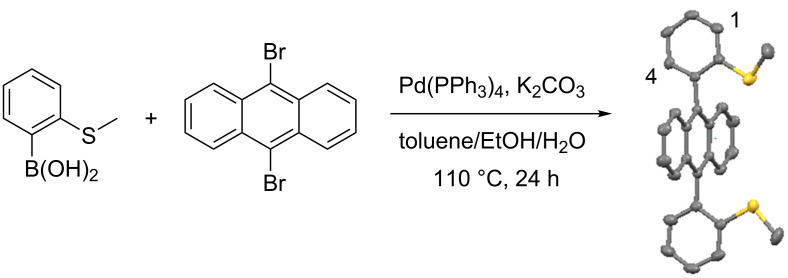
Synthesis of ligand **1**, as its *syn-*atropisomer.

Ligand **1** was synthesized in one step, from commercially available 9,10-dibromoanthracene and 2-(methylthio)phenylboronic acid, using a Suzuki–Miyaura cross-coupling reaction. Notably, the yield was low (26%) [55], and the X-ray analysis of monocrystals revealed the *syn-*conformation of **1** (Scheme 1). A variable temperature ^1^H NMR (VT-NMR, Figures S10–12 in Supporting Information File 1) experiment conducted on *syn-***1** in the range of –30 to 110 °C in C_2_D_2_Cl_4_ showed the broadening of proton signals, due to the restricted rotation of the 9,10-aryl substituents in respect to the anthracene core, without any indication of a *syn-*to-*anti* isomerization.

Four silver(I) complexes **1a**–**d** were prepared in excellent yield (77–92%) by mixing the ligand and the following silver salts in a 1:1 ratio in dichloromethane at room temperature: AgOTf for **1a**, AgOTFA for **1b**, AgNO_3_ for **1c** and PPh_3_AgOTf for **1d**.

### Crystallographic structures of silver complexes

Single crystals of **1a**, **1c** and **1d** suitable for X-ray diffraction analysis were grown from the slow diffusion of hexane into a solution of each complex in dichloromethane. Interestingly, each prochiral sulfur atom of the ligand becomes asymmetric by coordination to silver(I). In absence of any chiral source, the complexes were obtained either as nonchiral coordination products with a center or an axis of symmetry (**1a** and **1c**, with (*R*,*S*)–**1** ligands) or as a racemic mixture (**1d**, with (*R*,*R*)–**1** or (*S*,*S*)–**1** ligands).

**Figure 1:**
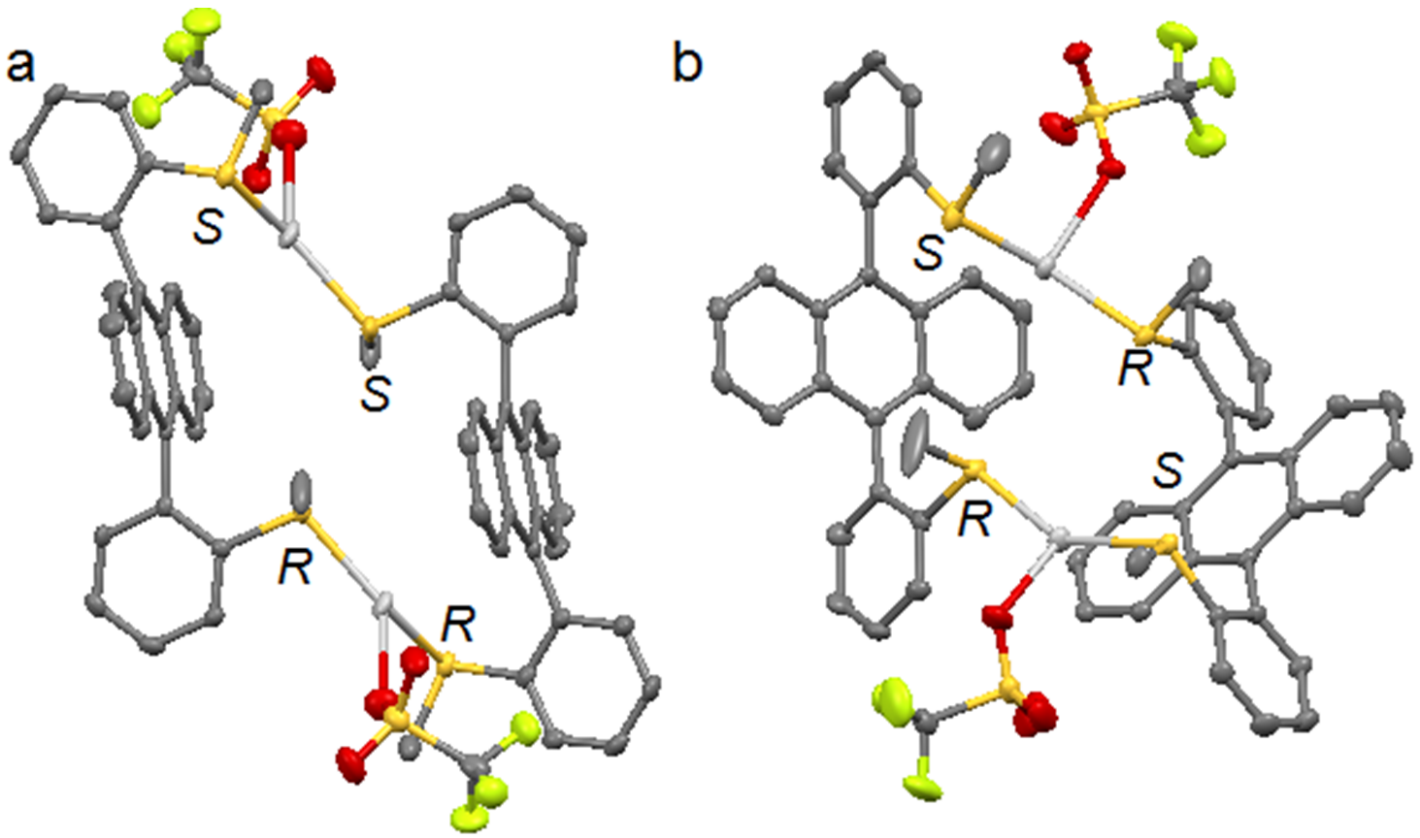
X-ray structures of complex **1a**, as two diastereoisomeric macrocycles (*R*,*S*-**1**)_2_·(AgOTf)_2_ with ligands assembled in: (a) a head-to-head fashion and, (b) a head-to-tail mode. Hydrogen atoms are omitted for clarity.

The X-ray diffraction of monocrystals **1a** revealed the formation of (*R*,*S*–**1**)_2_·(AgOTf)_2_ macrocycles driven by silver(I) coordination (Figure 1). The two ligands are facing through the coordination of one *syn-*thioether group to the same silver cation. Two different crystals were isolated and highlighted the two possible arrangements of the ligands that led to different diastereoisomeric macrocycles (Figure 1a,b). In Figure 1a, each silver cation was coordinated to two sulfur atoms with the same configuration (named head-to-head coordination mode for ligands) meanwhile in Figure 1b, each Ag(I) cation was coordinated to two sulfur atoms with (*R*)- and (*S*)-configuration, respectively (head-to-tail ligand coordination mode). These diastereoisomeric crystals presented a slightly different spatial arrangement.

The head-to head macrocycle 1a had a parallelepiped shape (Figure 1a): the interplanar distance between two anthracenes was ca. 6.31 Å and the dihedral angle between the anthracene core and its 9,10-aryl substituents was 89° and 104°, respectively. The head-to-tail macrocycle **1a** adopted a V-shape (Figure 1b): the angle between the planes of the two anthracenes was 73.2° and the dihedral angles between the aryl substituents and the anthracene plane ranged between 77° and 87°, respectively.

In solution, the two diastereoisomeric macrocycles coexisted, possibly in different ratios as the chemical shift of protons on the 9,10-phenyl and anthracene moieties were found slightly different for three different batches of silver complexes prepared by the same procedure (see Supporting Information File 1, Figure S9).

**Figure 2:**
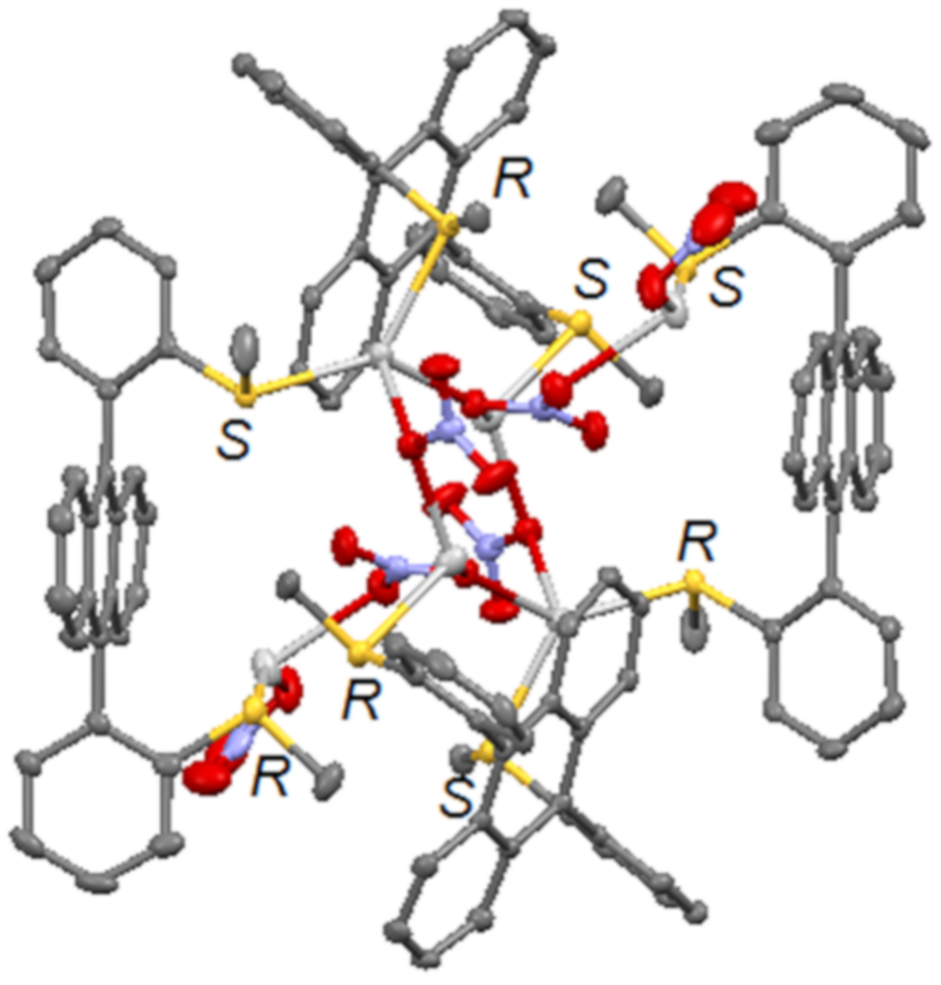
X-ray structure of complex **1c**, as a (*R*,*S*-**1**)_4_·(AgNO_3_)_6_ cage with three nitrate anions as coordinating bridges. Hydrogen atoms are omitted for clarity.

The complex formed with AgNO_3_ had a different stoichiometry, due to the multidentate nitrate anions. The X-ray structure of **1c** appeared as a large (*R*,*S*-**1**)_4_·(AgNO_3_)_6_ complex with three nitrate anions as coordinating bridges between the two symmetric M_3_L_2_ units (Figure 2). The resulting coordinated macrocycle M_6_L_4_ had a globular shape that displayed the polyaromatic ligands towards the exterior and fully encapsulated two nitrate anions and the silver(I) cations. This silver nitrate complex was thus soluble in chlorinated solvents.

**Figure 3:**
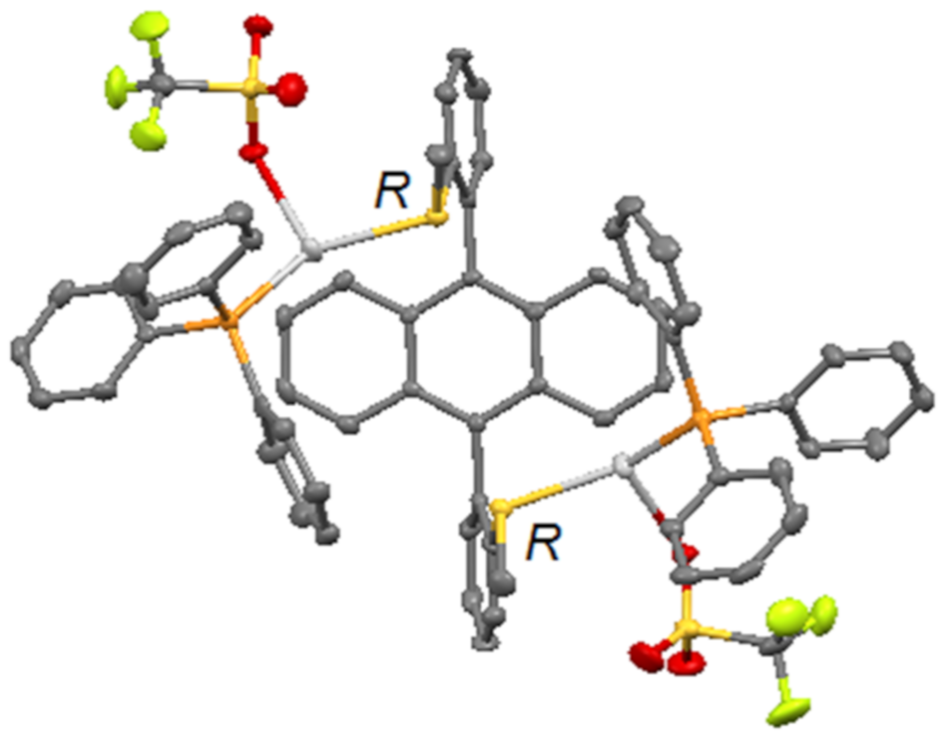
X-ray structure of complex **1d**, as a racemic mixture of (*R*,*R*)- and (*S*,*S*)-(*syn-***1**)·(PPh_3_AgOTf)_2_.

In the presence of the bulky triphenylphosphine silver triflate salt, a monocoordination occurred between Ag(I) and each sulfur atom of ligand **1** leading to a discrete complex (*syn-***1**)·(Ph_3_PAgOTf)_2_, as revealed by ^1^H NMR and X-ray (Figure 3). The steric hindrance also induced the access to a racemic mixture of (*R*,*R*)- and (*S*,*S*)-complexes where triphenylphosphine groups were located at the opposite sides of the anthracene core.

The crystallographic structures of **1a**, **1c** and **1d** showed that both ligand *syn-***1** and the nature of silver anion impacted on the stoichiometry of coordination complexes. Atropisomer **1** directed the self-assembly towards the same half-space (regarding the anthracene core). The triflate anion lead to a [2 + 2] macrocycle meanwhile the more coordinating nitrate anion induced the formation of a large globular macrocycle M_6_L_4_. A bulkier silver salt favored a mono-coordination on each binding site.

### Silver(I) complexes in solution

Surprisingly, several batches of each complex **1a**–**d** were compared by ^1^H NMR (CDCl_3_, 2 mM) (see Supporting Information File 1, Figure S9) and revealed slight variations in the chemical shifts that might originate from the presence of several species in solution, such as self-aggregates, mixture of conformers, mixture of diastereoisomers or complexes with different stoichiometry.

**Figure 4:**
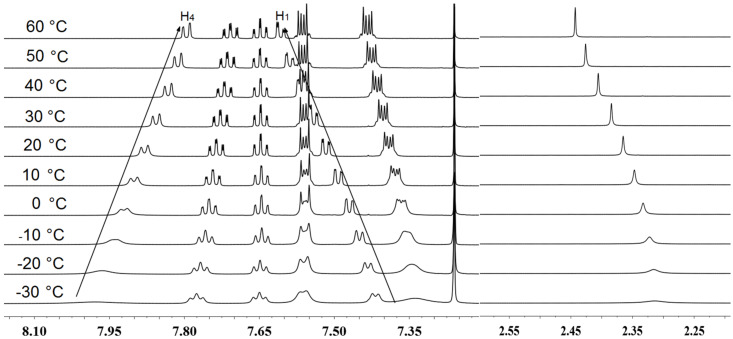
Variable temperature ^1^H NMR of complex **1a** in CDCl_3_ (7 mM) from −30 °C to 60 °C.

To investigate the possible self-aggregation of complexes **1a**–**c** (7 mM), VT NMR experiments were conducted between –30 °C and 60 °C in CDCl_3_. For complex **1a**, the aromatic protons were shifted for 0.1 to 0.2 ppm meanwhile the methyl of the thioether group was shifted for 0.12 ppm (Figure 4). The two most affected aromatic protons were located on the 9,10-substituents, the one adjacent to SMe group (H1) and the one closest to the anthracene core (H4). Similar changes on VT NMR were observed for complex **1b** and **1c** (see Supporting Information File 1, Figures S11 and 12), with chemical shift variations in the range of 0.1 to 0.3 ppm for **1c**. The little differences observed on ^1^H NMR spectra at 60 °C accounted for a slight change in the geometry of the complexes probably due to the thermal motion, and not to the dissociation of self-aggregates. In the temperature range of –30 to 60 °C, none dissociated free ligand was observed, thus confirming that coordinated macrocycles **1a**–**c** were thermally stable. At room temperature, the slight different ^1^H NMR spectra may account for the existence of several conformations for these constrained coordinated architectures, which still have a certain degree of freedom, that is required for further catalytic properties.

Diffusion-ordered spectroscopy (DOSY) ^1^H NMR was also used on complexes **1a**–**d** (5 mM) in CDCl_3_ (Supporting Information File 1, Table S7). Each complex appeared as a unique species whose diffusion coefficient (*D*) can be fitted. So the presence of species with different coordination modes can be excluded. Unexpectedly, the diffusion constants of both complexes **1a** and **1b** were disparate (0.6–1.9 × 10^−9^ m^2^/s) depending on the batches meanwhile the *D* values of complexes **1c** and **1d** at 5 mM were similar (0.80–0.93 × 10^−9^ m^2^/s) and not depending on the batch. The nonreproducibility of DOSY experiments for **1a** and **1b** might originate from the uncontrolled formation of different diastereoisomers by coordination which had different geometries. Thereafter, the silver(I) complexes are discussed with the proposed stoichiometry from X-ray and NMR data, without accounting for stereochemistry: M_2_L_2_ for AgOTf and AgOTFA complexes (**1a**,**b**), M_6_L_4_ for AgNO_3_ one (**1c**) and M_2_L complex with PPh_3_AgOTf (**1d**).

Finally, the photophysical properties of ligand *syn-***1** (20 μM) and complexes **1a**–**d** (30 μM) were evaluated in dichloromethane (Supporting Information File 1, Figures S4–S8). The UV–visible and fluorescence emission spectra (λ_exc_ = 345 nm) of ligand and complexes were similar and correspond to those of 9,10-diphenylanthracene [56–57]. The silver(I) coordination on the ligand and the formation of supramolecular systems does not seem to affect the spectroscopic properties of the 9,10-diphenylanthracene system.

### Silver catalysis

As silver(I) salts exhibit a high alkynophilicity [11–17], the new complexes **1a**–**d** were evaluated as homogeneous catalysts in two tandem addition/cycloisomerization reactions using alkynes **2** and **3**.

2-Alkynylbenzaldehyde **2** [58–59] was chosen as the first model substrate for a cyclization reaction in the presence of methanol as a second nucleophile. This tandem addition/cycloisomerization was previously described in high yields (>95%) using 5 mol % catalyst loadings starting from 2-(alkynyl)quinoline-3-carbaldehyde [60–61] with AgOTf catalyst and starting from 2-alkynylbenzaldehyde derivatives [62] in the presence of a macrocyclic pyridine-tetraaza complex of Ag(I) as a catalyst. In our control experiment, alkyne **2** was converted into product **4** in high yield (88 %) using AgOTf at 5 mol % (Table 1, entry 1). Interestingly, 1-methoxyisochromene **4** can also be isolated in 89% yield, using a AgOTf catalytic loading of 1 mol % (Table 1, entry 2). All silver complexes **1a**–**d** (1 mol %) efficiently catalyzed the intramolecular cyclization with 65–92% yields (Table 1, entries 3–7). To our delight, the transformation also reached 92% yield by employing **1a** at 0.5 mol % (Table 1, entry 4). Compared to literature [60–62], catalyst **1a** is effective for the tandem cyclization of 2-alkynylbenzaldehyde **2** at lower catalyst loadings and under milder conditions (20 °C, full conversion after 12 h). As previously observed for inorganic Ag salts [60], the catalyst efficiency for this cyclization slightly depends on its anion nature (**1a** > **1c** > **1b** > **1d**).

**Table 1:** Addition/cycloisomerization of alkyne **2**.^a^

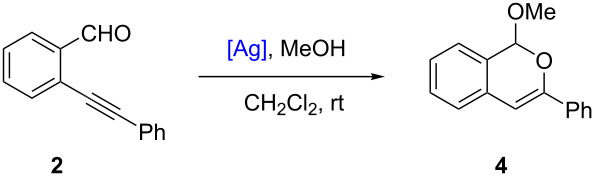			

Entry	[Ag] catalyst	Loading (mol %)	Yield^b^ (%)

1	AgOTf	5	88
2	AgOTf	1	89
3	**1a**	1	92
4	**1a**	0.5	92
5	**1b**	1	73
6	**1c**	1	85
7	**1d**	1	65

^a^Unless specified, all the reactions were carried out in dry CH_2_Cl_2_ at room temperature for 12–16 h with alkyne **2** (30 mg, 0.15 mmol), MeOH (0.45 mmol) and a silver(I) catalyst (0.5–5 mol %). ^b^Isolated yield.

To further demonstrate the catalytic properties of silver(I) complexes **1a**–**d**, we investigated their performance in the cyclization [63] of alkynone **3** [63–65] in the presence of benzylamine, as a nucleophile (Table 2) that lead to substituted pyrrole **5**. This tandem condensation/cycloisomerization was previously reported in 78% yield using AgOTf at 5 mol % (reaction time 3.5 h, 50 °C) [63]. Noteworthy, at 50 °C the transformation occurs in 35% yield without any catalyst (Table 2, entry 1). In our hands, using AgOTf (2.5 mol %), the product was obtained in 73% yield meanwhile 67–76% yield were reached when silver complexes **1a**–**d** at 1 mol % were employed (Table 2, entries 2–6). Interestingly, a lower catalytic loading of 0.5 mol % allowed the isolation of 73% of pyrrole **5** in the presence of catalysts **1a** and **1b** (Table 2, entries 7 and 8). Under the same conditions, the catalytic efficiency of **1c** and **1d** was slightly lower (64–67 % yield) but similar to AgOTf at 2.5 mol % (Table 2, entries 9 and 10). For this second tandem model cyclization, the effect of the anion on the catalysts’ efficiency was weak. Finally, this second model addition/cycloisomerization was successfully catalyzed by silver complexes **1a**–**b** also at lower loadings (0.5 mol %) than AgOTf.

**Table 2:** Condensation/cycloisomerization of alkyne **3**.^a^

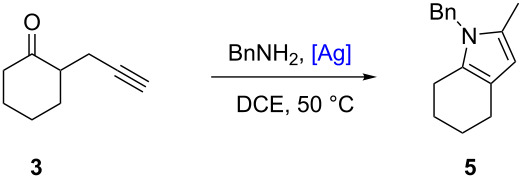			

Entry	[Ag] catalyst	Loading (mol %)	Yield^b^ (%)

1	–	–	35
2	AgOTf	2.5	73
3	**1a**	1	76
4	**1b**	1	67
5	**1c**	1	72
6	**1d**	1	76
7	**1a**	0.5	73
8	**1b**	0.5	73
9	**1c**	0.5	67
10	**1d**	0.5	64

^a^Unless specified, all the reactions were performed at 50 °C under argon atmosphere in dry 1,2-dichloroethane for 12–16 h, in the presence of alkyne **3** (27 mg, 0.2 mmol), benzylamine (0.3 mmol) and a silver(I) catalyst (0.5–2.5 mol %). ^b^Isolated yield.

## Conclusion

9,10-Diphenylanthracene with two *ortho*-substituted thioether functional groups is an attractive scaffold which allowed us to design and prepare a stable *syn-*atropisomer ligand **1** which can direct coordinate towards the same half-space. Three macrocyclic silver complexes were then synthesized and their coordination modes revealed by X-ray diffraction were depended on the nature of the anion, i.e., M_2_L_2_ for ^–^OTf/^–^OTFA and M_6_L_4_ for NO_3_^–^. With prochiral thioether groups, M_2_L_2_ macrocycles were obtained as mixtures of diastereoisomers due to the two possible arrangements of the coordinated ligands (head-to-head or head-to-tail) resulting into different spatial arrangements. In solution, the architectures of silver(I) complexes with ligand *syn-***1** seemed to be similar to the solid-state structures. The silver(I) complexes were evaluated as homogeneous catalysts in two tandem addition/cycloisomerization reactions on model alkynes to give the expected cyclization products in excellent yields, also by using 0.5 mol % catalytic loading, with efficiencies similar to those reported in the literature [60–63] with inorganic silver salts and complexes employed at higher loadings (2.5–5 mol %). The use of original and effective silver complexes might be a way to lower the catalytic loading in silver-catalyzed transformations and opens perspectives to the design of new asymmetric ligands.

## Experimental

**General procedure for the synthesis of silver(I) complexes 1a–d**: To a solution of ligand **1** (15 mg, 0.0355 mmol) in anhydrous CH_2_Cl_2_ (1 mL) was added the corresponding silver salt (0.0355 mmol, 1.0 equiv) under argon atmosphere at room temperature. The mixture was stirred for 4 h. The clear solution was concentrated to ca. 0.3 mL and diethyl ether (2.0 mL) was slowly added to afford a precipitation. After filtration, the isolated solid was washed with diethyl ether and dried under vacuum.

**General procedure for the acetalization/cycloisomerization of alkyne 2**: An oven-dried Schlenk tube was charged with the silver(I) catalyst (0.5–5 mol %), then degassed and backfilled with argon for three times. A solution of alkyne **2** (30 mg, 0.15 mmol) in anhydrous CH_2_Cl_2_ (1 mL) and dry MeOH (18 µL, 0.45 mmol) were successively added. The mixture was stirred at room temperature for 12 h. The solution was concentrated and the crude residue was purified by column chromatography on silica gel (eluent: petrol ether/ethyl acetate 50:1) to obtain 1-methoxyisochromene **4**.

**General procedure for condensation/cycloisomerization of alkyne 3**: A dry Schlenk tube charged with silver catalyst (0.5–2.5 mol %) was degassed and backfilled with argon for three times. A solution of alkyne **3** (27 mg, 0.2 mmol) in anhydrous CH_2_Cl_2_ (1 mL) and benzylamine (33 µL, 0.3 mmol) were successively added. The mixture was stirred at 50 °C for 12 h. The solution was concentrated under reduced pressure and the crude product was purified by column chromatography on silica gel (eluent: petrol ether/ethyl acetate 70:1) to obtain pyrrole **5**.

## Supporting Information

File 1 Synthesis and characterization data of ligand and silver complexes, details of X-ray structures.

File 2 Crystallographic structures CCDC 1883532, 1883535, 1883536, 1883538 and 1883674 of ligand **1** and silver complexes **1a**–**d** (.cif files).
