# Supplementary material for: Self-assembled coordination thioether silver(I) macrocyclic complexes for homogeneous catalysis
Source: Beilstein J Org Chem. 2019 Oct 17;15:2465–72. doi: 10.3762/bjoc.15.239 (PMC6808213; doi:10.3762/bjoc.15.239)
Supplement: File 1 — Synthesis and characterization data of ligand and silver complexes, details of X-ray structures. [file Beilstein_J_Org_Chem-15-2465-s001.pdf]

BEILSTEIN JOURNAL OF ORGANIC CHEMISTRY

# Supporting Information

for

# Self-assembled coordination thioether silver(I) macrocyclic complexes for homogeneous catalysis

Zhen Cao, Aline Lacoudre, Cybille Rossy and Brigitte Bibal

*Beilstein J. Org. Chem.* **2019,** *15,* 2465–2472. doi:10.3762/bjoc.15.239

# Synthesis and characterization data of ligand and silver complexes, details of X-ray structures

License and Terms: This is a supporting information file under the terms of the Creative Commons Attribution License (http://creativecommons.org/licenses/by/4.0). Please note that the reuse, redistribution and reproduction in particular requires that the authors and source are credited.
The license is subject to the *Beilstein Journal of Organic Chemistry* terms and conditions: (https://www.beilstein-journals.org/bjoc)

# Self-Assembled Coordination Thioether Silver(I) Macrocycles for Homogeneous Catalysis

Z. Cao, A. Lacoudre, C. Rossy, B. Bibal

## Contents


| | |
|---|---|
| General information | S2 |
| Synthesis of ligand *syn*-**1** | S4 |
| Synthesis of silver(I) complexes **1a–d** | S5 |
| Details of X-ray structures | S9 |
| Photophysical property of ligand and silver(I) complexes | S15 |
| $^{1}H$ NMR study | S18 |
| Silver catalysis | S27 |
| Infrared spectroscopy | S29 |
| NMR spectra | S34 |
| References | S51 |

## General information

Unless specified, all the reactions were performed under argon atmosphere. All commercially avaible reagents were used without further purifications: AgOTf (Sigma-Aldrich), triphenylphosphinegold(I) chloride (Sigma-Aldrich), 2-(methylthio)benzene boronic acid (Alfa Aesar), 9,10-dibromoanthracene (TCI). Dichloromethane was purified and dried according to standard methods prior to use.
Silver trifluoroacetate[1] and triphenylphosphinesilver triflate[2] were prepared according to the literature.

For the assignment of NMR signals, the following abbreviations were employed: ‘br’ for broad peaks, ‘m’ for multiple peaks, ‘d’ for doublet, ‘t’ for triplet, ‘q’ for quartet, ‘dd’ for double doublet. The protons belonging to the anthracene core were named ‘Anthr-H’ meanwhile those of the 9,10-phenyl substituents were coinded ‘Benz-H’.

The UV–vis spectroscopy was proceeded on a VARIAN 5000 UV-vis-Nir spectrophotometer and the fluorescence emission spectra were done using a Fluoromax-4 spectrofluorometer. The infrared spectroscopy was acquired using a spectrum 100 FT-IR spectrometer.

Crystallographic data of complexes **1a**–**d** were acquired at CESAMO (UMR 5255) on a Bruker APEX 2 DUO. A single crystal was mounted and immersed in a stream of nitrogen gas [$T$ = 150(2) K]. Data were collected, using a micro-focus sealed tube of Mo Kα radiation (k = 0.71073 Å) on a KappaCCD diffractometer. Data collection and cell refinement were performed using APEX2 2013.10-0 (Bruker AXS Inc.), and SAINT v8.34A (Bruker AXS Inc.). Data reduction was performed using SAINT v8.34A (Bruker AXS Inc.). Correction for absorption was performed using multi-scan integration as included in SADABS V2012/1 (Bruker AXS). Structure solutions were found by charge flipping methods SUPERFLIP[3a] EDMA[3b] and refined with SHELXL.[3c]
Crystallographic data of compound **1** were collected at Biophysical and Structural Chemistry plateform (BPCS) at IECB, CNRS UMS3033, Inserm US001, Bordeaux University, with a FR-X Rigaku diffractometer with rotating anode and monochromatic Cu-Kα radiation (λ = 1.54187 Å) and a Dectris Pilatus 200K detector or a R-Axis Rapid Rigaku MSC diffractometer with monochromatic Cu Kα radiation (λ = 1.54187 Å) and a

curved image plate detector. The unit cell determination and data reduction were performed using the Crystal Clear program suite[4a] on the full set of data. The structure was solved by direct methods and refined using Shelx suite of programs[3c] in the integrated WinGX system[4b]. The positions of the H atoms were deduced from coordinates of the non-H atoms and confirmed by Fourier synthesis. The non-H atoms were refined with anisotropic temperature parameters. H atoms were included for structure factor calculations but not refined.

Figures were obtained using Mercury software[4c]. Crystallographic data have been deposited at the Cambridge Crystallographic Data Centre.

## Synthesis of Ligand *syn* 1

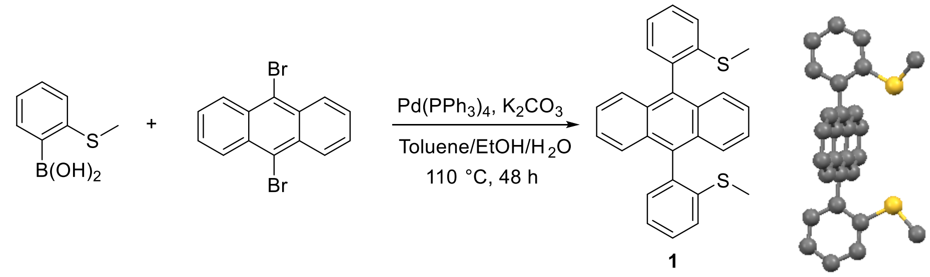


A Schlenk tube was charged with 2-(methylthio)benzeneboronic acid (150 mg, 0.89 mmol), 9,10-dibromoanthracene (120 mg, 0.36 mmol), tetrakis(triphenylphosphine)palladium (21 mg, 5 mol %) and potassium carbonate (248 mg, 1.8 mmol). The mixture was degassed and flushed with argon for three times. Then the degassed solvent toluene/ethanol/water (6 mL, 4:1:1) was added. The resulting mixture was stirred at 110 °C for 48 h. After cooling to room temperature, the suspension was filtered through a pad of celite and washed with dichloromethane. The filtrate was dried over magnesium sulfate, then concentrated and purified by column chromatography on silica gel (eluent: petrol ether/ ethyl acetate, 75: 1) to afford *syn*-atropisomer **1** as a light yellow solid (180 mg, 26% yield).

M. p. 252°C; FTIR (KBr): $\tilde{\nu}$ = 3426, 3049, 2916, 1583, 1468, 1434, 1383, 1269, 1256, 1163, 1070, 1023, 967, 941, 767, 752, 698, 661, 610 $cm^{-1}$

$^{1}H$ NMR (300 MHz, $CDCl_3$) δ 7.52-7.59 (m, 6H, Anthr-H and Benz-H), 7.44 (d, *J* = 7.6 Hz, 2H, Benz-H), 7.31-7.40 (m, 8H, Anthr-H and Benz-H), 2.25 (m, 6H, $SCH_3$).

$^{13}C$ NMR (75 MHz, $CDCl_3$) δ 140.1, 137.0, 135.3, 131.6, 130.0, 128.6, 121.7, 125.5, 124.6, 15.3

HRMS (FD): *m/z* calculated for $C_{13}H_{19}O_4BrNa$ $[M]^{+\bullet}$: 422.1163; found: 422.1180.

The *syn* configuration was confirmed by X-ray diffraction on a monocrystal obtained by the diffusion of hexane into a solution of **1** (3 mg) in dichloromethane (0.3 mL).

## Synthesis of silver complexes 1a–d

Synthesis of complex **1a** = ((*R,S*)–**1**)$_2$.($AgSO_2CF_3$)$_2$

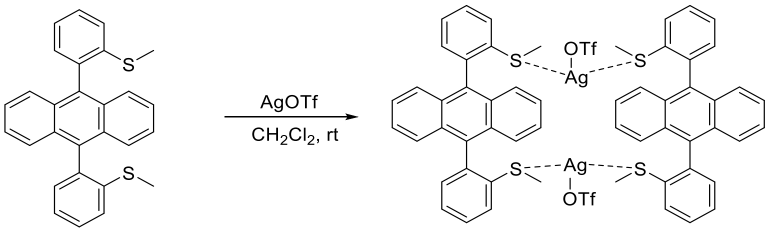


**1a**

To a solution of *syn*-**1** (15 mg, 0.0355 mol) in dichloromethane (1 mL) was added silver trifluoromethanesulfonate (9.1 mg, 0.0355 mmol) under argon atmosphere at room temperature. The mixture was stirred at room temperature for 4 h. Then the solution was concentrated to ca. 0.3 mL and diethyl ether (2 mL) was added to afford a white precipitate. The solid was filtered, washed with diethyl ether and dried under reduced pressure to afford product **1a** (22 mg, 92% yield).

M. p. 246 °C; FTIR (KBr): $\tilde{\nu}$ = 3440, 3057, 2927, 1620, 1474, 1437, 1383, 1279, 1243, 1221, 1162, 1026, 773, 753, 635 cm$^{-1}$

$^{1}$H NMR (300 MHz, $CDCl_3$) δ 7.86 (d, *J* = 7.9 Hz, 4H, Benz-H), 7.73 (td, *J* = 7.4, 1.5 Hz, 4H, Benz-H), 7.65 (td, *J* = 7.4, 1.2 Hz, 4H, Benz-H), 7.52-7.58 (m, 12H, Anthr-H and Benz-H), 7.36-7.42 (m, 8H, Anthr-H) 2.38 (s, 12H, $SCH_3$).

$^{13}$C NMR (75 MHz, $CDCl_3$) δ 138.3, 134.4, 133.4, 131.7, 130.6, 130.1, 129.6, 128.6, 126.9, 125.4, 20.4.

$^{19}$F NMR (282 MHz, $CDCl_3$) δ (ppm) -77.4.

Monocrystals (needles) were obtained by the slow diffusion of hexane into a solution of complex **1a** (1.5 mg) in $CH_2Cl_2$ (0.2 mL). Two different crystal structures ((*R,S*)-**1**)$_2$.($AgSO_2CF_3$)$_2$ involving the coordination of different asymmetric sulfur atoms on silver(I) were obtained:

a) the two sulfur atoms coordinated to silver(I) presented the same configuration (either *(R)* or *(S))*. The two ligands **1** are facing head-to-head (Fig. S1a);

b) the two sulfur atoms coordinated to silver(I) presented a *(R)* and a *(S)* configuration, respectively. The two ligands **1** are placed in a head-to-tail way (Fig. S1b)

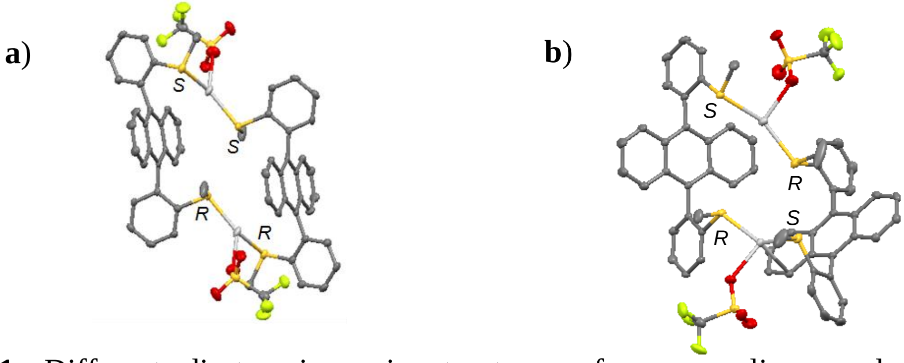


**Figure S1**. Different diastereoisomeric structures of macrocyclic complex [(*R,S*)-**1**)$_2$·($AgSO_2CF_3$)$_2$]: **a**) Ligands are placed in a head-to-head fashion, with two *(R)* or two *(S)* stereogenic sulfur atoms coordinated to each silver(I). This macrocycle possesses a center of symmetry; **b**) Ligands are placed in a head-to-tail way, with one *(R)* and one *(S)* stereogenic sulfur atoms coordinated to each silver(I); this macrocycle has a center of symmetry.

Synthesis of complex **1b** = (**1**)$_2$·$(AgCO_2CF_3)_2$

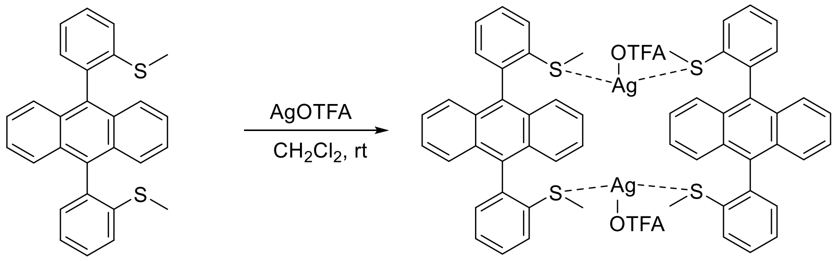


**1b**

To a solution of *syn*-**1** (6 mg, 0.014 mol) in dichloromethane (1 mL) was added silver trifluoroacetate (3.1 mg, 0.014 mmol) under argon atmosphere at room temperature. The mixture was stirred at ambient temperature for 4 h. Then the solution was concentrated to *ca*. 0.2 mL and diethyl ether (2 mL) was added. A light yellow solid was formed after 5 min. The precipitate was filtered, washed with diethyl ether and dried under reduced pressure to afford complex **1b** (7 mg, 78% yield).

M. p. 235 °C; FTIR (KBr): $\tilde{\nu}$ = 3426, 3052, 2919, 1620, 1473, 1434, 1384, 1299, 772, 754, 664 $cm^{-1}$

$^1H$ NMR (300 MHz, $CDCl_3$) δ 7.61-7.67 (m, 4H, Benz-H), 7.55-7.59 (m, 8H, Anthr-H), 7.45-7.55 (m, 12H, Benz-H), 7.36-7.41 (m, 8H, Anthr-H), 2.40 (s, 12H, $SCH_3$).

$^{13}C$ NMR (75 MHz, $CDCl_3$) δ 137.2, 136.5, 134.6, 131.7, 130.1, 129.8, 129.3, 126.6, 126.5, 125.8, 125.7, 17.1.

$^{19}F$ NMR (282 MHz, $CDCl_3$) δ -73.7 ppm.

Synthesis of complex **1c** = [(*R*,*S*–**1**)$_4$·($AgNO_3$)$_6$]

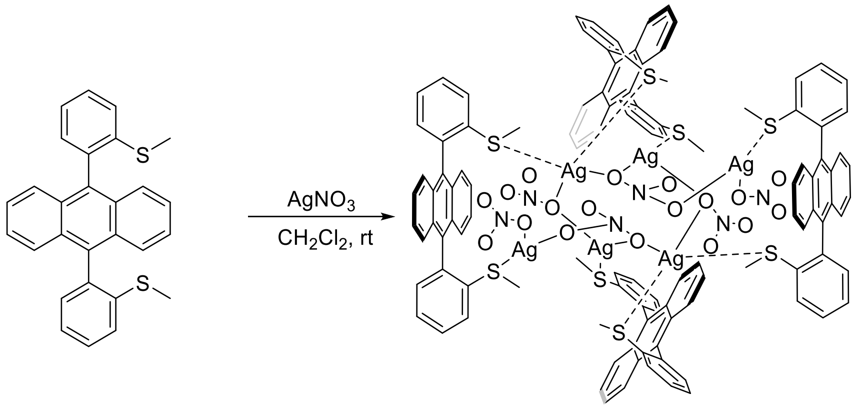


**1c**

To a solution of *syn*-**1** (6 mg, 0.014 mol) in dichloromethane (1 mL) was added silver nitrate (2.4 mg, 0.014 mmol) under argon atmosphere at room temperature. The mixture was stirred at ambient temperature for 4 h. Then the solution was concentrated to ca. 0.2 mL and diethyl ether (2 mL) was added to get a white precipitate. The solid was filtered, washed with diethyl ether and dried under reduced pressure to afford complex **1c** (5.1 mg, 80% yield).
M. p. 247.3 °C; FTIR (KBr): $\tilde{\nu}$ = 3428, 3052, 2919, 1620, 1473, 1434, 1384, 1300, 772, 754, 664 $cm^{-1}$
$^1$H NMR (300 MHz, $CDCl_3$) δ 7.63-7.69 (m, 8H, Benz-H), 7.47-7.63 (m, 40H, Anthr-H and Benz-H), 7.39-7.42 (m, 16H, Anthr-H), 2.37 (s, 24H, $SCH_3$).
$^{13}$C NMR (75 MHz, $CDCl_3$) δ 137.5, 135.3, 134.6, 131.7, 129.6 (d, *J* = 2.6 Hz), 127.4, 127.2, 126.7, 125.5, 18.4.

White needle monocrystals were grown by hexane diffusion into a solution of complex **1c** (2 mg) in $CH_2Cl_2$ (0.3 mL)

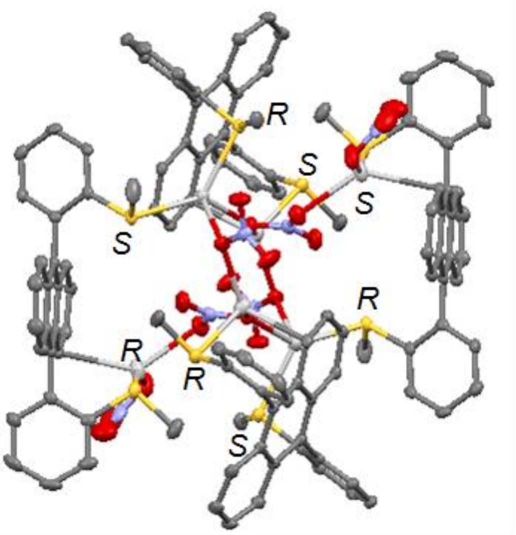

**Figure S2**. Solid-state structure of complex **1c** ($M_6L_4$), with *syn (R,S)-syn* **1** ligands.

Synthesis of complex **1d** = (**1**).($AgSO_2CF_3.PPh_3)_2$

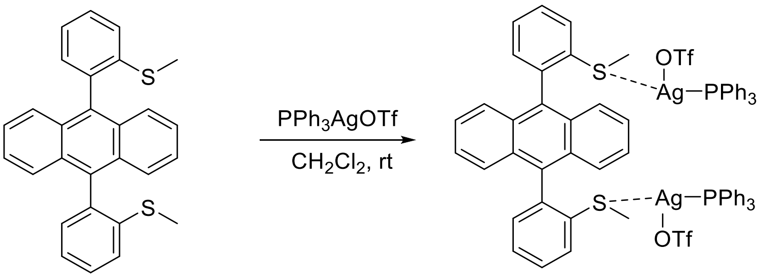


**1d**

To a solution of *syn*-**1** (6 mg, 0.014 mol) in dichloromethane (1 mL) was added triphenylphosphinesilver trifluoromethanesulfonate (7.4 mg, 0.014 mmol) under argon atmosphere at room temperature. The mixture was stirred at ambient temperature for 4 h. Then the solution was concentrated to *ca*. 0.2 mL and diethyl ether (2 mL) was added to get a white precipitate. The solid was filtered, washed with diethyl ether and dried under reduced pressure to afford complex **1d** as a light yellow powder (8 mg, 77% yield).
M. p. 125.8 °C; FTIR (KBr): $\tilde{\nu}$ = 3441, 3055, 2929, 1587, 1480, 1436, 1289, 1235, 1220, 1162, 1026, 779, 753, 694, 635, 516, 504 $cm^{-1}$;
$^{1}H$ NMR (300 MHz, $CDCl_3$) δ 7.64-7.69 (m, 4H, Benz-H), 7.51-7.61 (m, 6H, Anthr-H and Benz-H), 7.42-7.48 (m, 8H, Anthr-H and Benz-H and $PPh_3$), 7.29-7.39 (m, 28H, $PPh_3$), 2.34 (s, 6H, $SCH_3$)
$^{13}C$ NMR (75 MHz, $CDCl_3$) δ 137.4, 137.2, 135.4, 134.7, 133.9, 131.6, 131.1, 129.7, 129.3, 127.9, 127.1, 126.5, 125.6, 18.3
$^{19}F$ NMR (282 MHz, $CDCl_3$) δ -77.4 ppm
$^{31}P$ NMR (94 MHz, $CDCl_3$) δ 14.9 (bs) ppm.

Yellow monocystals were grown from hexane diffusion into a solution of complex **1d** (1 mg) in $CH_2Cl_2$ (0.2 mL)

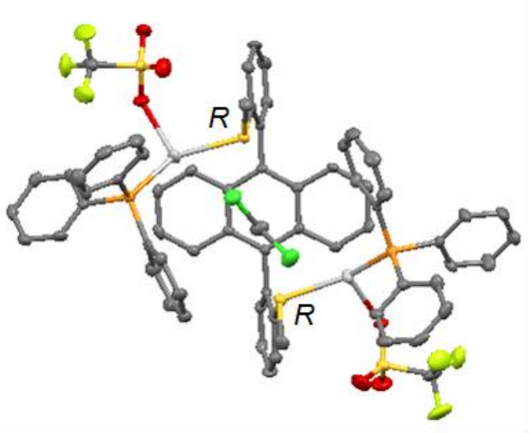


**Figure S3**. Solid-state structure of complex **1d** ($M_2L$), observed as a racemic mixture *(R,R) and (S,S)* complexes

**Table S1**. Selected lengths (Å) and angles (°) for X-ray structures of **1**, **1a** (two diastereoisomeric crystals), **1c** and **1d**.

| | **1** | **1a** (head-to-head) | **1a** (head-to-tail) | **1c** | **1d** |
|---|---|---|---|---|---|
| S···Ag$^+$ | - | Ag2-S4 2.509,<br>Ag2-S5 2.456 | Ag1-S1 2.478,<br>Ag1-S3 2.532,<br>Ag2-S4 2.563,<br>Ag2-S5 2.601 | Ag1-S1(S3) 2.570,<br>Ag2-S2 2.608,<br>Ag3-S4 2.502 | Ag1-S1 2.514,<br>Ag2-S3 2.521 |
| P···Ag$^+$ | - | - | - | - | Ag1-P1 2.396,<br>Ag2-P2 2.401 |
| <SAgS> | - | S1-Ag1-S2 147.0<br>S4-Ag2-S5 136.1 | S1-Ag1-S3 133.7<br>S4-Ag2-S5 148.8 | S1-Ag1-S3 131.5 | - |
| <PAgS> | - | - | - | - | P1-Ag1-S1 138.0,<br>P2-Ag2-S3 140.6 |
| Dihedral angles between the anthracene and its 9,10 aryl substituents | C20-C15-C1-C14 100.0<br>C28-C23-C8-C7 90.9<br>C58-C53-C38-C37 93.4<br>C50-C45-C31-C44 87.6 | C20-C15-C7-C6 98.4<br>C23-C24-C14-C13 83.5<br>C120-C115-C107-C106 104.5<br>C123-C128-C14-C113 100.5 | C20-C15-C13-C12 92.9<br>C27-C22-C6-C5 84.0<br>C55-C50-C32-C33 106.1<br>C48-C43-C39-C40 86.3 | C2-C7-C8-C9 81.6<br>C27-C22-C15-C16 95.0<br>C30-C35-C36-C49 70.8<br>C55-C50-C43-C42 82.6 | C16-C15-C13-C12 76.3<br>C26-C21-C6-C5 82.7 |

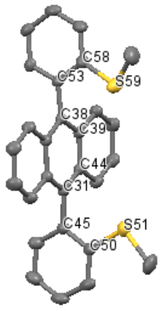

**1**

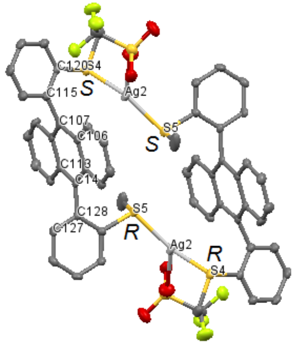

**1a** (Head-to-head)

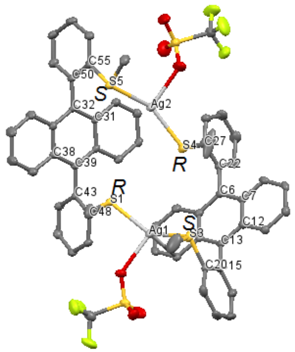

**1a** (Head-to-tail)

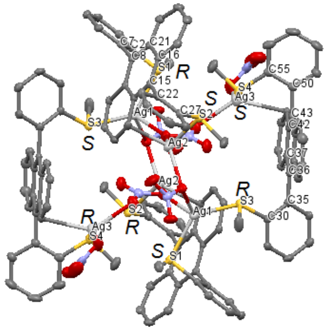

**1c**

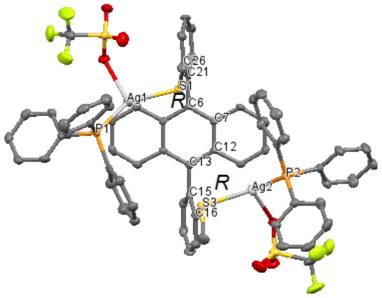

**1d**

**Table S2.** Crystal data and structure refinement for ligand *syn*-**1**

| | | |
|---|---|---|
| CCDC number | 1883674 | |
| Empirical formula | $C_{56} H_{44} S_4$ | |
| Formula weight | 845.15 | |
| Temperature | 153(2) K | |
| Wavelength | 1.54187 Å | |
| Crystal system | Monoclinic | |
| Space group | P21/c | |
| Unit cell dimensions | a = 20.742(3) Å | $\alpha$= 90° |
| | b = 8.9984(11) Å | $\beta$= 91.166(6) ° |
| | c = 23.764(4) Å | $\gamma$ = 90° |
| Volume | 4434.5(11) $Å^3$ | |
| Z | 4 | |
| Density (calculated) | 1.266 $Mg/m^3$ | |
| Absorption coefficient | 2.250 $mm^{-1}$ | |
| F(000) | 1776 | |
| Crystal size | 0.12 x 0.06 x 0.02 $mm^3$ | |
| Theta range for data collection | 6.40 to 68.25∞. | |
| Index ranges | -24≤ h ≤24, -10≤ k ≤10, -28≤ l ≤27 | |
| Reflections collected | 31983 | |
| Independent reflections | 8075 [R(int) = 0.0161] | |
| Completeness to theta = 68.25° | 99.5 % | |
| Absorption correction | Semi-empirical from equivalents | |
| Max. and min. transmission | 0.9564 and 0.7740 | |
| Refinement method | Full-matrix least-squares on $F^2$ | |
| Data / restraints / parameters | 8075 / 0 / 545 | |
| Goodness-of-fit on $F^2$ | 1.039 | |
| Final R indices [I>2sigma(I)] | R1 = 0.0385, wR2 = 0.1093 | |
| R indices (all data) | R1 = 0.0445, wR2 = 0.1134 | |
| Largest diff. peak and hole | 0.788 and -0.815 e.$Å^{-3}$ | |

**Table S3**. Crystal data and structure refinement for silver complex **1a** (Fig 1a, head-to-head coordination of ligands)

| | |
|---|---|
| CCDC number | 1883535 |
| Empirical formula | $C_{58} H_{44} Ag_2 F_6 O_6 S_6$ |
| Formula weight | 1359.03 |
| Temperature | 150(2) K |
| Wavelength | 0.71073 Å |
| Crystal system | Triclinic |
| Space group | P -1 |
| Unit cell dimensions | a = 13.4300(13) Å alpha = 117.822(2) ° |
| | b = 14.8251(14) Å beta = 103.344(2) ° |
| | c = 15.6390(13) Å gamma = 92.109(2) ° |
| Volume | 2641.4(4) $Å^3$ |
| Z | 2 |
| Calculated density | 1.709 $Mg/m^{-3}$ |
| Absorption coefficient | 1.053 $mm^{-1}$ |
| F(000) | 1368 |
| Crystal size | 0.120 x 0.100 x 0.030 $mm^3$ |
| Theta range for data collection | 1.534 to 26.381 ° |
| Limiting indices | -16≤ h ≤16, -18≤ k ≤18, -18≤ l ≤19 |
| Reflections collected / unique | 29382 / 10564 [R(int) = 0.0524] |
| Completeness to theta = 25.242 | 98.1 % |
| Absorption correction | Semi-empirical from equivalents |
| Refinement method | Full-matrix least-squares on F^2 |
| Data / restraints / parameters | 10564 / 6 / 760 |
| Goodness-of-fit on F^2 | 1.033 |
| Final R indices [I>2sigma(I)] | R1 = 0.0393, wR2 = 0.0887 |
| R indices (all data) | R1 = 0.0603, wR2 = 0.0993 |
| Extinction coefficient | n/a |
| Largest diff. peak and hole | 0.598 and -0.781 $e.Å^{-3}$ |

**Table S4.** Crystal data and structure refinement for silver complex **1a** (fig 1b, head-to-tail coordination of ligands)

| | |
|---|---|
| CCDC number | 1883532 |
| Empirical formula | $C_{58} H_{44} Ag_2 F_6 O_6 S_6$ |
| Formula weight | 1359.03 |
| Temperature | 120(2) K |
| Wavelength | 0.71073 Å |
| Crystal system | Monoclinic |
| Space group | P 21/c |
| Unit cell dimensions | a = 15.8914(11) Å alpha = 90 ° |
| | b = 14.2792(10) Å beta = 94.932(2) ° |
| | c = 26.5135(18) Å gamma = 90 ° |
| Volume | 5994.1 (7) $Å^3$ |
| Z | 4 |
| Calculated density | 1.506 $Mg/m^{-3}$ |
| Absorption coefficient | 0.928 $mm^{-1}$ |
| F(000) | 2736 |
| Crystal size | 0.600 x 0.150 x 0.060 $mm^3$ |
| Theta range for data collection | 1.542 to 26.760 ° |
| Limiting indices | -20≤ h ≤20, -18≤ k ≤18, -33≤ l ≤33 |
| Reflections collected / unique | 93239 / 12661 [R(int) = 0.0378] |
| Completeness to theta = 25.242 | 99.7 % |
| Absorption correction | Semi-empirical from equivalents |
| Max. and min. transmission | 0.7454 and 0.6047 |
| Refinement method | Full-matrix least-squares on F^2 |
| Data / restraints / parameters | 12661 / 1 / 707 |
| Goodness-of-fit on F^2 | 1.040 |
| Final R indices [I>2sigma(I)] | R1 = 0.0324, wR2 = 0.0744 |
| R indices (all data) | R1 = 0.0380, wR2 = 0.0761 |
| Extinction coefficient | n/a |
| Largest diff. peak and hole | 0.721 and -0.513 e.$Å^{-3}$ |

**Table S5.** Crystal data and structure refinement for silver complex **1c**

| | |
|---|---|
| CCDC number | 1883538 |
| Empirical formula | $C_{56}H_{44}Ag_3N_3O_9S_4$ |
| Formula weight | 1354.79 |
| Temperature | 150(2) K |
| Wavelength | 0.71073 Å |
| Crystal system | Triclinic |
| Space group | P -1 |
| Unit cell dimensions | a = 12.755(3) Å alpha = 111.882(5) ° |
| | b = 13.140(3) Å beta = 102.770(4) ° |
| | c = 16.721(4) Å gamma = 92.073(5) ° |
| Volume | 2514.4 (9) $Å^3$ |
| Z | 2 |
| Calculated density | 1.789 $Mg/m^{-3}$ |
| Absorption coefficient | 1.386 $mm^{-1}$ |
| F(000) | 1356 |
| Crystal size | 0.140 x 0.030 x 0.030 $mm^3$ |
| Theta range for data collection | 3.103 to 26.372 ° |
| Limiting indices | -15≤ h ≤15, -16≤ k ≤16, -20≤ l ≤20 |
| Reflections collected / unique | 78956 / 10193 [R(int) = 0.0603] |
| Completeness to theta = 25.242 | 99.2 % |
| Absorption correction | Semi-empirical from equivalents |
| Max. and min. transmission | 0.7454 and 0.6977 |
| Refinement method | Full-matrix least-squares on F^2 |
| Data / restraints / parameters | 10193 / 0 / 680 |
| Goodness-of-fit on F^2 | 1.023 |
| Final R indices [I>2sigma(I)] | R1 = 0.0319, wR2 = 0.0661 |
| R indices (all data) | R1 = 0.0494, wR2 = 0.0724 |
| Extinction coefficient | n/a |
| Largest diff. peak and hole | 0.971 and -0.739 $e.Å^{-3}$ |

**Table S6.** Crystal data and structure refinement for silver complex **1d**

| | |
|---|---|
| CCDC number | 1883536 |
| Empirical formula | $C_{67} H_{54} Ag_2 Cl_2 F_6 O_6 P_2 S_4$ |
| Formula weight | 1545.92 |
| Temperature | 150(2) K |
| Wavelength | 0.71073 Å |
| Crystal system | Orthorhombic |
| Space group | P n a 21 |
| Unit cell dimensions | a = 26.230(2) Å alpha = 90 ° |
| | b = 9.4521(8) Å beta = 90 ° |
| | c = 25.912(2) Å gamma = 90 ° |
| Volume | 6424.2 (9) Å$^3$ |
| Z | 4 |
| Calculated density | 1.598 Mg/m$^{-3}$ |
| Absorption coefficient | 0.942 mm$^{-1}$ |
| F(000) | 3120 |
| Crystal size | 0.150 x 0.040 x 0.020 mm$^3$ |
| Theta range for data collection | 1.553 to 26.374 ° |
| Limiting indices | -30≤ h ≤32, -11≤ k ≤11, -32≤ l ≤26 |
| Reflections collected / unique | 44069 / 11444 [R(int) = 0.0520] |
| Completeness to theta = 25.242 | 99.8 % |
| Absorption correction | Semi-empirical from equivalents |
| Max. and min. transmission | 0.7454 and 0.6655 |
| Refinement method | Full-matrix least-squares on F^2 |
| Data / restraints / parameters | 11444 / 1 / 805 |
| Goodness-of-fit on F^2 | 1.021 |
| Final R indices [I>2sigma(I)] | R1 = 0.0309, wR2 = 0.0547 |
| R indices (all data) | R1 = 0.0404, wR2 = 0.0578 |
| Extinction coefficient | n/a |
| Largest diff. peak and hole | 0.353 and -0.346 e.Å$^{-3}$ |

## Photophysical properties of ligand 1 and silver(I) complexes 1a–d

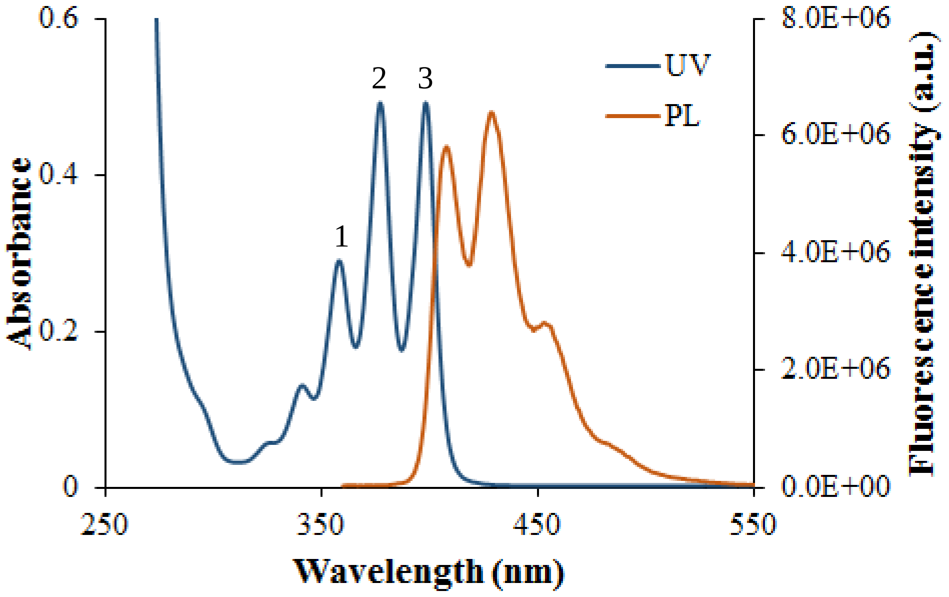


**Figure S4**. Absorption (blue) and fluorescence emission (red, $\lambda_{excitation}$ = 345 nm) spectra of ligand **1** (30 µM, $CH_2Cl_2$). Selected maxima of absorption: 0.29 (358 nm); 0.49 (377 nm); 0.49 (398 nm).

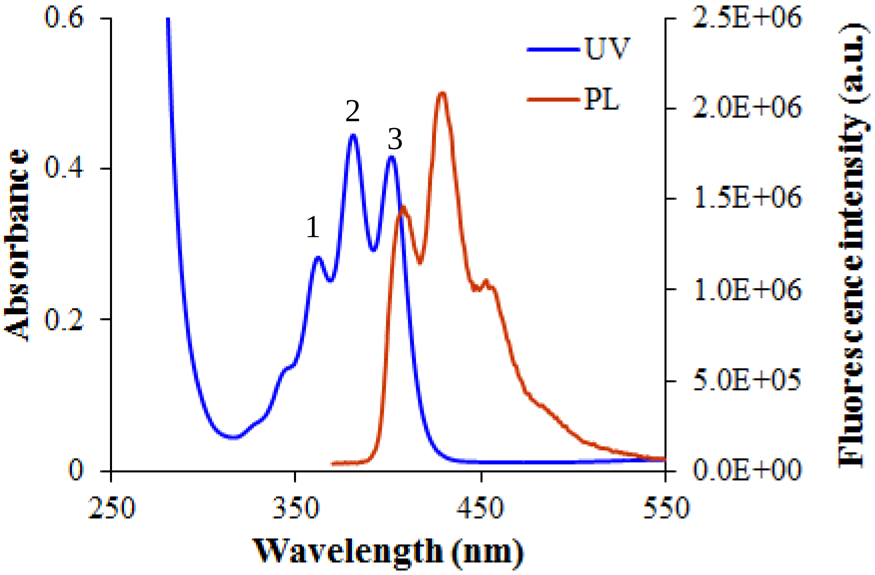


**Figure S5**. Absorption (blue) and fluorescence emission (red, $\lambda_{excitation}$ = 345 nm) spectra of complex **1a** (20 µM, $CH_2Cl_2$). Selected peaks of absorption: 0.28 (362 nm); 0.44 (381 nm); 0.41 (402 nm).

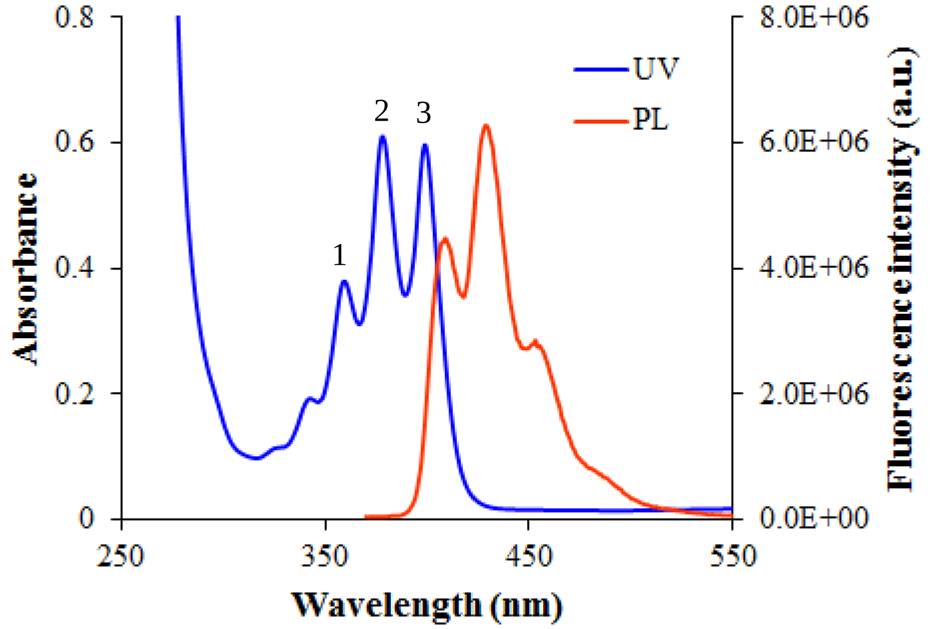


**Figure S6**. Absorption (blue) and fluorescence emission (red, $\lambda_{excitation}$ = 345 nm) spectra of complex **1b** (20 µM, $CH_2Cl_2$). Selected maxima of absorption: 0.38 (359 nm); 0.61 (378 nm); 0.60 (399 nm).

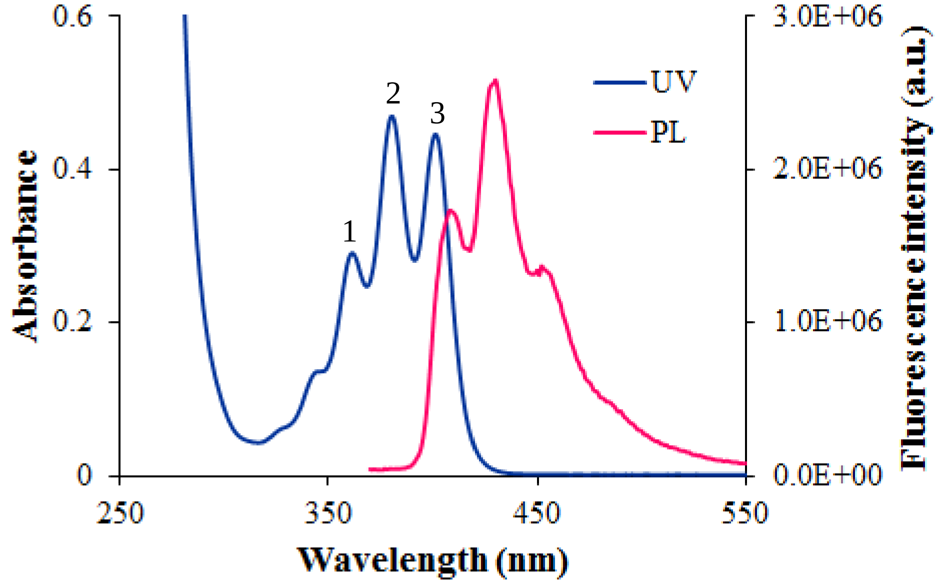


**Figure S7**. Absorption (blue) and fluorescence emission (pink, $\lambda_{excitation}$ = 345 nm) spectra of complex **1c** (20 µM, $CH_2Cl_2$). Selected maxima of absorption: 0.29 (361 nm); 0.47 (380 nm); 0.44 (401 nm).

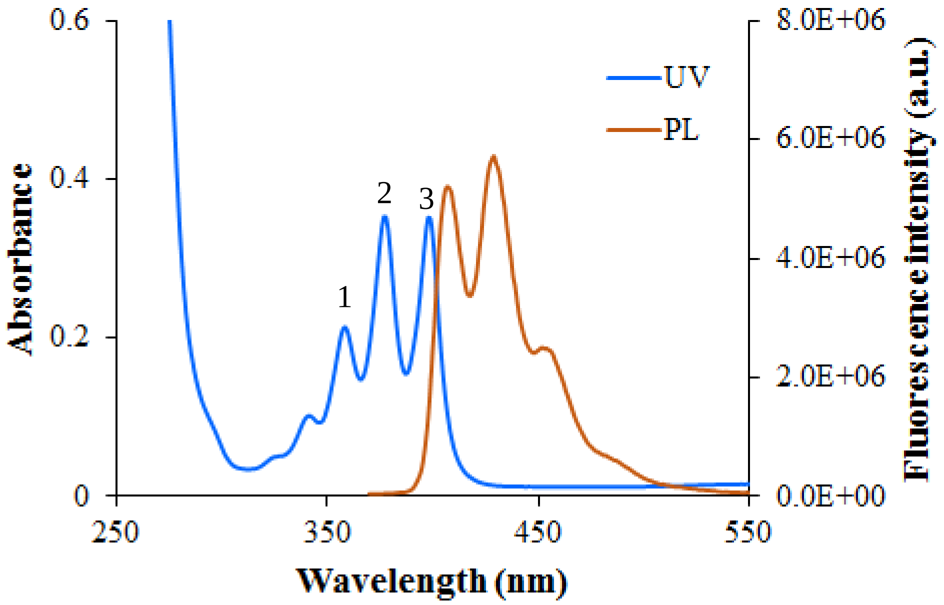


**Figure S8**. Absorption (blue) and fluorescence emission (red, $\lambda_{excitation}$ = 345 nm) spectra of complex **1d** (20 µM, $CH_2Cl_2$). Selected maxima of absorption: 0.21 (358 nm); 0.35 (377 nm); 0.35 (398 nm).

# $^1$H NMR Study

## $^1$H NMR of three batches of complex 1a

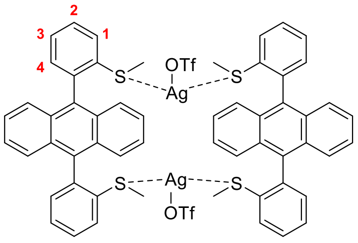


**1a**

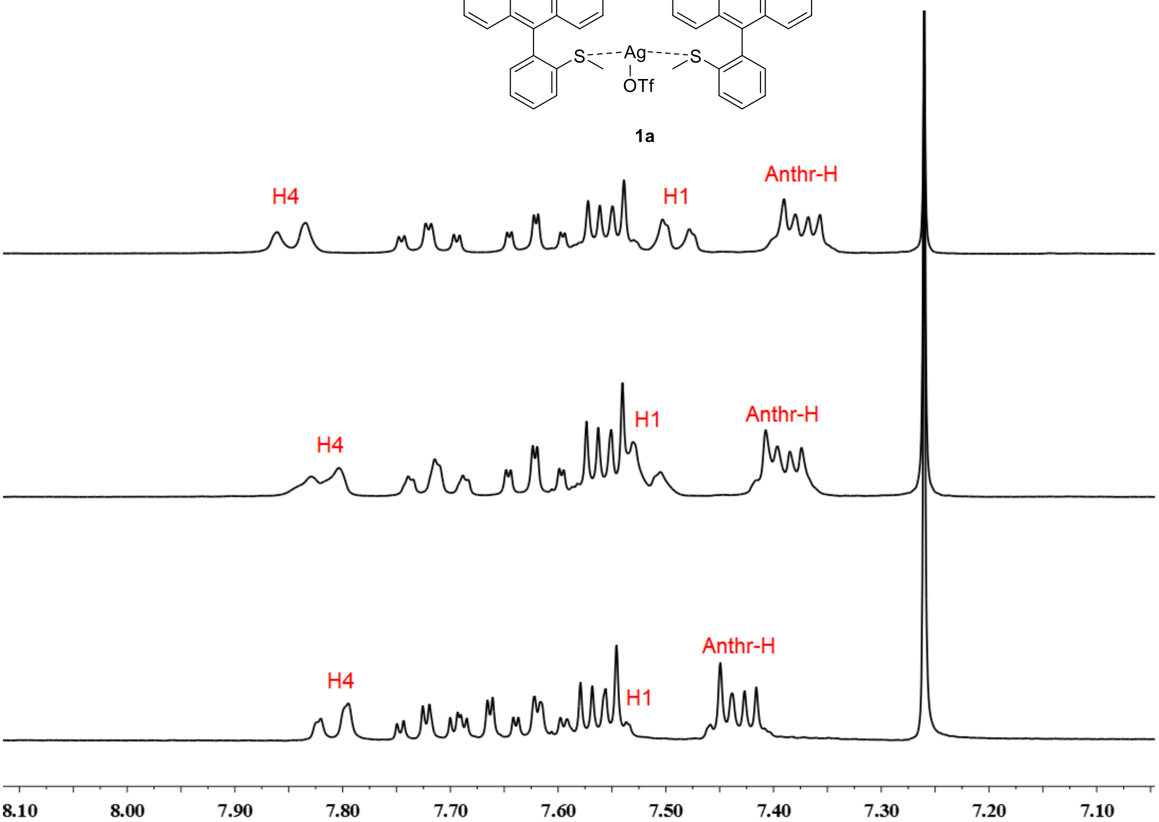


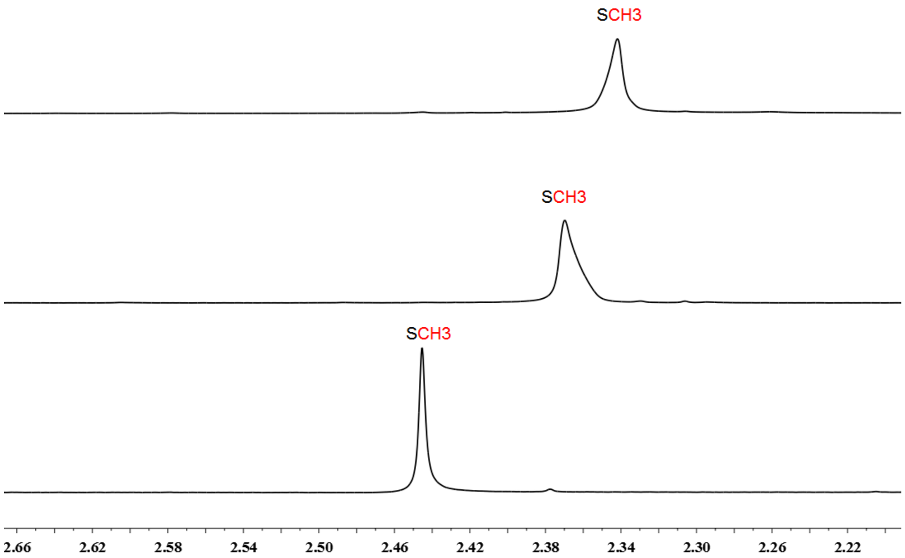


**Figure S9**. $^1$H NMR of three batches of complex **1a** (2 mM, $CDCl_3$, 298 K) obtained by the same procedure.

# Variable temperature NMR

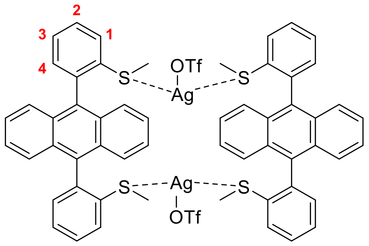


**1a**

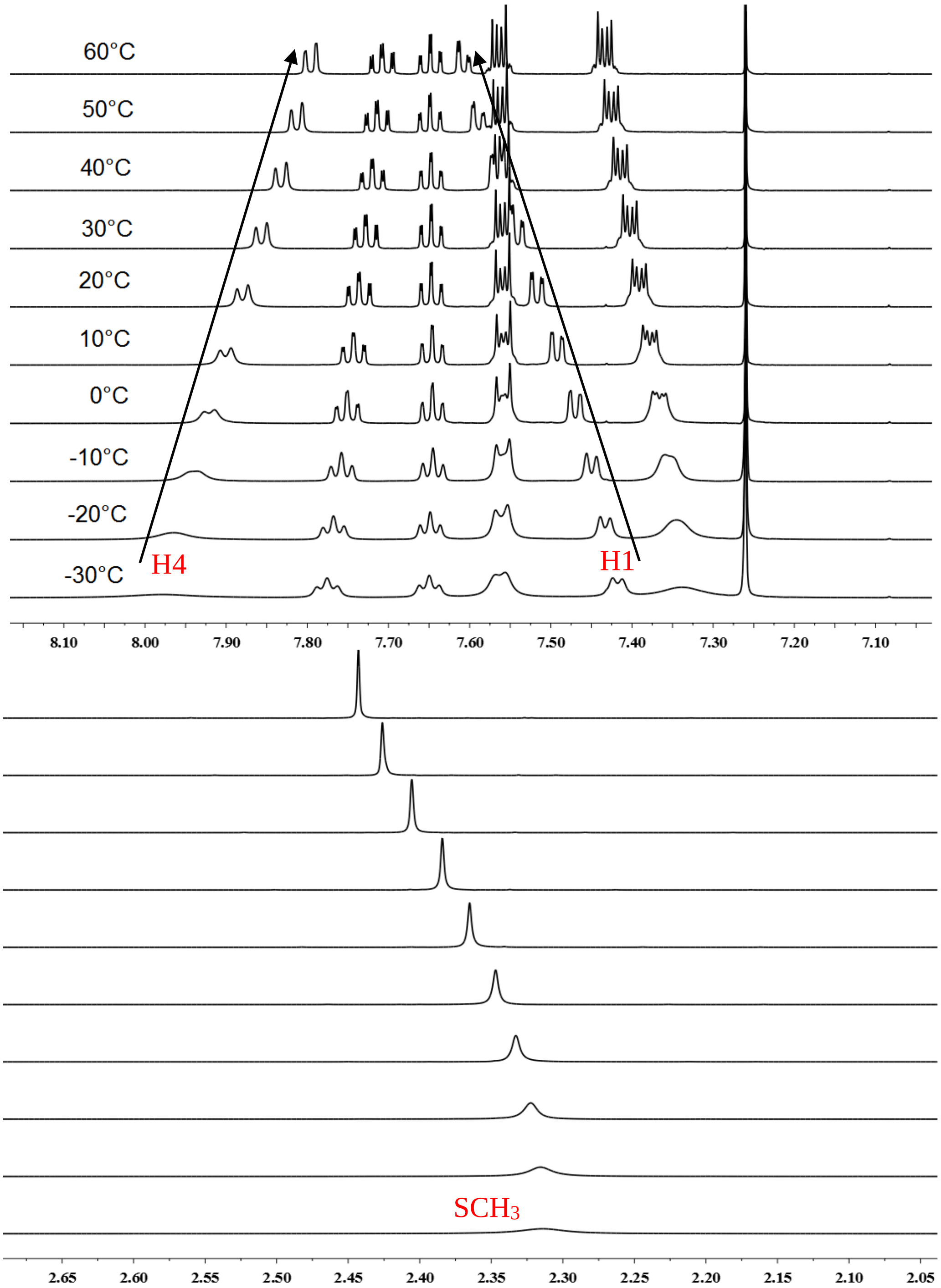


**Figure S10**. Proton VT-NMR of complex **1a** (7 mM, $CDCl_3$) from −30 °C to 60 °C.

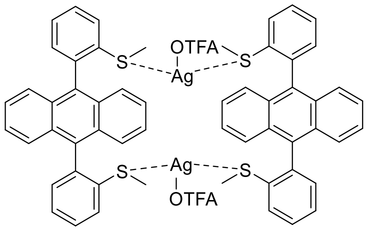


**1b**

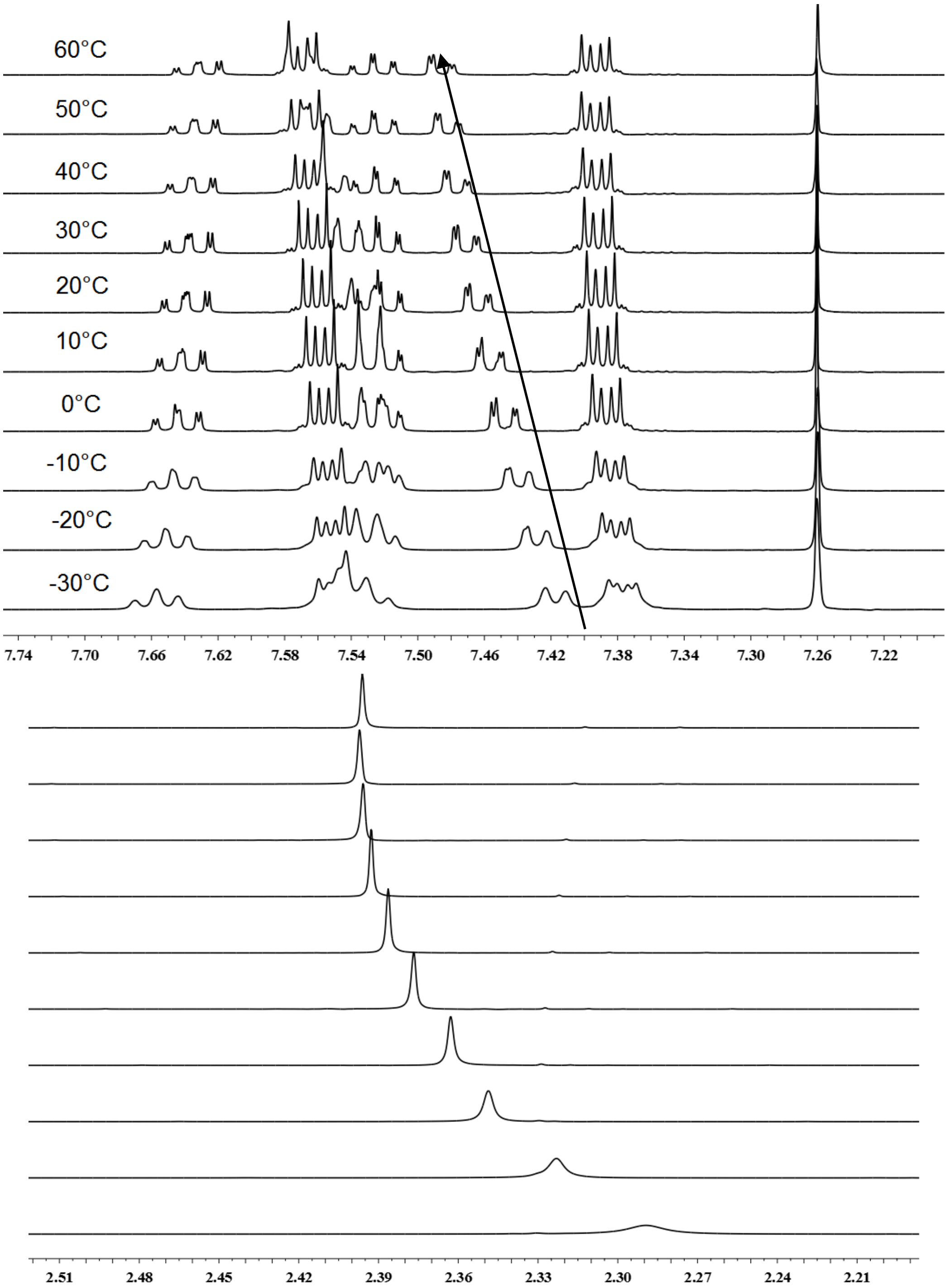


**Figure S11**. Variable temperature $^{1}$H NMR of complex **1b** (7 mM, $CDCl_3$) from −30 °C to 60 °C.

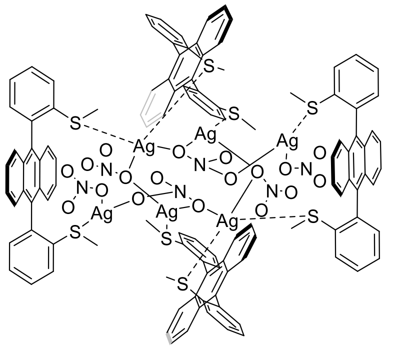


**1c**

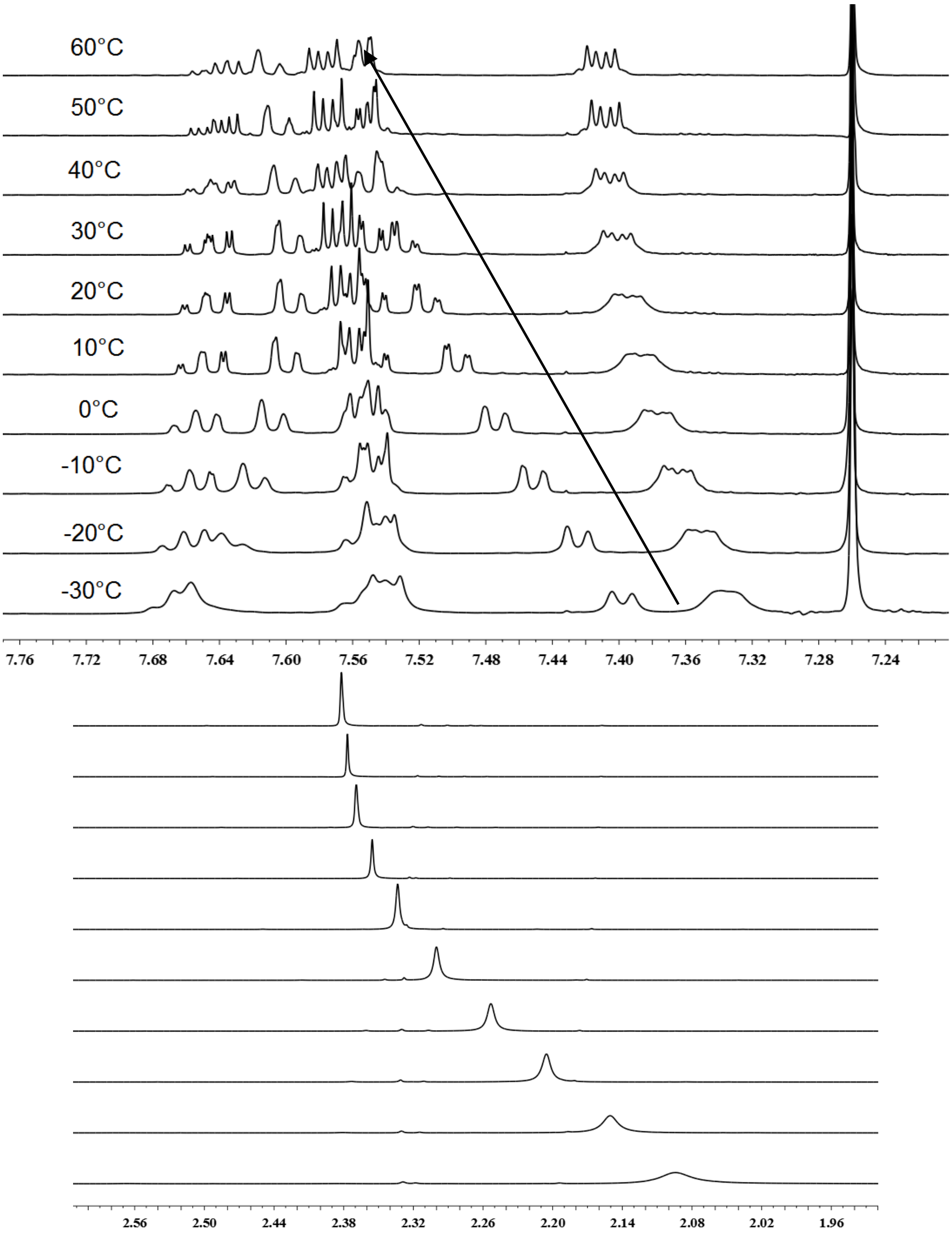


**Figure S12**. Variable temperature $^{1}$H NMR of complex **1c** (7 mM, $CDCl_3$) from −30 °C to 60 °C.

## Diffusion-Ordered Spectroscopy $^1$H NMR (DOSY)

**General Information:**

NMR experiments were performed at 298 K using a Bruker Avance III 600 ($^1$H: 600.16 MHz, $^{13}$C: 150.93 MHz) instrument equipped with a 5 mm gradient inverse broadband probe.

DOSY NMR spectroscopy: “Two-dimensional” version of the pulsed field gradient spinecho experiment for measuring translational diffusion constants using the following parameters: spectral width, 10 ppm; number of scans 8; recycling delay 1 s, intergradient delay Δ 150 ms, gradient pulse duration δ 1 ms. The pulse gradients (*G*) were incremented from 2 to 95% of the maximum gradient strength in a linear ramp in 16 steps. The diffusion coefficient *D* can be obtained by fitting a specific resonance area, I, obtained at different gradient powers (G) using the following equation: $I/I0 = \exp[-\gamma GD\ \delta\ (\Delta - \delta/3)]$

**Table S7**. Diffusion coefficients (*D*, × $10^{-9}$ $m^2/s$) of silver complexes **1a–d** (600 MHz, $CDCl_3$, 5–7 mM, 298 K)

| D[a] | First series<br>Batch 1<br>(conc.) | Second series<br>Batch 2 (conc.) | Third series<br>Batch 3 (conc.) |
|---|---|---|---|
| **1a** | 1.85 (6.1 mM) | 0.73 (5 mM)[b]; 0.58 (5 mM)[b] | 1.69 (5 mM)[c]; 2.00 (5 mM)[c] |
| **1b** | 1.76 (6.5 mM) | 0.80 (5 mM)[b]; 0.89 (5 mM)[b] | 1.69 (5 mM)[c]; 1.35 (5 mM)[c] |
| **1c** | 0.93 (7 mM) | 0.76 (5 mM)[b]; 0.81 (5 mM)[b] | - |
| **1d** | 0.91 (5.7 mM) | 0.85 (5 mM)[b]; 0.80 (5 mM)[b] | - |

[a]Average D values calculated for aromatic and aliphatic protons, with estimated errors of ± 15–20%. [b]Measurements were done twice on the same NMR tube. [c]Measurements were run on the same batch of complex but done in different NMR tube.

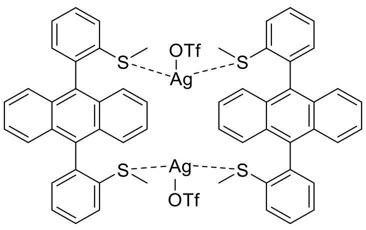


**1a**

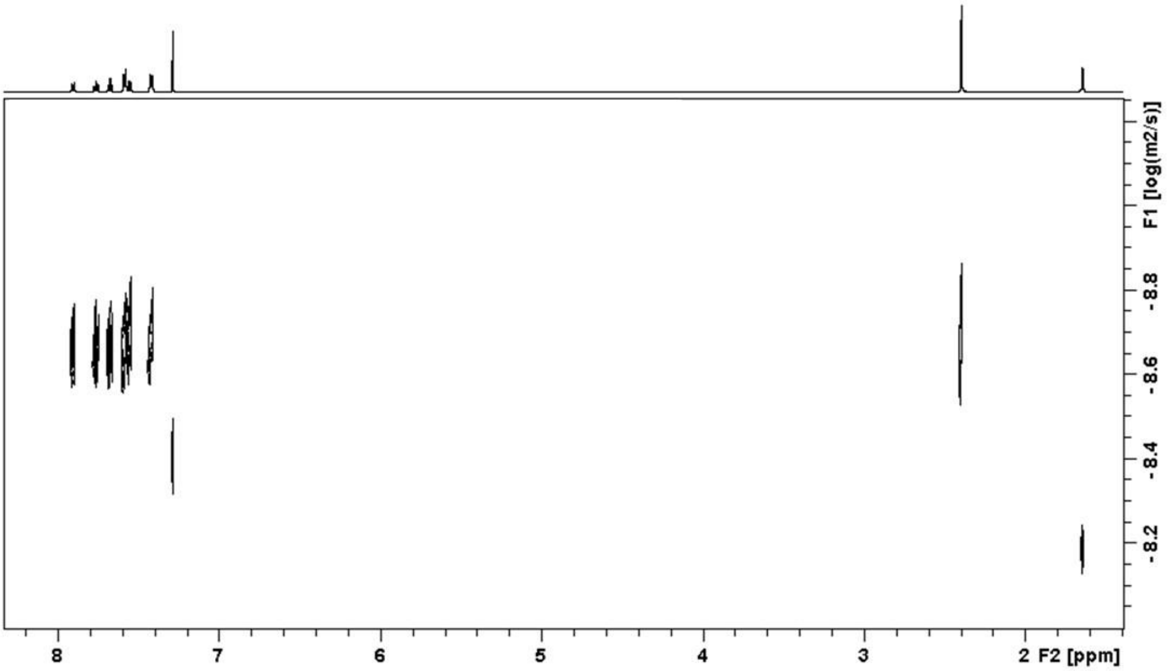


**Figure S13**. DOSY spectrum of complex **1a** (5 mM) in $CDCl_3$ .

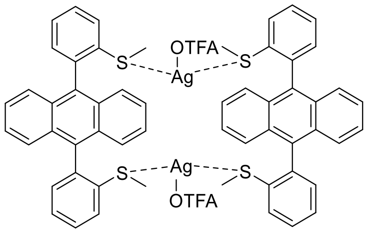


**1b**

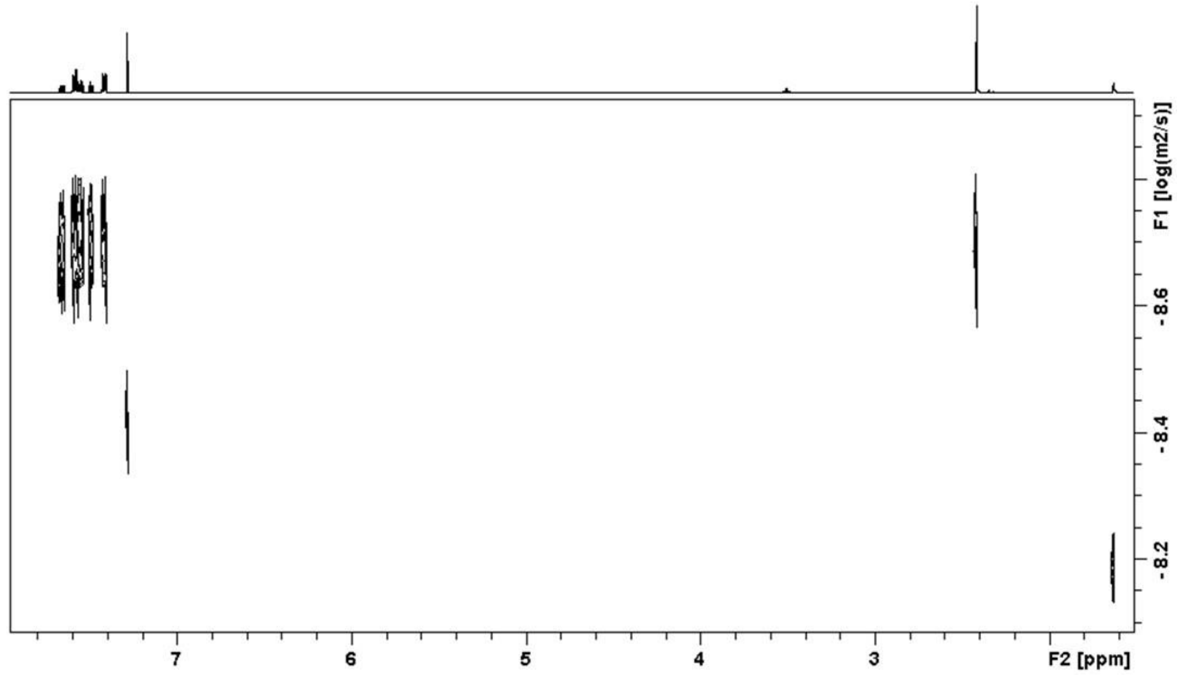


**Figure S14**. DOSY spectrum of complex **1b** (5 mM) in $CDCl_3$.

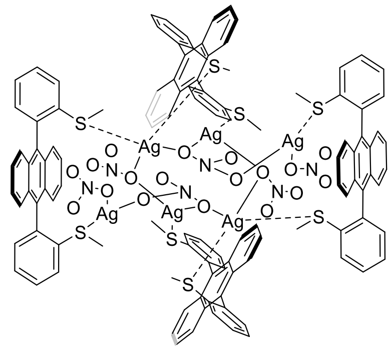


**1c**

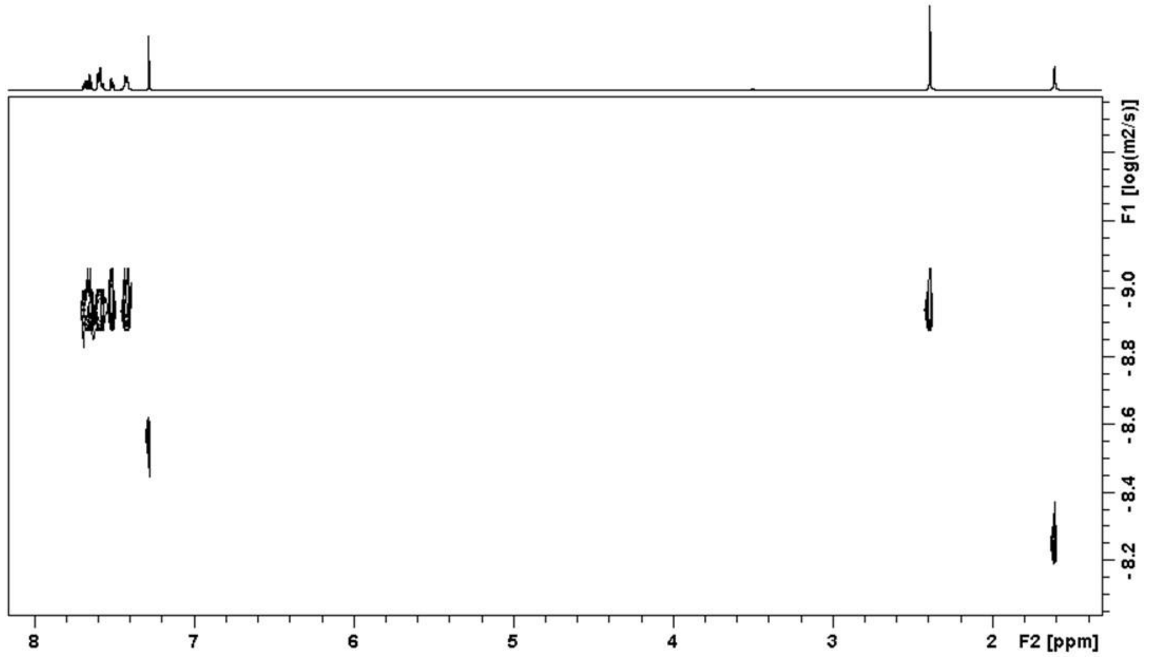


**Figure S15**. DOSY spectrum of complex **1c** (5 mM) in $CDCl_3$

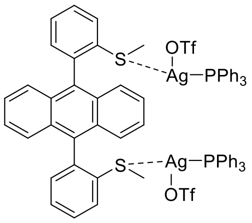


**1d**

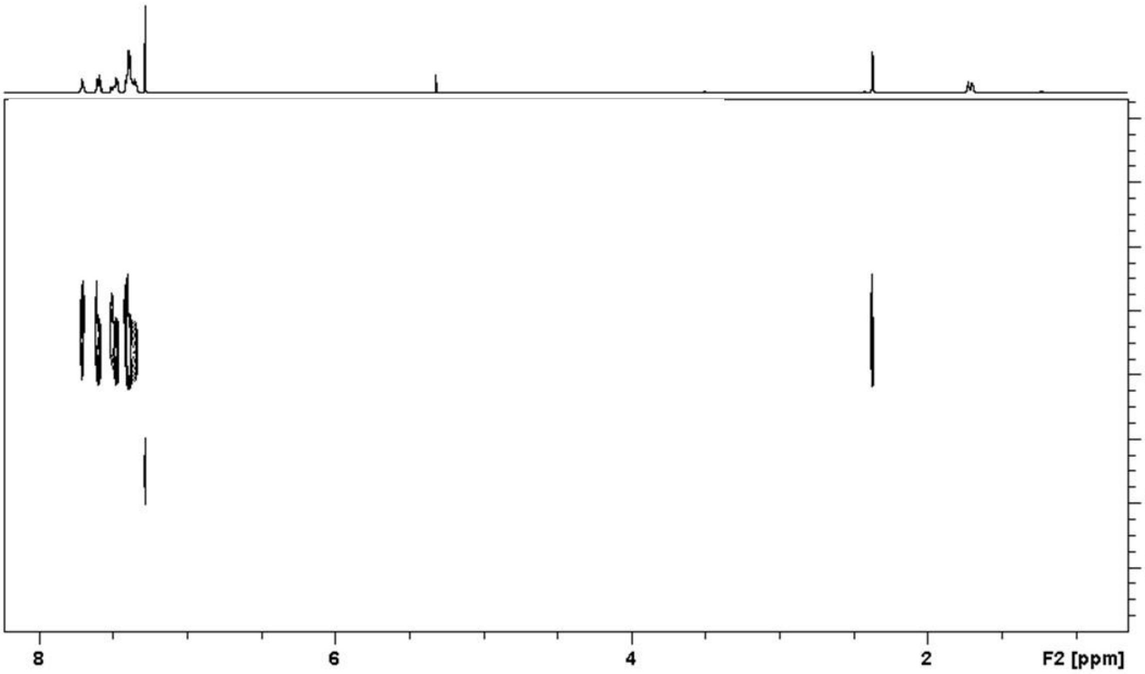


**Figure S16**. DOSY spectrum of complex **1d** (5 mM) in $CDCl_3$

## Silver catalysis

## Synthesis of alkynes for silver catalysed cyclization

Synthesis of alkyne **2**[5]

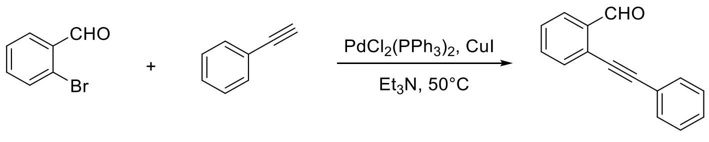


**2**

Compound **2**: An oven-dried Schlenk tube was charged with 2-bromobenzaldehyde (0.31 mL, 2.7 mmol), phenylacetylene (0.31 mL, 2.8 mmol), copper iodide (10.5 mg, 0.054 mmol), and bis(triphenylphosphine)palladium chloride (38 mg, 0.054 mmol). The mixture was degassed and flushed with argon for three times, then triethylamine (5 mL) was added and the resulting mixture was stirred at 50 °C for 24 h. After cooling to room temperature, the slurry was taken up in dichloromethane (50 mL) and water (20 mL). The aqueous layer was extracted with $CH_2Cl_2$ (2 × 20 mL). The combined organic layers were dried over magnesium sulfate, then concentrated and purified by column chromatography on silica gel (eluent: petrol ether/ethyl acetate = 75:1) to afford product **2** (438 mg, 79% yield) as reddish oil.
$^1$H NMR (300 MHz, $CDCl_3$) δ 10.65 (s, 1H), 7.95 (dd, *J* = 7.59, 1.02 Hz, 1H), 7.54-7.66 (m, 4H), 7.36-7.48 (m, 4H); $^{13}$C NMR (75 MHz, $CDCl_3$) δ 191.7, 135.9, 133.8, 133.3, 131.7, 129.1, 128.7, 128.6, 127.3, 126.9, 122.4, 96.4, 85.0. The data are identical to literature.[5]

Synthesis of alkyne **3**[6]

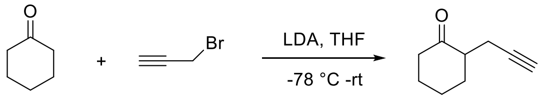


**3**

Compound **3**: To a solution of LDA (6.2 mL, 14.4 mmol, 2 M) in anhydrous THF (3 mL) was added dropwise cyclohexanone (1.0 mL, 9.6 mmol) at -78 °C under argon atmosphere. The mixture was stirred at −78 °C for 30 min then propargyl bromide (1.0 mL, 11.5 mmol) was added. The resulting mixture was stirred at −78 °C for further 1 h and then allowed to warm up to room temperature and stirred overnight. Saturated ammonium chloride (5 mL) was added and the mixture was extracted with dichloromethane (3 × 15 mL). The combined organic layers were dried over magnesium sulfate, then concentrated and purified by column chromatography on silica gel (eluent: petrol ether/ ethyl acetate = 75:1) to afford product **3** (827 mg, 63% yield, estimated purity *ca*. >90%) as a light yellow oil.
$^1$H NMR (300 MHz, $CDCl_3$) δ 2.00-2.64 (m, 8H), 1.95 (t, *J* = 2.7 Hz, 1H), 1.81-1.93 (m, 1H), 1.55-1.79 (m, 2H), 1.33-1.47 (m, 1H); $^{13}$C NMR (75 MHz, $CDCl_3$) δ 210.9, 82.6, 69.5, 49.6, 42.0, 33.3, 27.9, 25.2, 18.9.
The data are identical to the literature.[6]

**General procedure for intramolecular cyclization of alkyne 2 to compound 4**

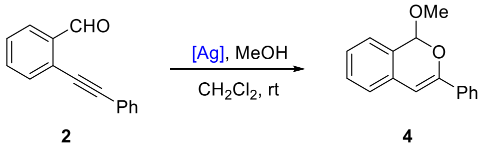


A dried Schlenk tube was charged with the silver salt (0.5 mmol%-5 mol %), then degassed and backfilled with argon for three times. A solution of alkyne **2** (30 mg, 0.15 mmol) in anhydrous $CH_2Cl_2$ (1 mL) and MeOH (18 µL, 0.45 mmol) was added and the mixture was stirred at room temperature for 12 h. The crude solution was concentrated under reduced pressure and purified by column chromatography on silica gel (eluent: petrol ether/ ethyl acetate = 50:1) to obtain product **4**.
$^1$H NMR (300 MHz, $CDCl_3$) δ 7.81-7.85 (m, 2H), 7.33-7.45 (m, 4H), 7.26-7.31 (m, 2H), 7.23 (d, $J$ = 7.44 Hz, 1H), 6.62 (s, 1H), 6.16 (s, 1H), 3.62 (s, 3H); $^{13}$C NMR (75 MHz, $CDCl_3$) δ 149.5, 134.5, 130.0, 129.6, 128.8, 128.5, 127.1, 126.8, 125.9, 124.9, 124.6, 100.5, 99.9, 55.3. The data are identical to the literature.[7]

**General procedure for intermolecular cyclization of alkyne 3 to compound 5**

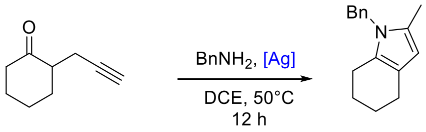


A dried Schlenk tube was charged with silver catalysis (0.5 mmol %-2.5 mol %), then degassed and backfilled with argon for three times. A solution of alkyne **3** (27 mg, 0.2 mmol) in anhydrous 1,2-dichloroethane (1 mL) and benzylamine (33 µL, 0.3 mmol) was added. The mixture was stirred at 50 °C for 12 h. The crude solution was concentrated under reduced pressure and purified by column chromatography on silica gel (eluent: petrol ether/ ethyl acetate = 70:1) to obtain product **5**.
$^1$H NMR (300 MHz, $CDCl_3$) δ 7.21-7.34 (m, 3H), 6.94 (d, $J$ = 7.14 Hz, 2H), 5.76 (s, 1H), 4.96 (s, 2H), 2.52 (t, $J$ = 5.94 Hz, 2H), 2.43 (t, $J$ = 5.43 Hz, 2H), 2.15 (s, 3H), 1.70-1.84 (m, 4H); $^{13}$C NMR (75 MHz, $CDCl_3$) δ 138.9, 128.7, 127.2,127.1, 127.0, 125.9, 116.4, 105.2, 46.2, 23.9, 23.6, 23.2, 22.2, 12.1. The data are identical to the literature.[6]

## Infrared spectroscopy

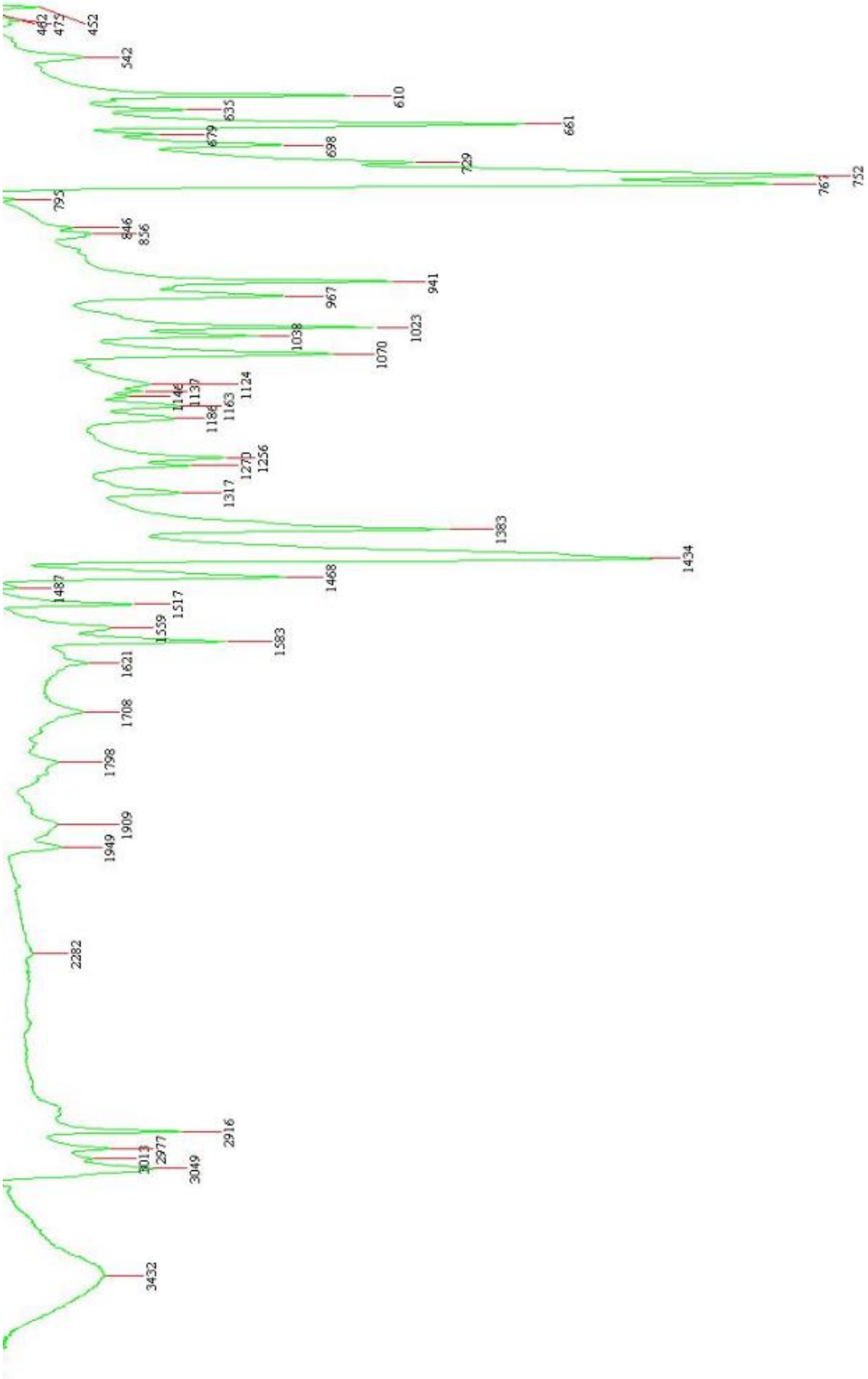


**Figure 17**. Infrared spectroscopy of ligand **1**.

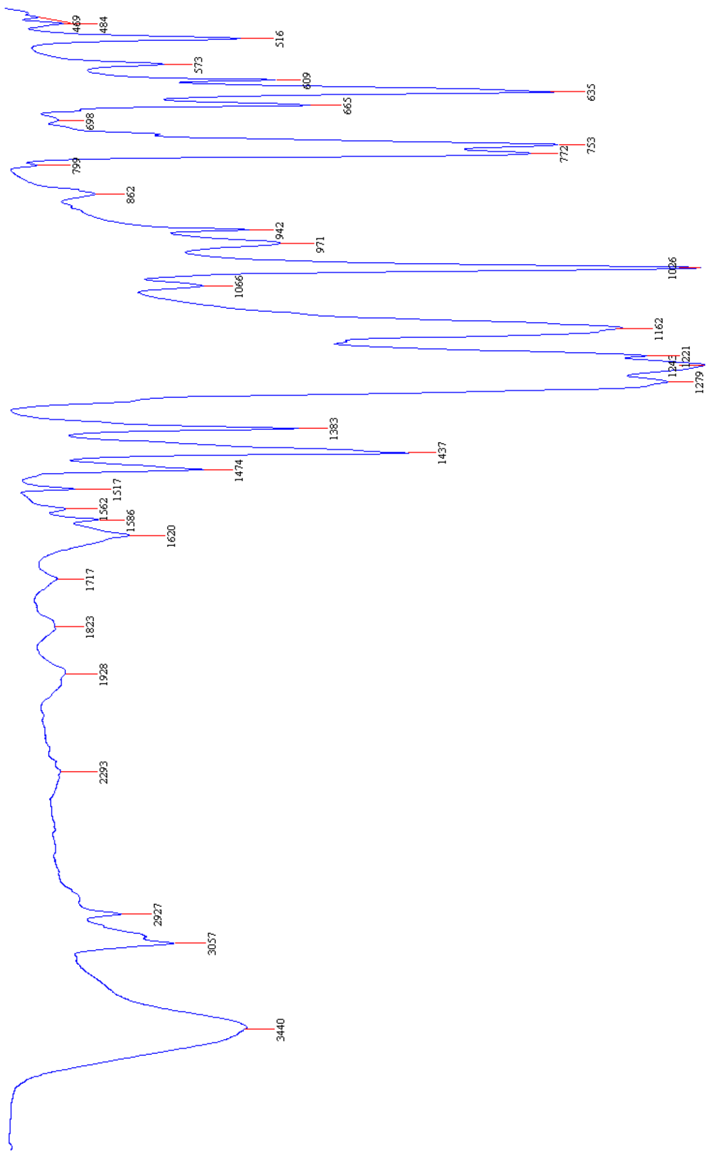


**Figure 18**. Infrared spectroscopy of complex **1a**.

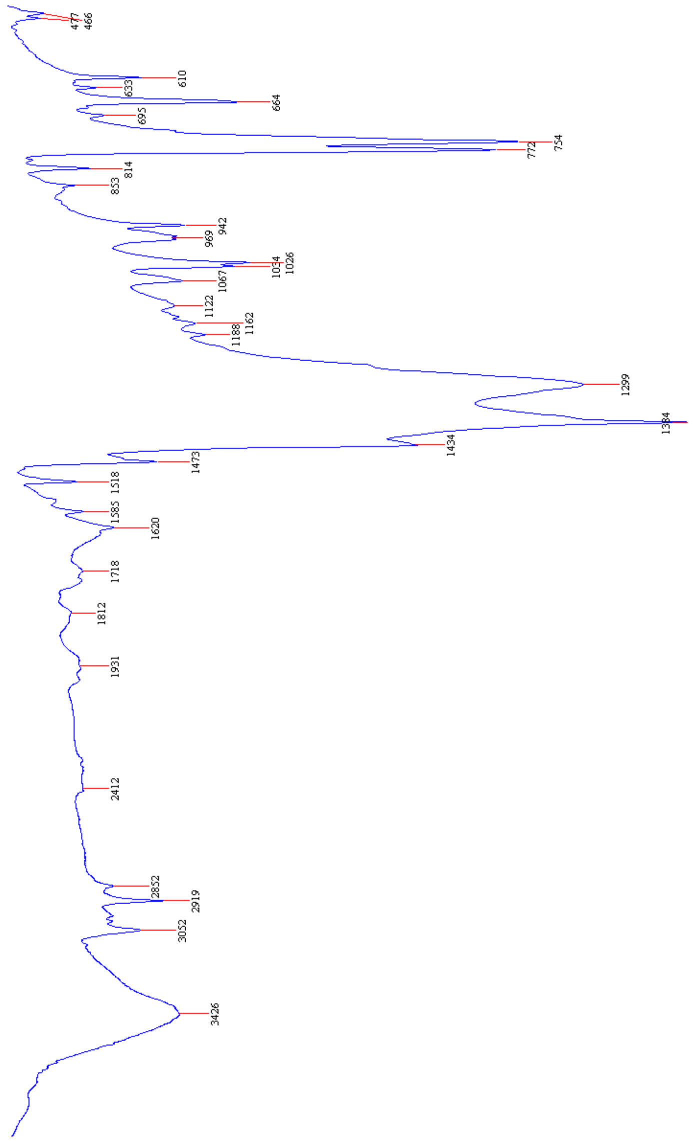


**Figure 19**. Infrared spectroscopy of complex **1b**.

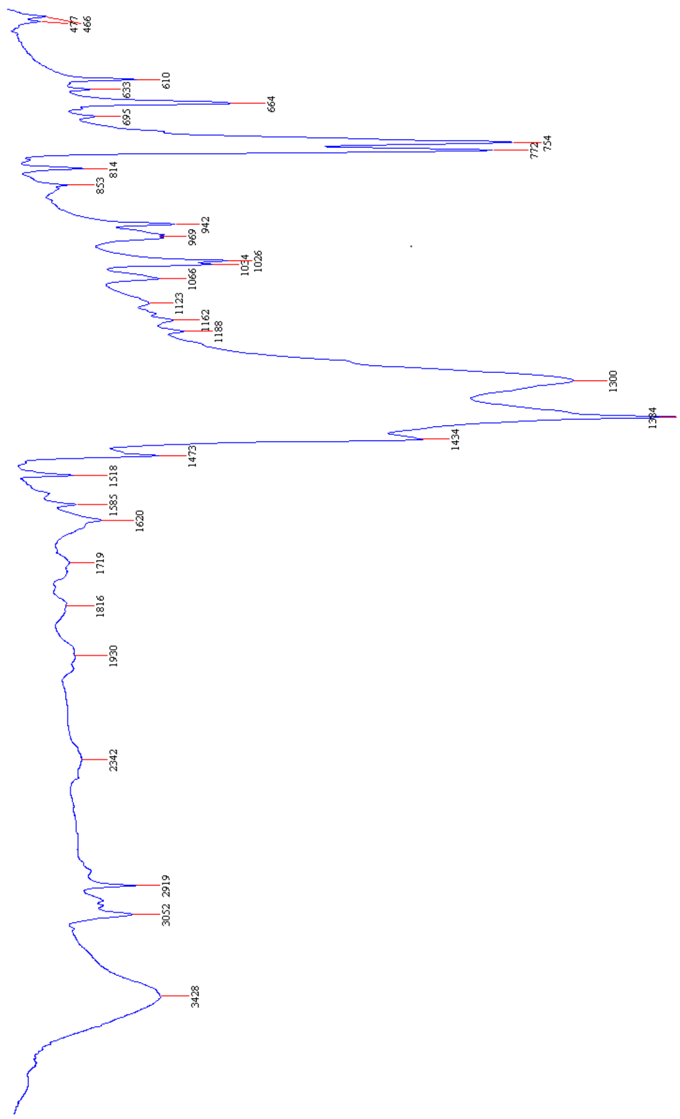


**Figure 20**. Infrared spectroscopy of complex **1c**.

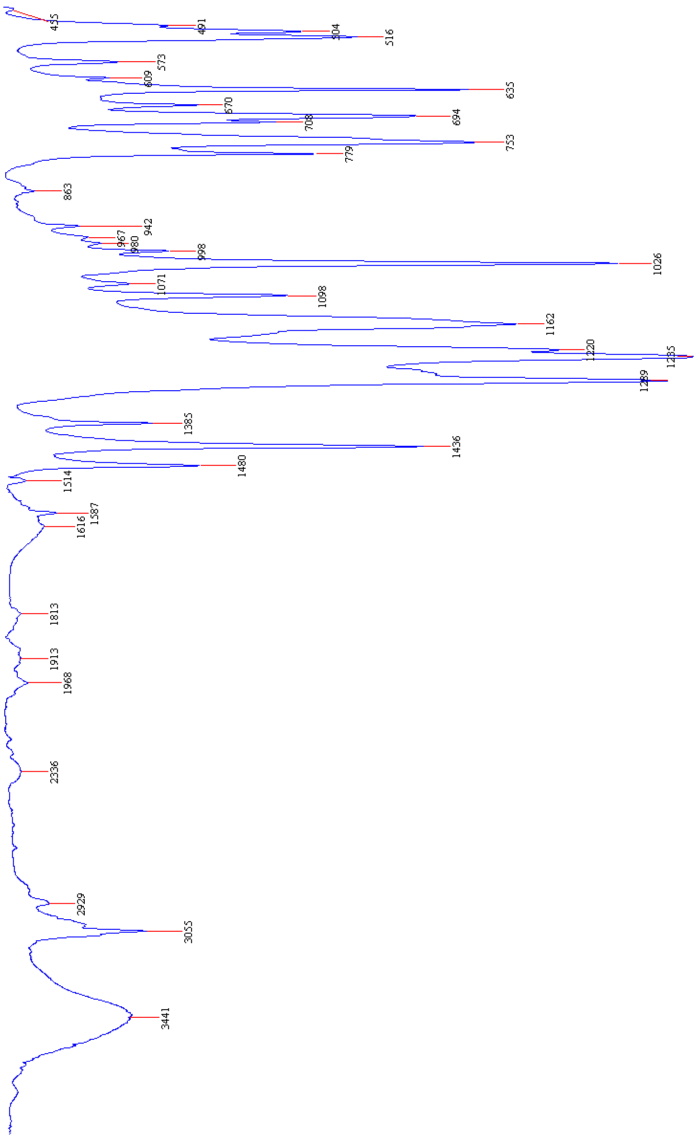


**Figure 21**. Infrared spectroscopy of complex **1d**.

# NMR spectra

Ligand **1**

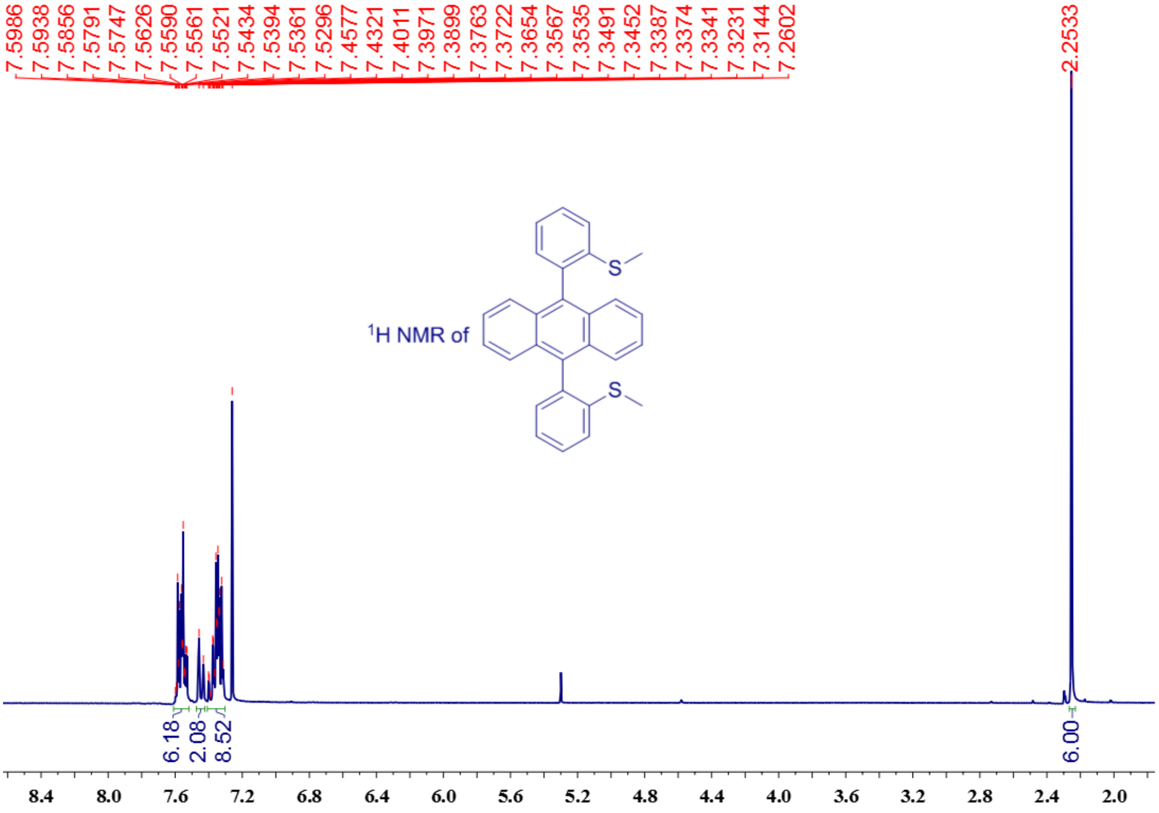


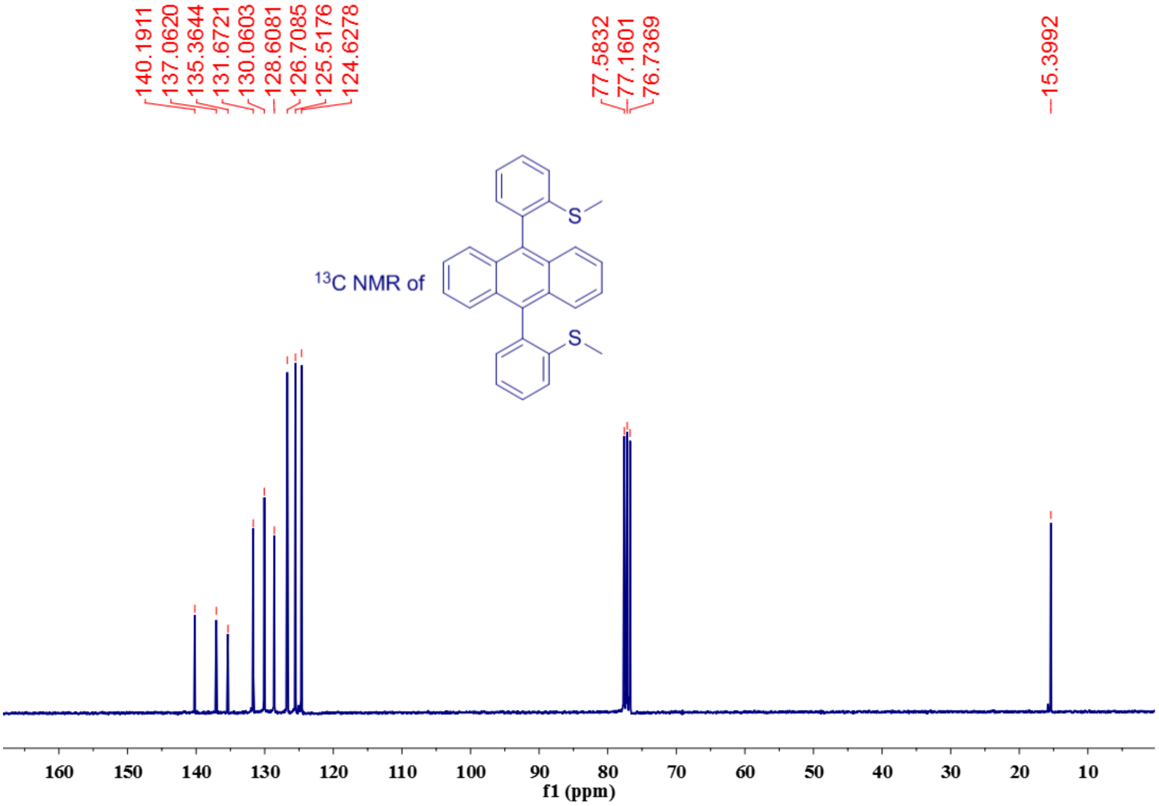

Complex **1a**

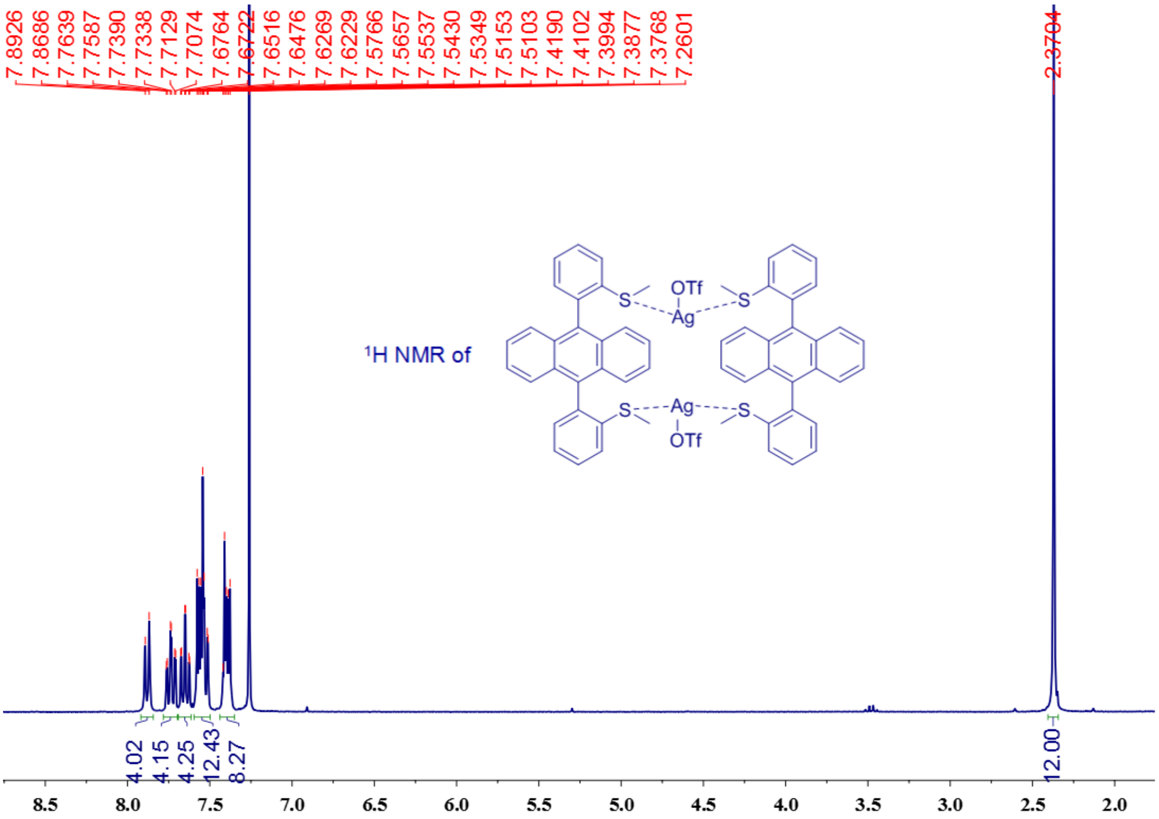

7.8926
7.8686
7.7639
7.7587
7.7390
7.7338
7.7129
7.7074
7.6764
7.6722
7.6516
7.6476
7.6269
7.6229
7.5766
7.5657
7.5537
7.5430
7.5349
7.5153
7.5103
7.4190
7.4102
7.3994
7.3877
7.3768
7.2601
2.3704
1H NMR of
OTf
Ag
S
4.02
4.15
4.25
12.43
8.27
12.00
8.5
8.0
7.5
7.0
6.5
6.0
5.5
5.0
4.5
4.0
3.5
3.0
2.5
2.0


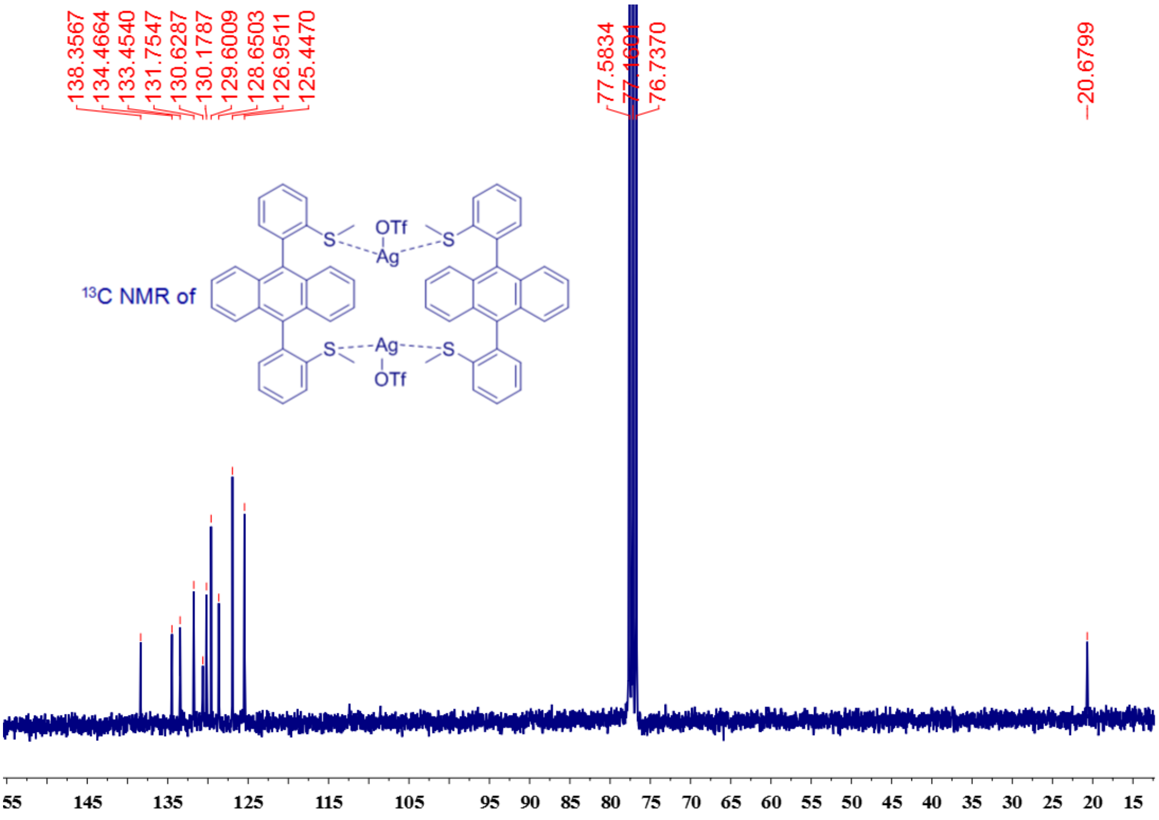

138.3567
134.4664
133.4540
131.7547
130.6287
130.1787
129.6009
128.6503
126.9511
125.4470
77.5834
76.7370
20.6799
13C NMR of
OTf
Ag
S
55
145
135
125
115
105
95
90
85
80
75
70
65
60
55
50
45
40
35
30
25
20
15

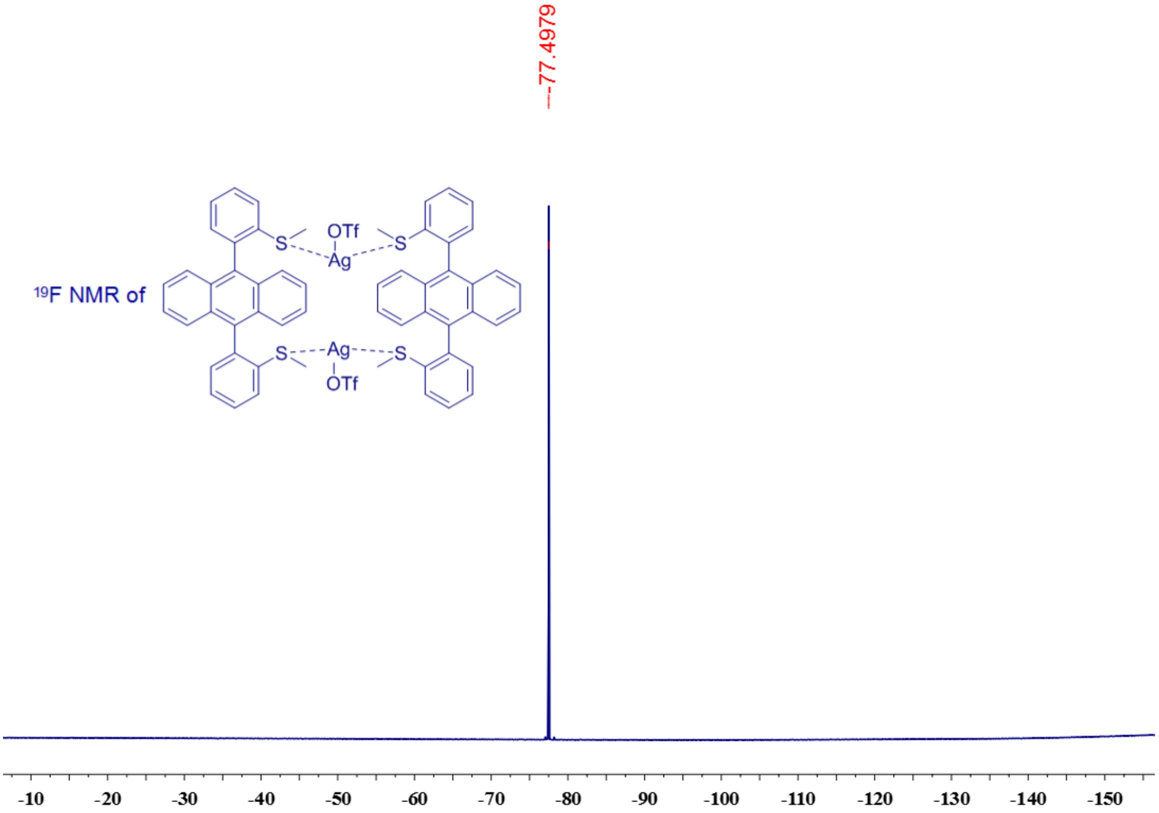

-77.4979
19F NMR of
OTf
Ag
S
-10
-20
-30
-40
-50
-60
-70
-80
-90
-100
-110
-120
-130
-140
-150


Complex **1b**

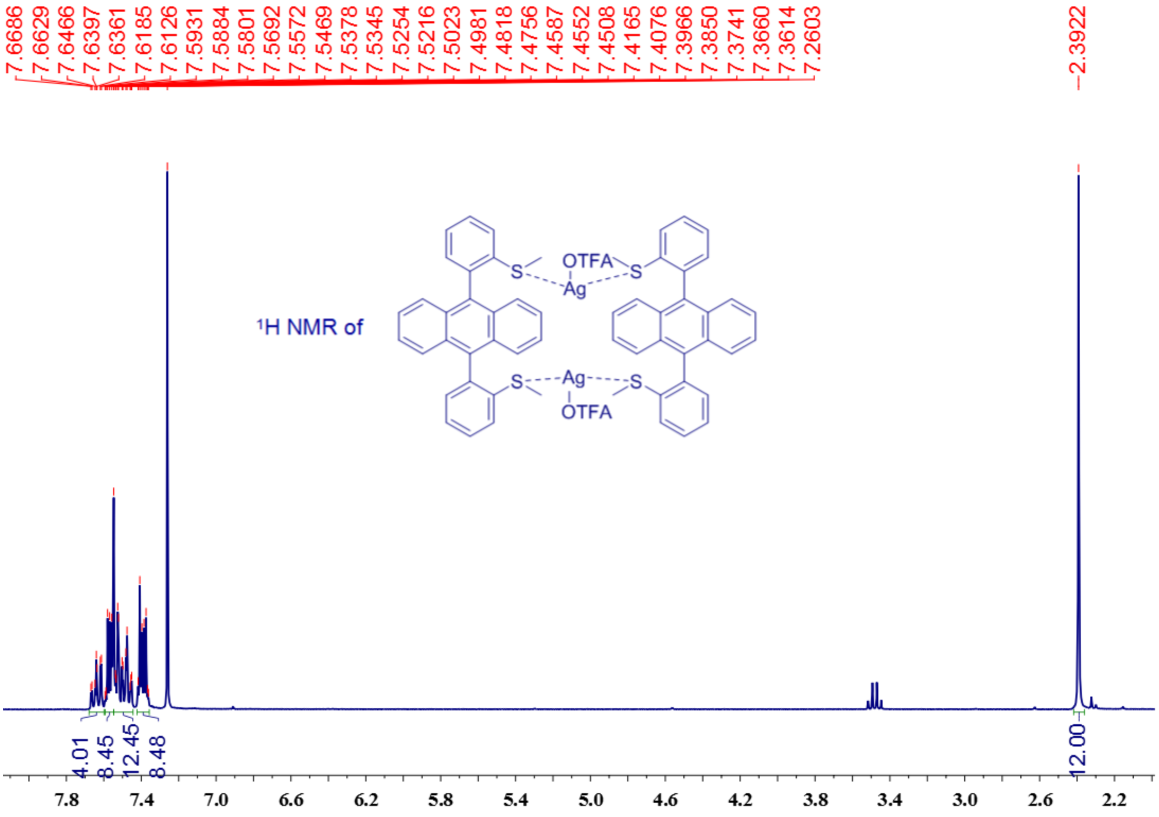

7.6686
7.6629
7.6466
7.6397
7.6361
7.6185
7.6126
7.5931
7.5884
7.5801
7.5692
7.5572
7.5469
7.5378
7.5345
7.5254
7.5216
7.5023
7.4981
7.4818
7.4756
7.4587
7.4552
7.4508
7.4165
7.4076
7.3966
7.3850
7.3741
7.3660
7.3614
7.2603
2.3922
1H NMR of
OTFA
Ag
S
4.01
8.45
12.45
8.48
12.00
7.8
7.4
7.0
6.6
6.2
5.8
5.4
5.0
4.6
4.2
3.8
3.4
3.0
2.6
2.2

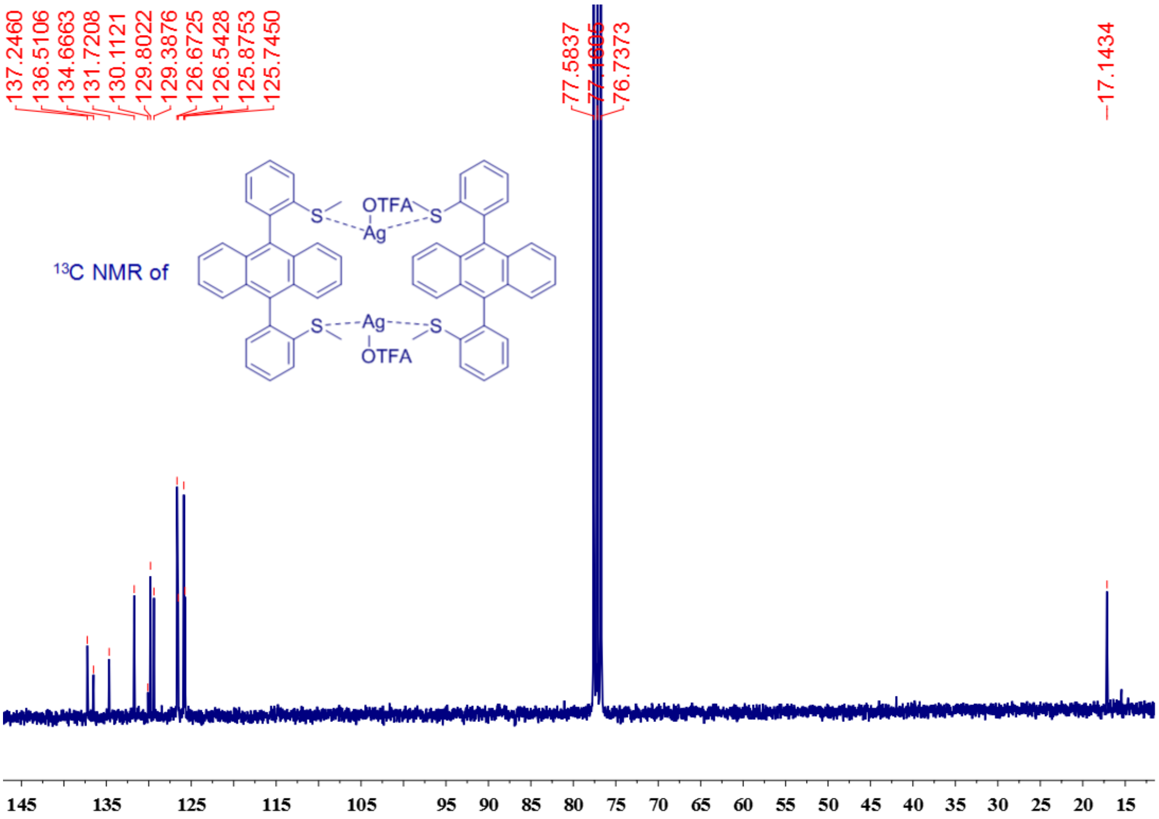

137.2460
136.5106
134.6663
131.7208
130.1121
129.8022
129.3876
126.6725
126.5428
125.8753
125.7450
77.5837
76.7373
17.1434
13C NMR of
OTFA
Ag
S
S
Ag
OTFA
S
S
145
135
125
115
105
95
90
85
80
75
70
65
60
55
50
45
40
35
30
25
20
15


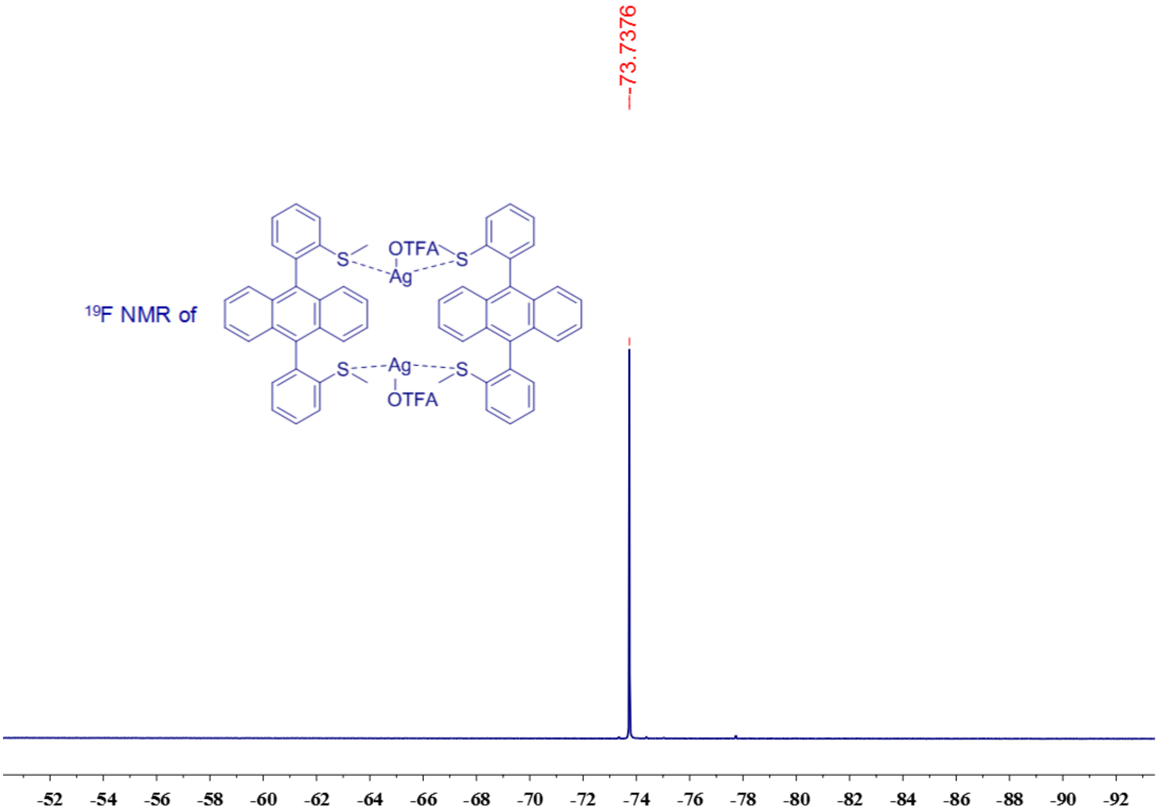

-73.7376
19F NMR of
OTFA
Ag
S
S
Ag
OTFA
S
S
-52
-54
-56
-58
-60
-62
-64
-66
-68
-70
-72
-74
-76
-78
-80
-82
-84
-86
-88
-90
-92

Complex **1c**

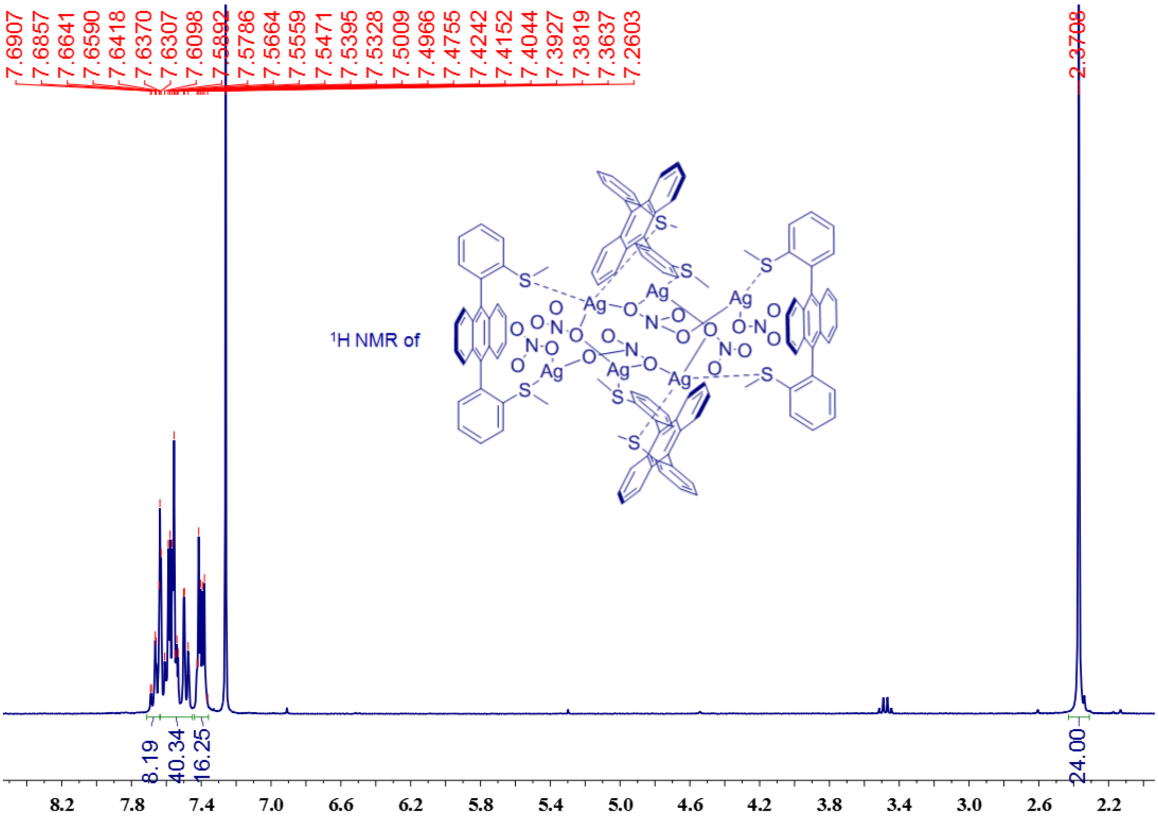

7.6907
7.6857
7.6641
7.6590
7.6418
7.6370
7.6307
7.6098
7.5892
7.5786
7.5664
7.5559
7.5471
7.5395
7.5328
7.5009
7.4966
7.4755
7.4242
7.4152
7.4044
7.3927
7.3819
7.3637
7.2603
2.3708
¹H NMR of
8.19
40.34
16.25
24.00
8.2
7.8
7.4
7.0
6.6
6.2
5.8
5.4
5.0
4.6
4.2
3.8
3.4
3.0
2.6
2.2


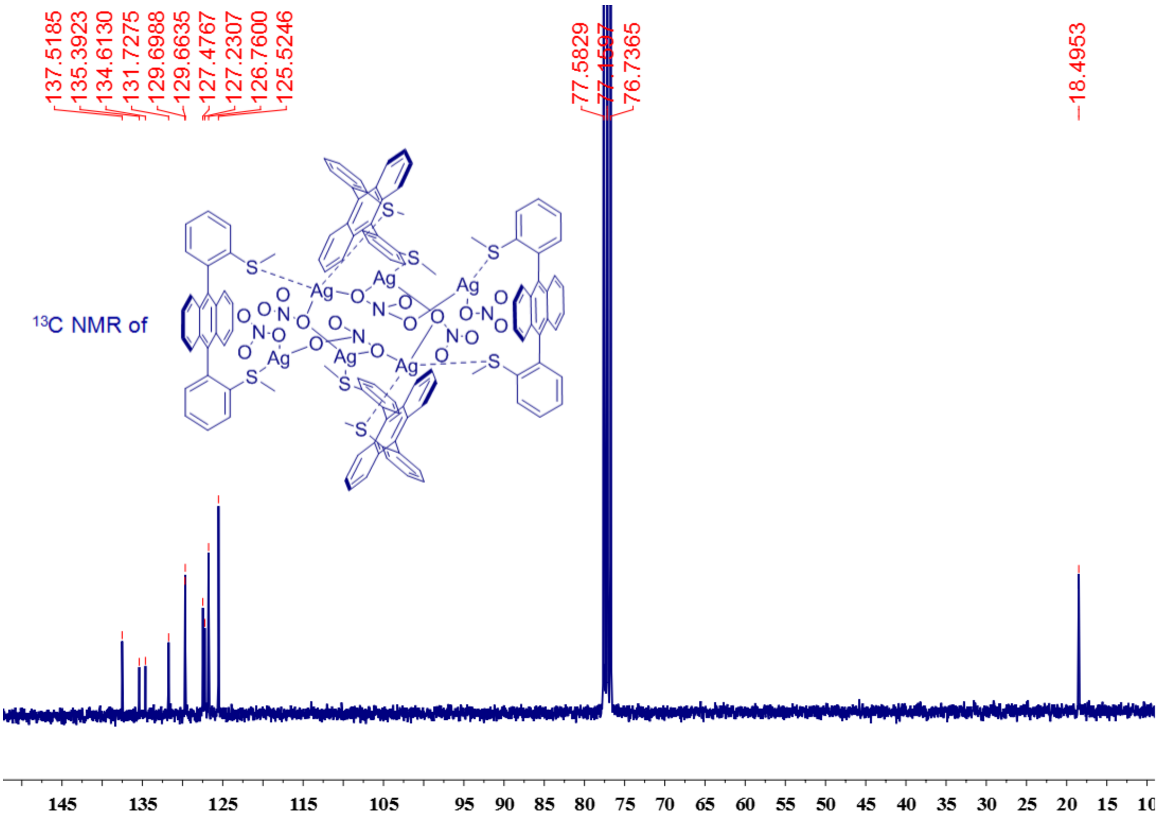

137.5185
135.3923
134.6130
131.7275
129.6988
129.6635
127.4767
127.2307
126.7600
125.5246
77.5829
77.1597
76.7365
18.4953
¹³C NMR of
145
135
125
115
105
95
90
85
80
75
70
65
60
55
50
45
40
35
30
25
20
15

Complex **1d**

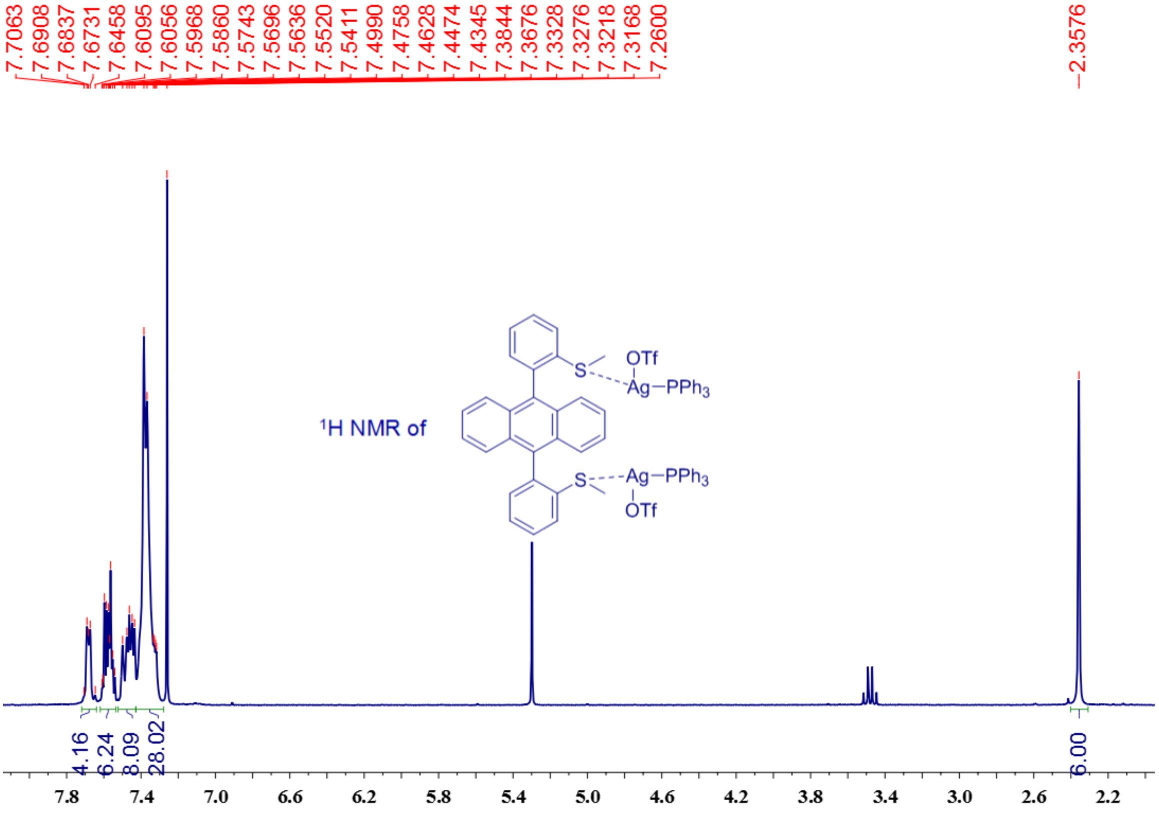

7.7063
7.6908
7.6837
7.6731
7.6458
7.6095
7.6056
7.5968
7.5860
7.5743
7.5696
7.5636
7.5520
7.5411
7.4990
7.4758
7.4628
7.4474
7.4345
7.3844
7.3676
7.3328
7.3276
7.3218
7.3168
7.2600
2.3576
1H NMR of
OTf
Ag−PPh3
S
4.16
6.24
8.09
28.02
6.00
7.8 7.4 7.0 6.6 6.2 5.8 5.4 5.0 4.6 4.2 3.8 3.4 3.0 2.6 2.2


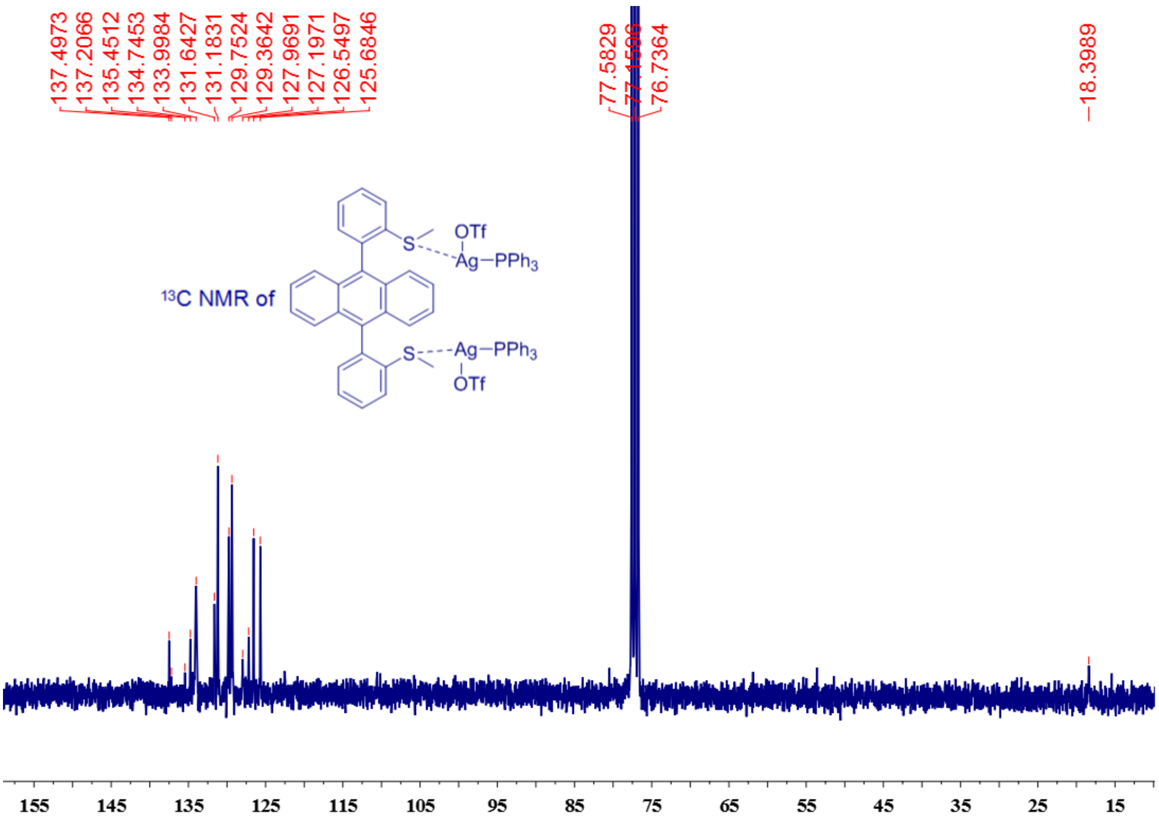

137.4973
137.2066
135.4512
134.7453
133.9984
131.6427
131.1831
129.7524
129.3642
127.9691
127.1971
126.5497
125.6846
77.5829
76.7364
18.3989
13C NMR of
OTf
Ag−PPh3
S
155 145 135 125 115 105 95 85 75 65 55 45 35 25 15

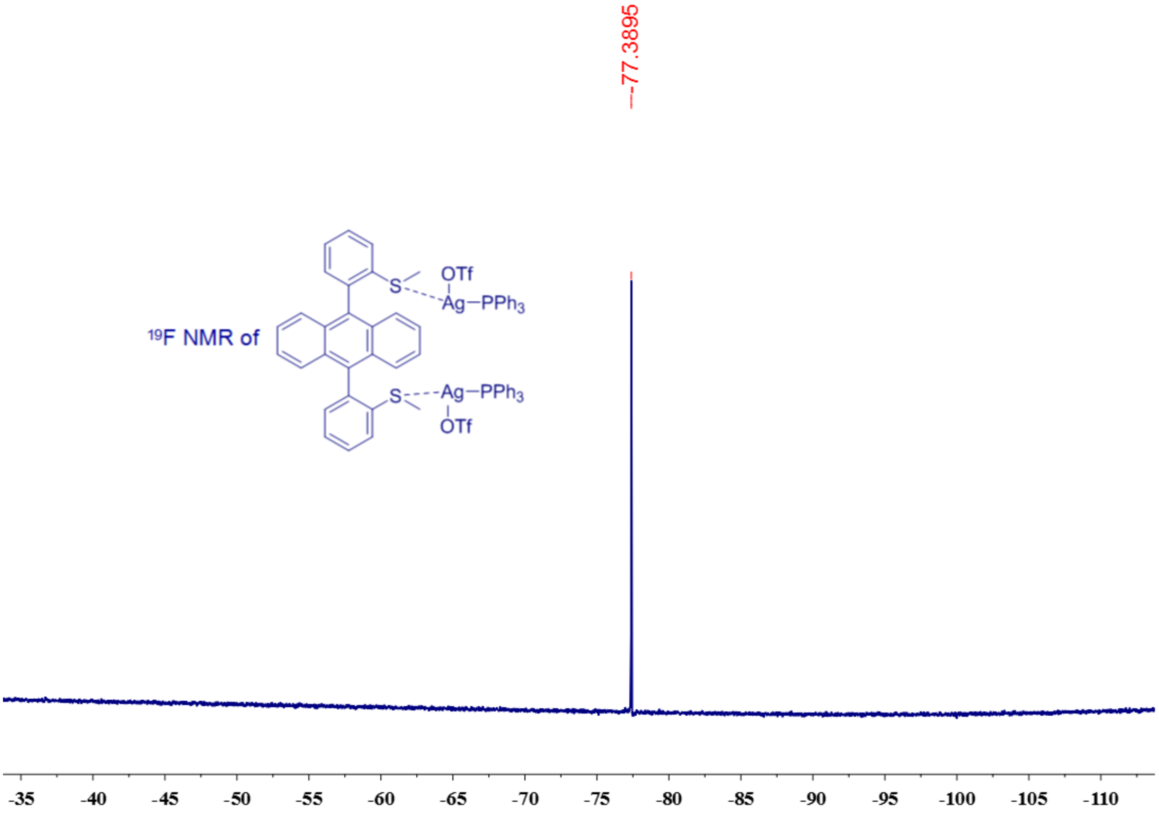


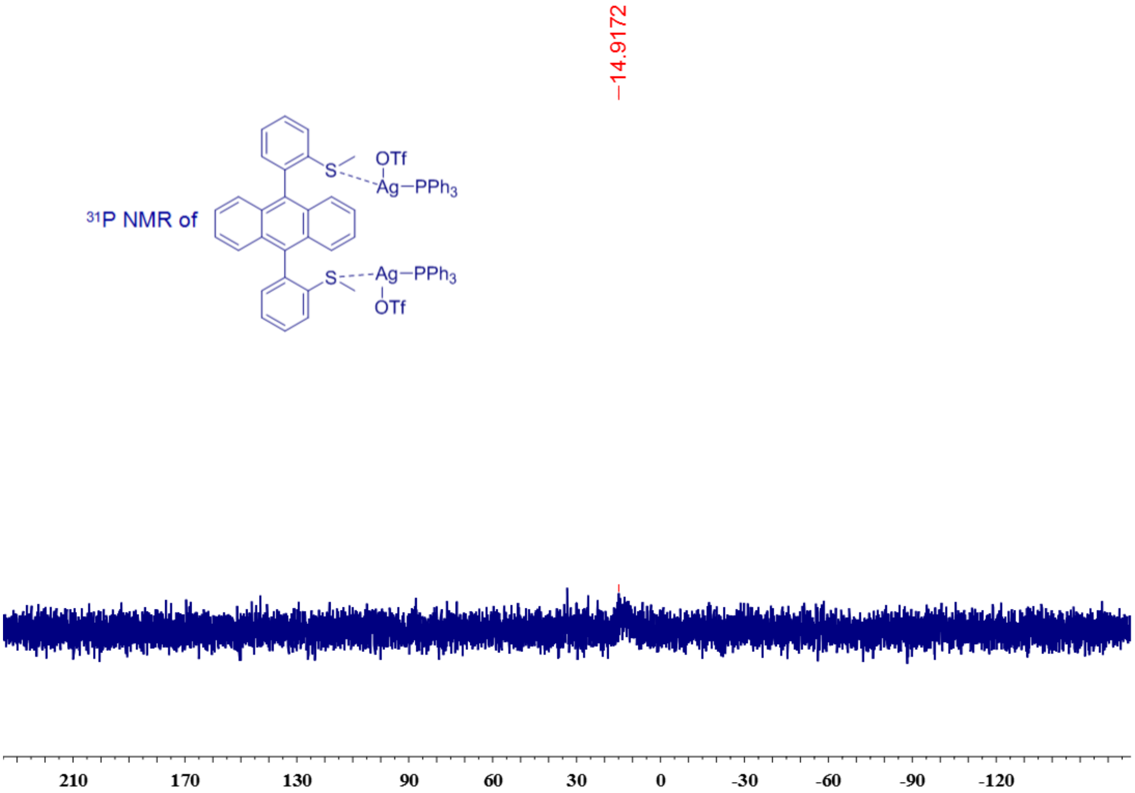


A rapid coordination/decoordination of triphenylphosphine on silver(I) on the NMR time scale may account for the absence of sharp signals on the $^{31}$P NMR spectrum. Such dynamic processes were previously described for silver(I)-phosphine complexes in solution.[8]

## 2D NMR spectra of silver complexes

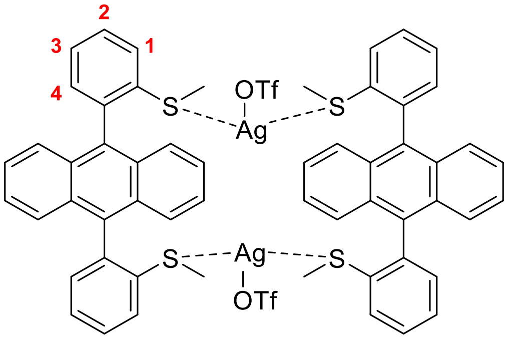

**1a**

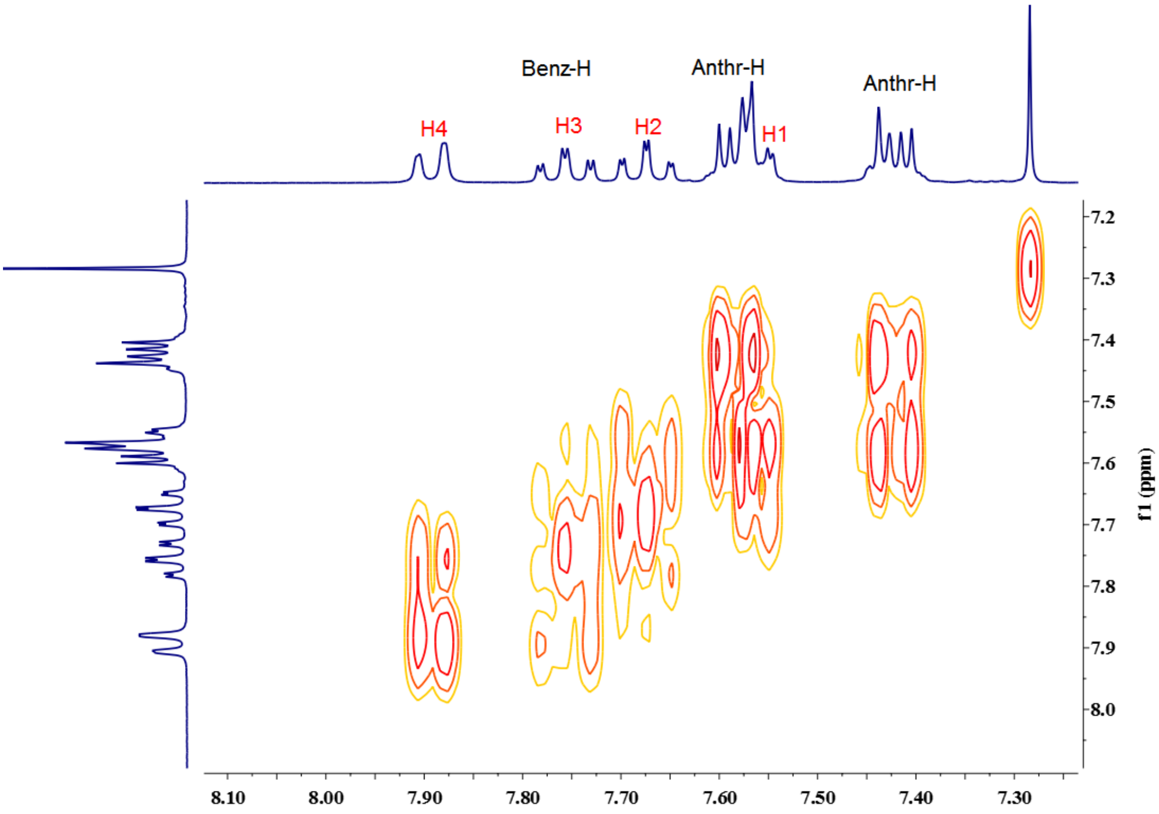


**Figure S22**. $^1$H-$^1$H gCOSY NMR spectrum of complex **1a**.

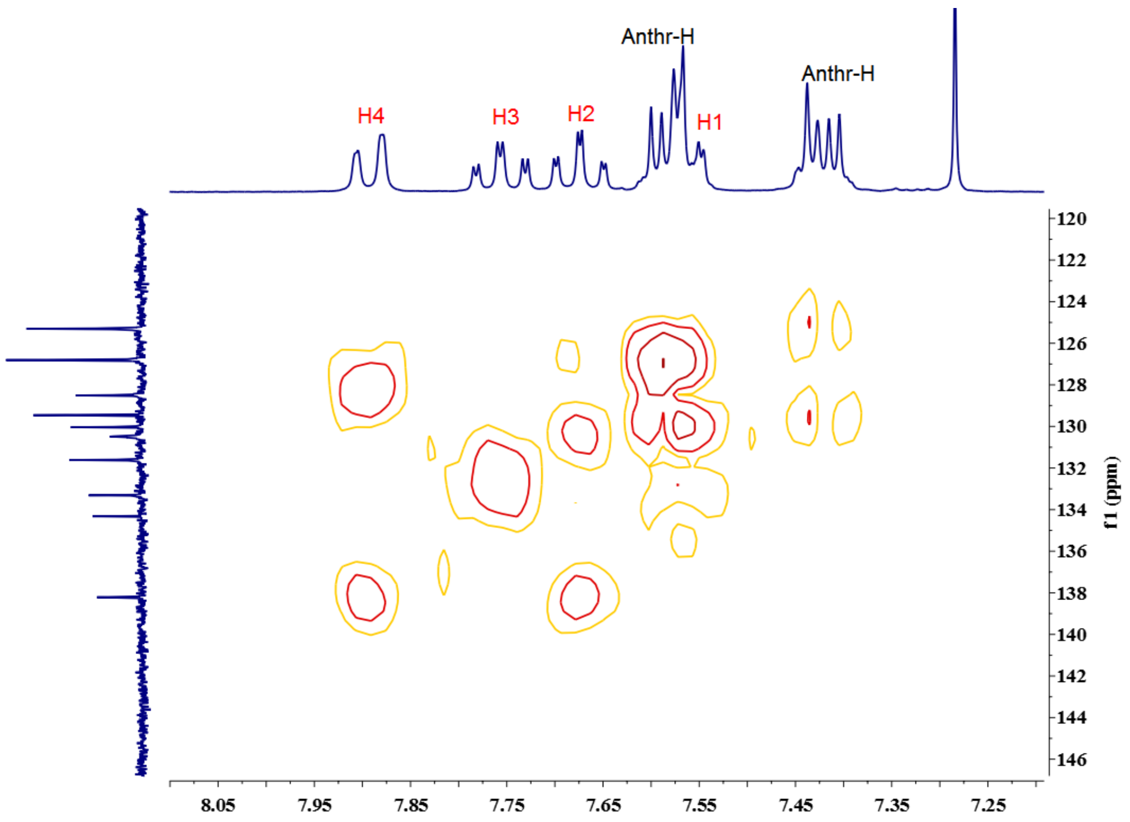


**Figure S23**. $^{1}H$-$^{13}C$ HSQC NMR spectrum of complex **1a**.

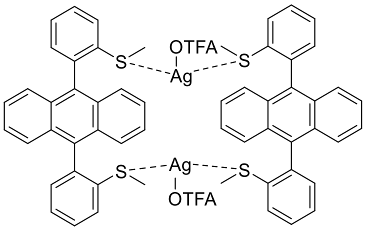


**1b**

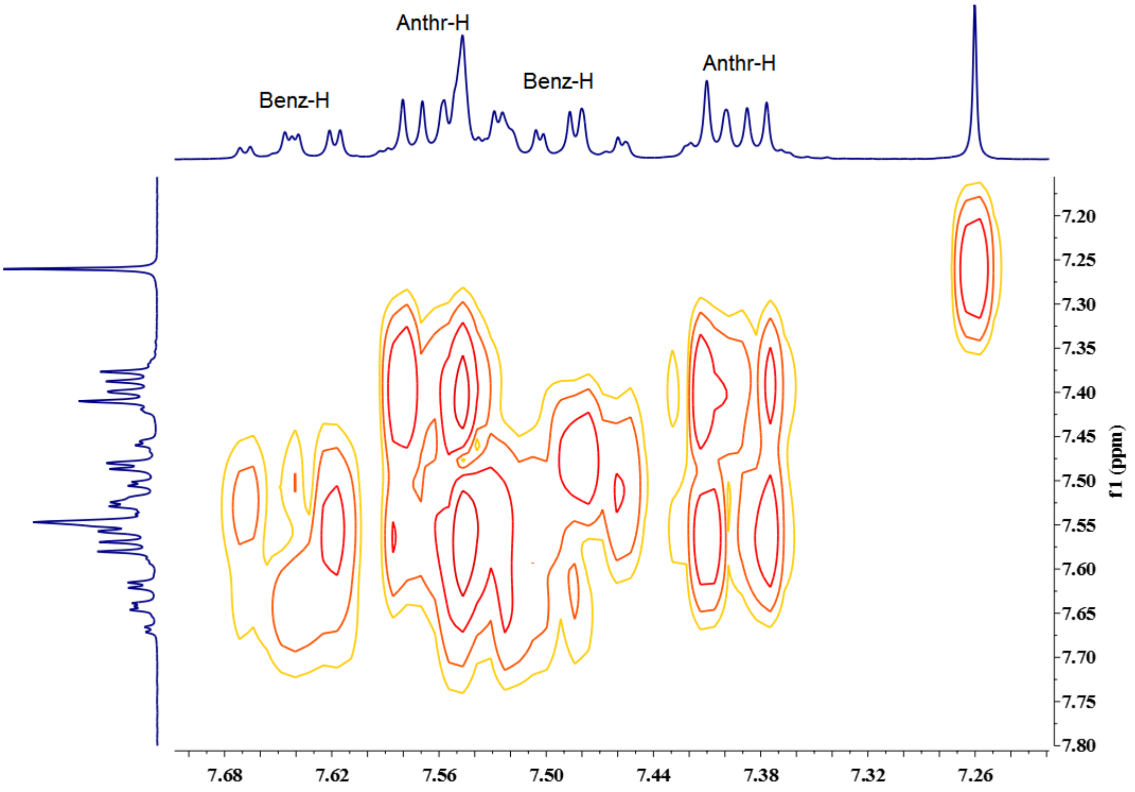


**Figure S24**. $^{1}H$-$^{1}H$ gCOSY NMR spectrum of complex **1b**.

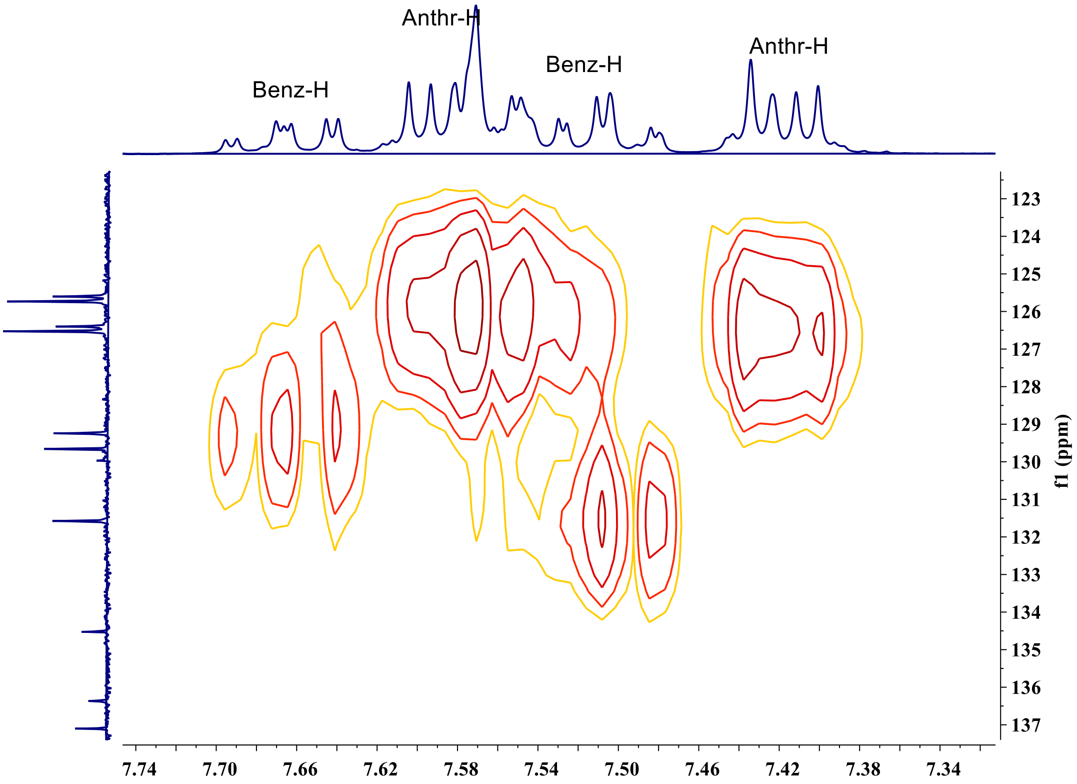


**Figure S25**. $^1$H-$^{13}$C HSQC NMR spectrum of complex **1b**.

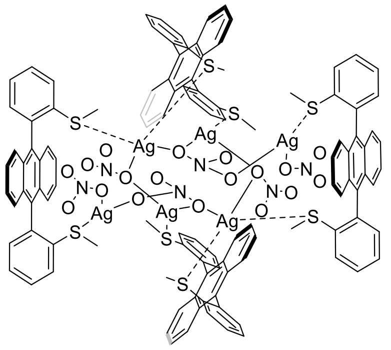

**1c**

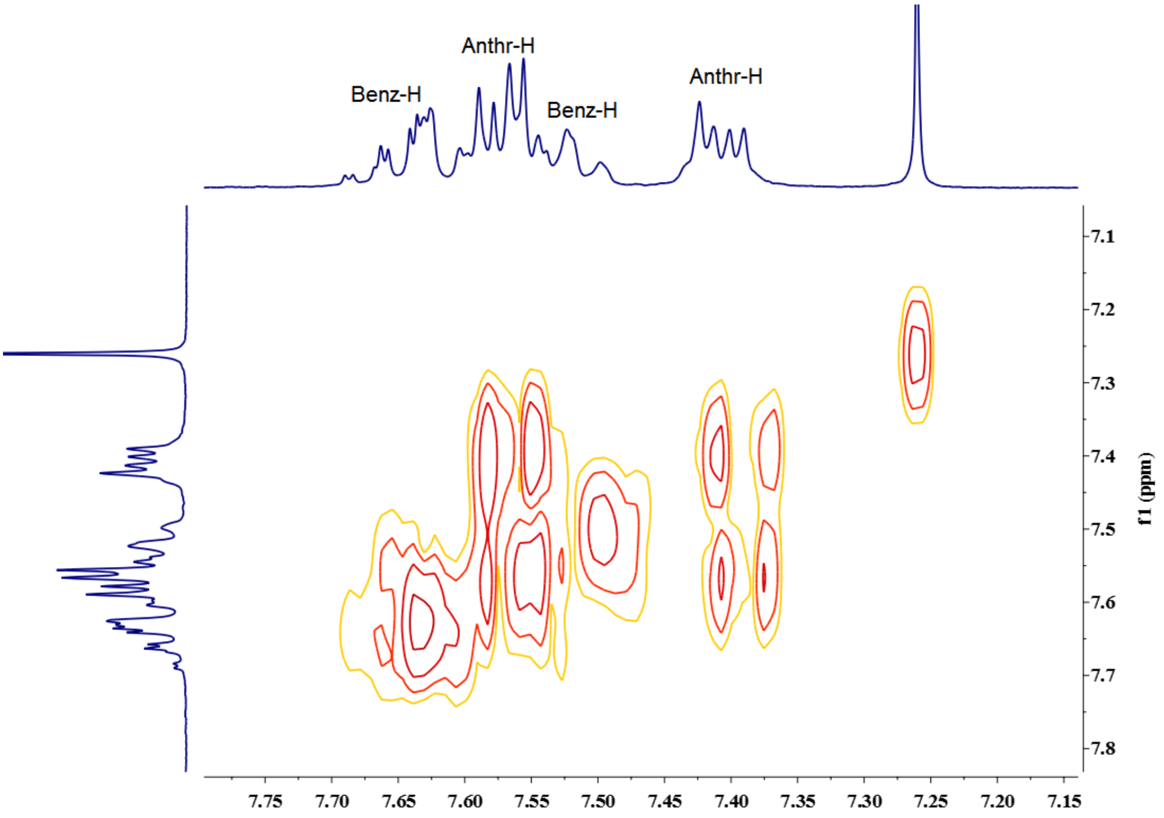


**Figure S26**. $^1$H-$^1$H gCOSY NMR spectrum of complex **1c**.

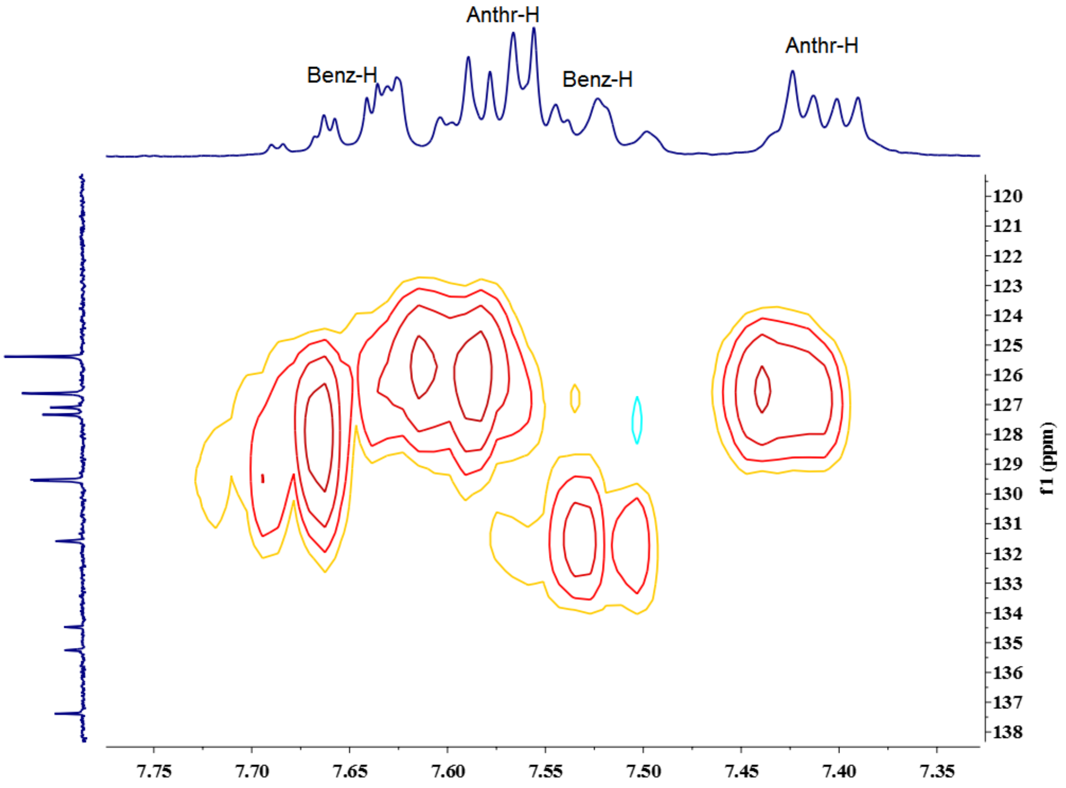


**Figure S27**. $^{1}H$-$^{13}C$ HSQC NMR spectrum of complex **1c**.

Compound **2**

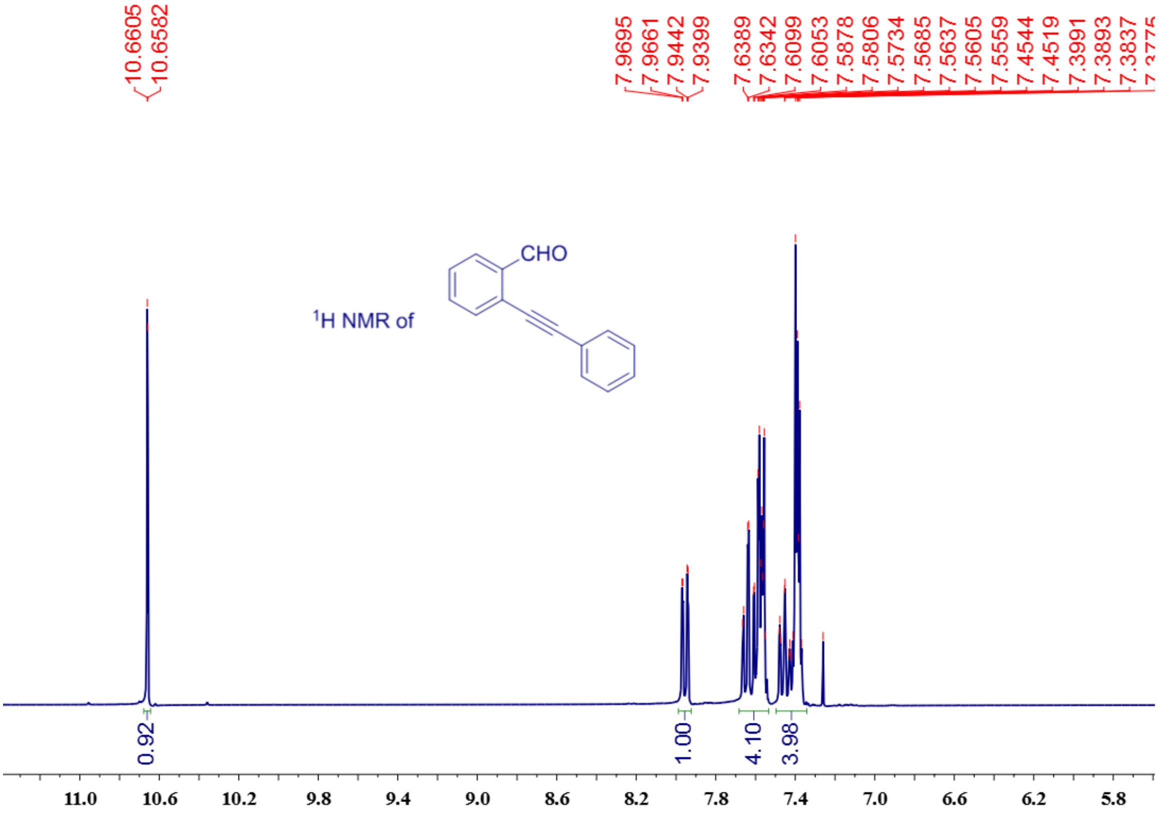
10.6605
10.6582
7.9695
7.9661
7.9442
7.9399
7.6389
7.6342
7.6099
7.6053
7.5878
7.5806
7.5734
7.5685
7.5637
7.5605
7.5559
7.4544
7.4519
7.3991
7.3893
7.3837
1H NMR of
CHO
0.92
1.00
4.10
3.98
11.0
10.6
10.2
9.8
9.4
9.0
8.6
8.2
7.8
7.4
7.0
6.6
6.2
5.8

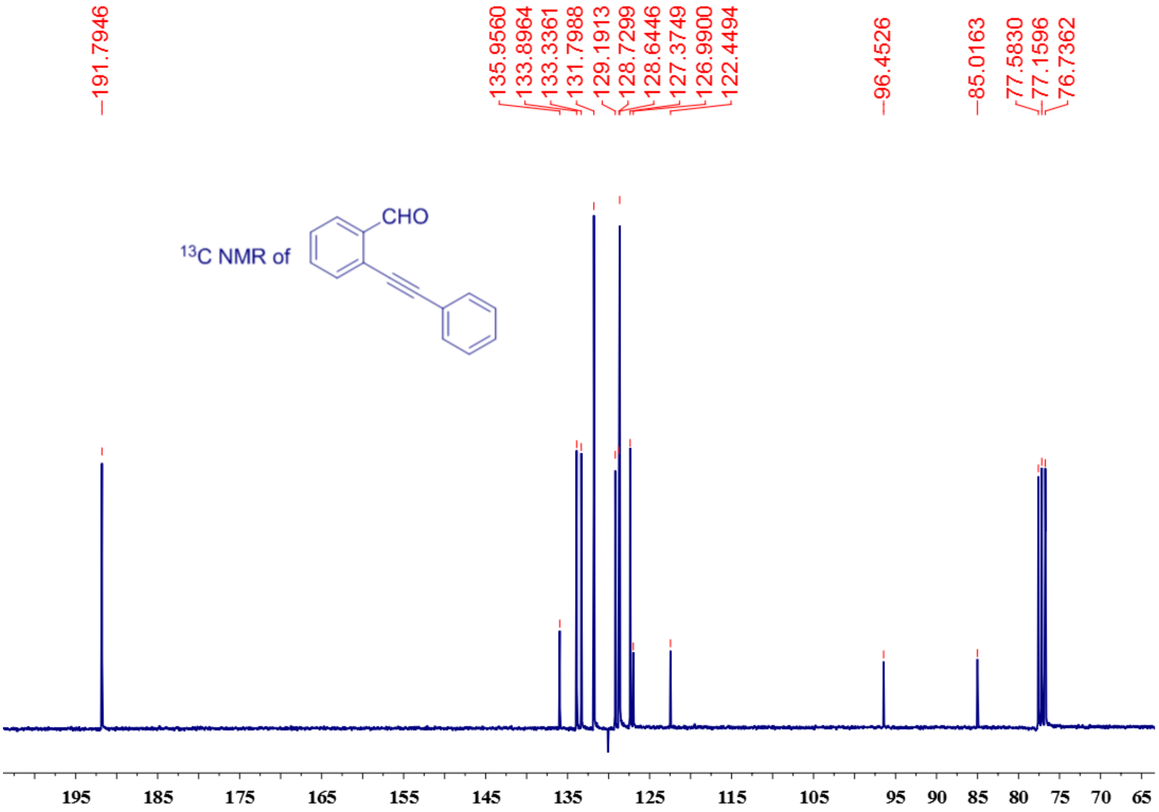
191.7946
135.9560
133.8964
133.3361
131.7988
129.1913
128.7299
128.6446
127.3749
126.9900
122.4494
96.4526
85.0163
77.5830
77.1596
76.7362
13C NMR of
CHO
195
185
175
165
155
145
135
125
115
105
95
90
85
80
75
70
65

Compound **3**

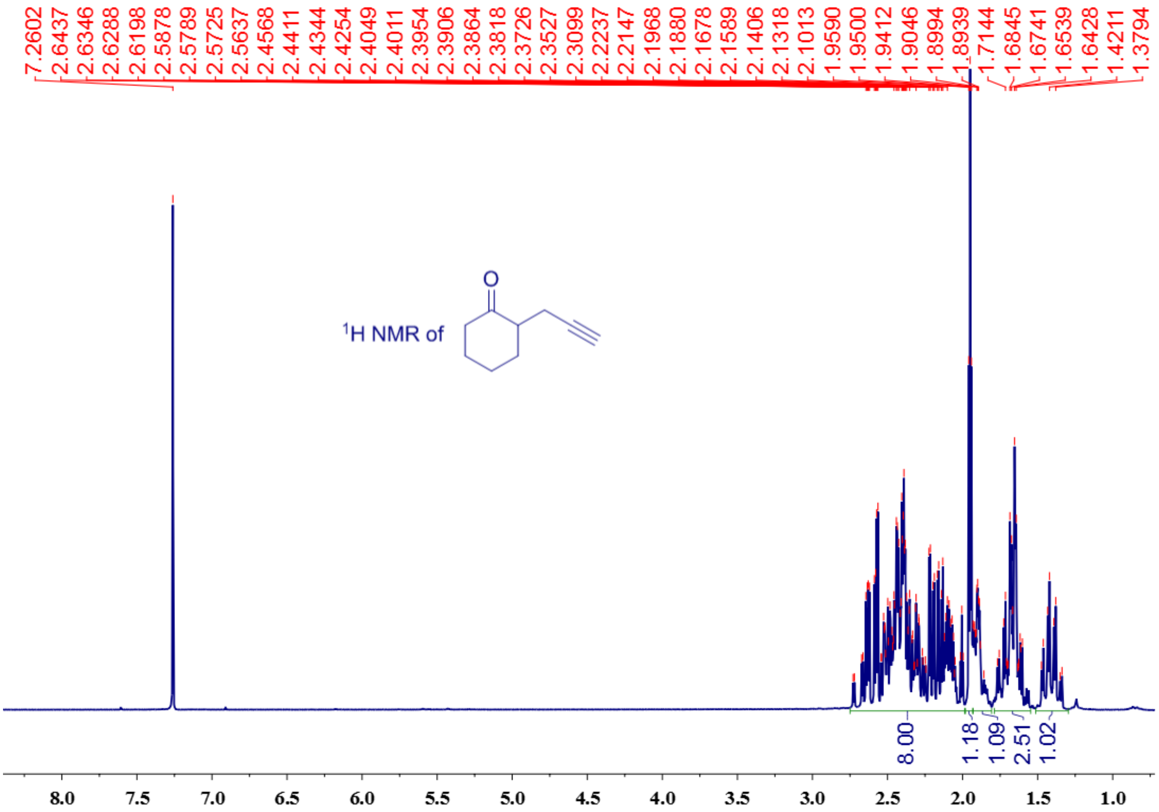

7.2602
2.6437
2.6346
2.6288
2.6198
2.5878
2.5789
2.5725
2.5637
2.4568
2.4411
2.4344
2.4254
2.4049
2.4011
2.3954
2.3906
2.3864
2.3818
2.3726
2.3527
2.3099
2.2237
2.2147
2.1968
2.1880
2.1678
2.1589
2.1406
2.1318
2.1013
1.9590
1.9500
1.9412
1.9046
1.8994
1.8939
1.7144
1.6845
1.6741
1.6539
1.6428
1.4211
1.3794
1H NMR of
O
8.00
1.18
1.09
2.51
1.02
8.0
7.5
7.0
6.5
6.0
5.5
5.0
4.5
4.0
3.5
3.0
2.5
2.0
1.5
1.0


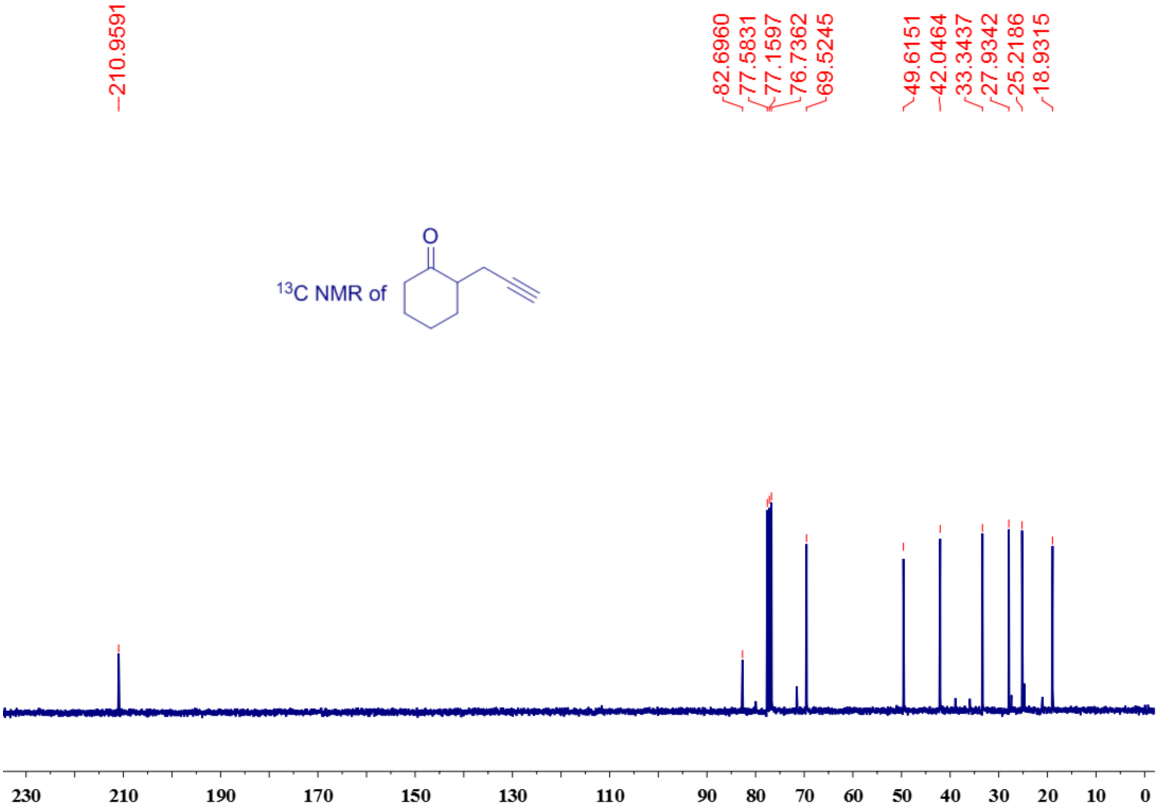

210.9591
82.6960
77.5831
77.1597
76.7362
69.5245
49.6151
42.0464
33.3437
27.9342
25.2186
18.9315
13C NMR of
O
230
210
190
170
150
130
110
90
80
70
60
50
40
30
20
10
0

Compound **4**

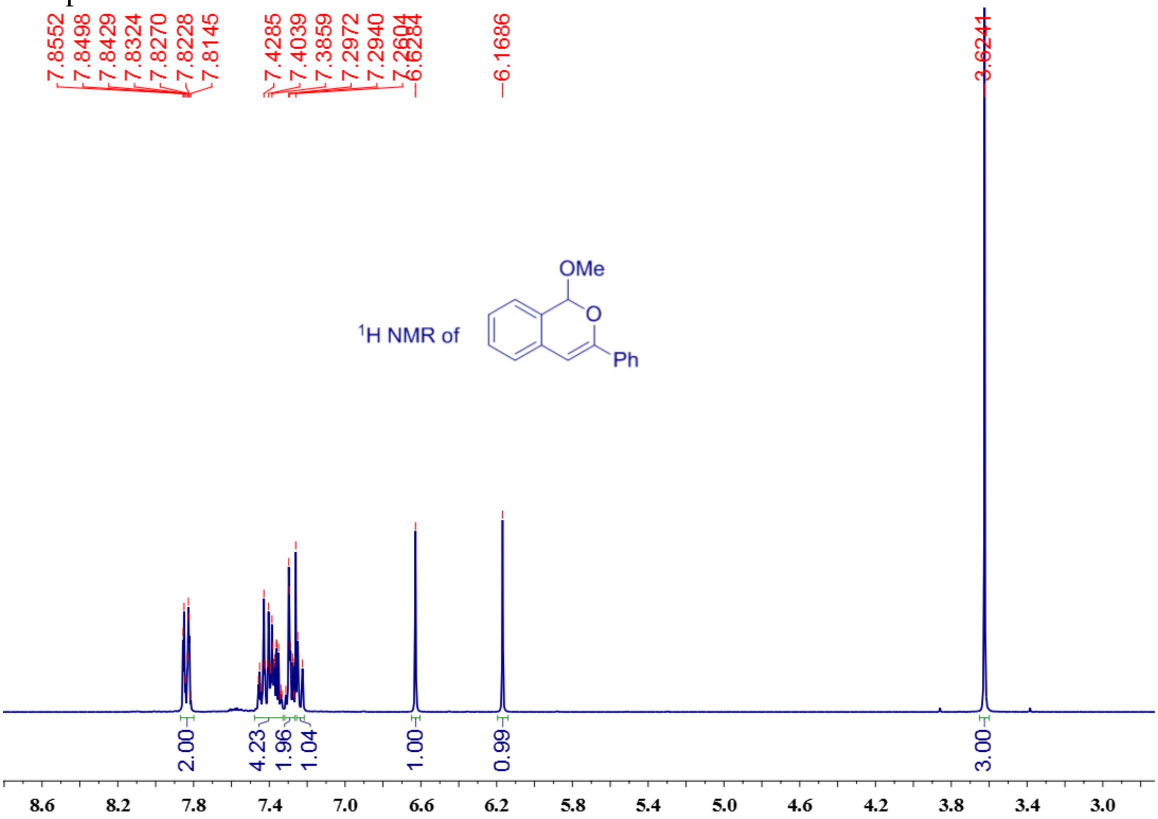
7.8552
7.8498
7.8429
7.8324
7.8270
7.8228
7.8145
7.4285
7.4039
7.3859
7.2972
7.2940
7.2604
6.6284
6.1686
3.6241
OMe
O
Ph
$^1$H NMR of
2.00
4.23
1.96
1.04
1.00
0.99
3.00
8.6
8.2
7.8
7.4
7.0
6.6
6.2
5.8
5.4
5.0
4.6
4.2
3.8
3.4
3.0

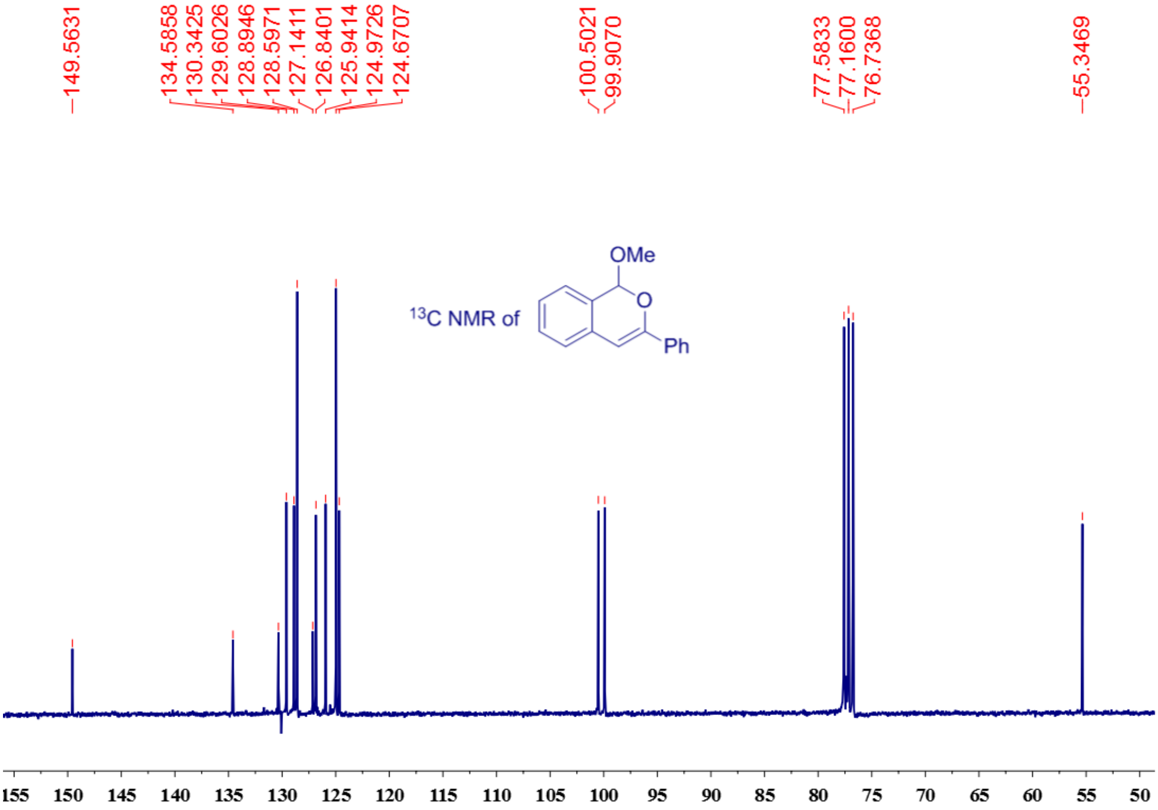
149.5631
134.5858
130.3425
129.6026
128.8946
128.5971
127.1411
126.8401
125.9414
124.9726
124.6707
100.5021
99.9070
77.5833
77.1600
76.7368
55.3469
OMe
O
Ph
$^{13}$C NMR of
155
150
145
140
135
130
125
120
115
110
105
100
95
90
85
80
75
70
65
60
55
50

Compound **5**

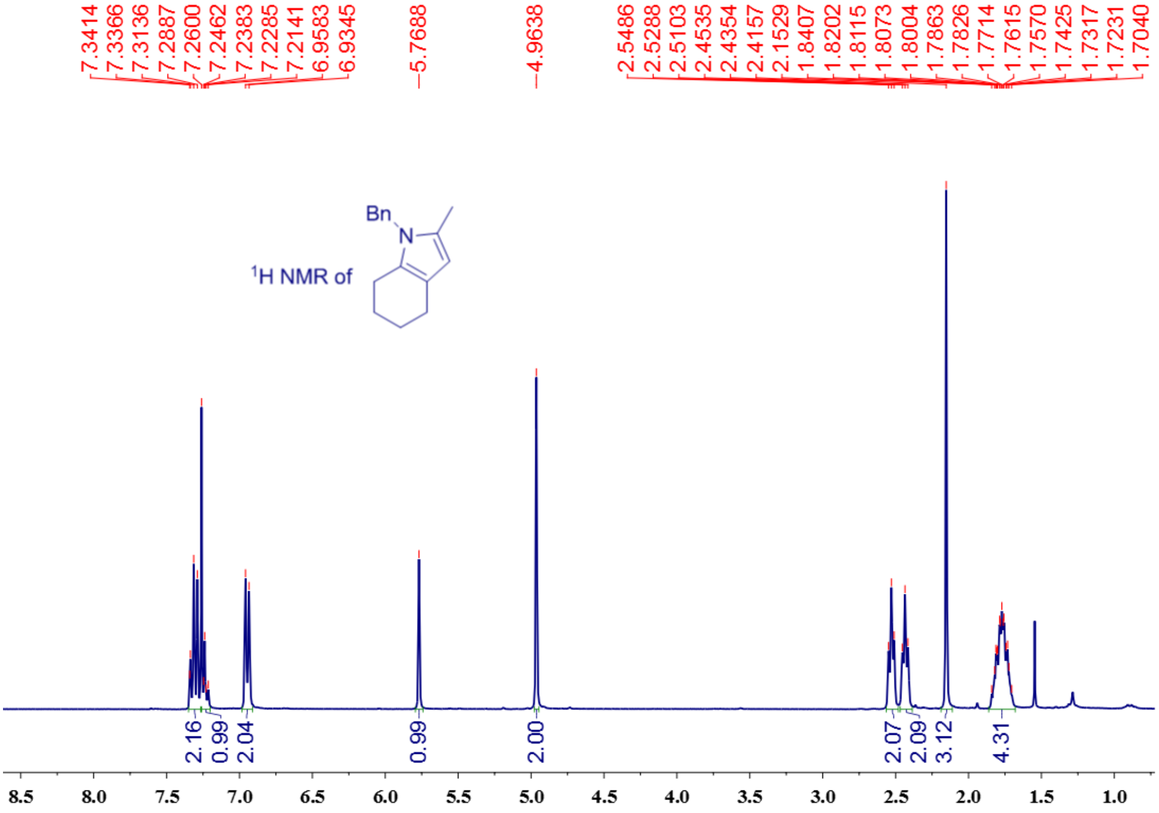

7.3414
7.3366
7.3136
7.2887
7.2600
7.2462
7.2383
7.2285
7.2141
6.9583
6.9345
5.7688
4.9638
2.5486
2.5288
2.5103
2.4535
2.4354
2.4157
2.1529
1.8407
1.8202
1.8115
1.8073
1.8004
1.7863
1.7826
1.7714
1.7615
1.7570
1.7425
1.7317
1.7231
1.7040
Bn
N
$^1$H NMR of
2.16
0.99
2.04
0.99
2.00
2.07
2.09
3.12
4.31
8.5
8.0
7.5
7.0
6.5
6.0
5.5
5.0
4.5
4.0
3.5
3.0
2.5
2.0
1.5
1.0


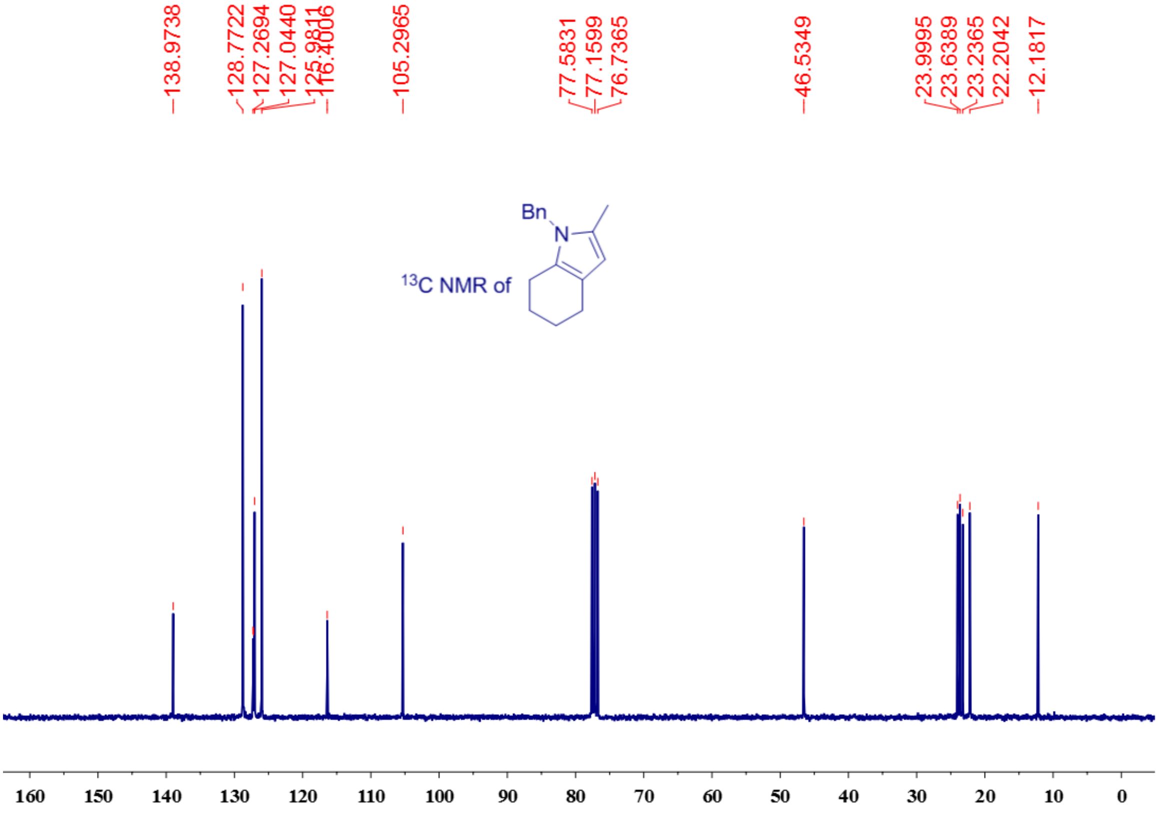

138.9738
128.7722
127.2694
127.0440
125.9811
116.4006
105.2965
77.5831
77.1599
76.7365
46.5349
23.9995
23.6389
23.2365
22.2042
12.1817
Bn
N
$^{13}$C NMR of
160
150
140
130
120
110
100
90
80
70
60
50
40
30
20
10
0

## References

[1] Prepared from $Ag_2O$ (1g, 4.32 mmol) and trifluoacetic acid (0.58 mL, 7.84 mmol) in water at room temperature. After 12 h, the suspension was filtered. The solid was suspended in diethyl ether (20 mL) and sonicated. Charcoal was added to the suspension. After filtration, the solution was concentrated and dried to afford $CF_3CO_2Ag$ (1.64 g, 95 % yield).

[2] a) T. G. Driver, K. A. Woerpel, *J. Am. Chem. Soc*. **2004**, *126*, 9993-10002; b) A. Izaga, R. P. Herrera, M. C. Gimeno, *ChemCatChem* **2017**, *9*, 1313-1321.

[3] a) L. Palatinus, G. Chapuis: SUPERFLIP - a computer program for the solution of crystal structures by charge flipping in arbitrary dimensions. *J. Appl. Cryst*., **2007**, *40*, 786-790; b) L. Palatinus, S. J. Prathapa, S. Van Smaalen, *J. Appl. Cryst.* **2012**, *45*, 575–580; c) G.M. Sheldrick, *Acta Cryst. Section A*, **2008**, *64*, 112–122.

[4] a) CrystalClear-SM Expert 2.1 (Rigaku, Jun 7th 2013) Software, Version 5.6.2.0, Tokyo, Japan; b) L. J. Farrugia, *J. Appl. Cryst*. **1999**, *32*, 837-838; c) C. F. Macrae, I. J. Bruno, J. A. Chrisholm, P. R. Edgington, P. McCabe, E. Pidcock, L. Rodriguez-Monge, R. Taylor, J. van de Streek, P. A. Wood, *J. Appl. Cryst*. **2008**, *41*, 466-470.

[5] a) T. Sakamoto, Y. Kondo, N. Miura, K. Hayashi, H. Yamanaka, *Heterocycles*. **1986**, *24*, 2311-2314; b) G. Dyker, W. Stirner, G. Henkel, *Eur. J. Org. Chem*. **2000**, 1433-1441.

[6] (a) B. M. Trost, M. T. Rudd, *J. Am. Chem. Soc*. **2005**, *127*, 4763-4776; (b) T. J. Harrison, J. A. Kozak, M. Corbella-Pane, G. R. Dake, *J. Org. Chem*. **2006**, *71*, 4525-4529.

[7] a) T. Godet, C. Vaxelaire, C. Michel, A. Milet, P. Belmont, *Chem. Eur. J*. **2007**, *13*, 5632-5641; b) E. Parker, N. Leconte, T. Godet, P. Belmont, *Chem. Commun*. **2011**, *47*, 343-345; c) M. Dell'Acqua, B. Castano, C. Cecchini, T. Pedrazzini, V. Pirovano, E. Rossi, A. Caselli, G. Abbiati, *J. Org. Chem*. **2014**, *79*, 3494-3505.

[8] a) U. Létinois-Haldes, P. Pale, S. Berger, *Magn. Reson. Chem*. **2004**, *42*, 831-834; b) G. A. Bowmaker, C. Di Nicola, Effendy, J. V. Hanna, P. C. Healy, S. P. King, F. Marchetti, C. Pettinari, W. T. Robinson, B. W. Skelton, A. N. Sobolev, A. Tabacaru, A. H. White, *Dalton Trans*. **2013**, *42*, 277-291.
